# Outcome measurement instruments for the core outcome sets on genital gender-affirming surgery: the GenderCOS project

**DOI:** 10.1016/j.eclinm.2026.103907

**Published:** 2026-04-18

**Authors:** Matteo Angelini, Philippine J. Roijer, Marleen S. Vallinga, Thomas E. Pidgeon, Aline Ceulemans, Alex Bakker, Brenda Carrière, Tina Rashid, James Bellringer, Javier Belinky, Marlon Buncamper, Shane D. Morrison, Walter P. Bouman, Tim C. van de Grift, Mark-Bram Bouman, Margriet G. Mullender

**Affiliations:** a Department of Plastic, Reconstructive and Hand Surgery, Amsterdam UMC (Location VUmc) - Amsterdam University Medical Centre, Amsterdam, the Netherlands; b Amsterdam Public Health Institute, Amsterdam, the Netherlands; c Vita-Salute San Raffaele University, IRCCS San Raffaele Hospital, Milan, Italy; d Department of Plastic Surgery, University Hospital Birmingham, Birmingham, United Kingdom; e Department of Plastic Surgery, University Hospital Ghent, Ghent, Belgium; f Independent Scholar, Utrecht/’s Hertogenbosch, the Netherlands; g Department of Urology, St George’s University Hospital NHS Foundation Trust, London, United Kingdom; h Department of Gender Surgery, Parkside Hospital, Wimbledon, London, United Kingdom; i Guemes Hospital and Urological Center CDU, Buenos Aires, Argentina; j Division of Plastic and Reconstructive Surgery, Department of Surgery, University of Washington, Seattle, WA, United States of America; k Department of Urology, University of Washington, Seattle, WA, United States of America; l Nottingham Centre for Transgender Health, Nottingham, United Kingdom; m Department of Psychiatry and Medical Psychology, Zaans Medical Center, Zaandam, the Netherlands

**Keywords:** Core outcome set, Gender-affirming surgery, Outcome measurement instruments, Transgender health, Patient-reported outcomes

## Abstract

**Background:**

Outcome assessment in genital gender-affirming surgery (gGAS) has long been a heterogenous practice. Although two core outcome sets (COS) for masculinizing and feminizing gGAS have been previously established, the absence of standardized and validated outcome measurement instruments (OMIs) limits consistent reporting. The second phase of the GenderCOS project aimed to identify, evaluate, and recommend OMIs to standardize outcome assessment and facilitate adoption of the COS in gGAS research, ultimately enabling comparability and evidence synthesis.

**Methods:**

The project followed the Core Outcome Measures for Effectiveness Trials (COMET) initiative standards and the COnsensus-based Standards for the selection of health Measurement INstruments (COSMIN) guideline for selecting OMIs for a COS. Phase 2 of the GenderCOS project was conducted from September 2024 to June 2025. Potential OMIs were identified through systematic reviews: PROSPERO: CRD42022347400 (inception to September 2023) and CRD42020223430 (inception to November 2020), clinical guidelines, and international expert consultations. All instruments relevant to at least one core outcome underwent quality and feasibility assessment. Consensus on the most appropriate OMIs and essential supplementary information was achieved through an international consensus process involving professional experts across multiple disciplines.

**Findings:**

A total of 380 potential OMIs were identified through systematic searches. After domain- and COS- matching, 152 patient-reported outcome measures (PROMs) and 53 clinical OMIs were evaluated on quality and feasibility aspects. The consensus process among professional experts in gender-care, resulted in measurement recommendations for 19 of the 20 unique core outcomes. These recommendations endorse the use of validated PROMs for the included patient-reported outcomes (PROs) and adherence to existing clinical guidelines for clinical outcomes and adverse events. For the remaining outcome relating to feminizing gGAS, a measurement recommendation was made for the subgroup that underwent vaginoplasty only. Development of an OMI suitable for all feminizing gGAS is also recommended.

**Interpretation:**

The GenderCOS provides the first consensus-based standardized measurement framework for core outcomes in gGAS. Its modular structure and inclusion of validated instruments enable harmonized reporting, data synthesis and evidence-based improvement in gGAS research.

**Funding:**

None received.

**Research in context**

**Evidence before this study**

A systematic search focusing on standardized core outcome measurement sets in genital gender-affirming surgery (gGAS) was conducted across the Core Outcome Measures in Effectiveness Trials (COMET) database, the COnsensus-based Standards for the selection of health Measurement INstruments (COSMIN) database, and PubMed, yielding no results. Systematic reviews conducted during phase 1, starting in 2021, and additional systematic searches up to June 2025 identified studies and guidelines reporting outcome measurement instruments (OMIs) used in gGAS, including both patient-reported and clinical outcomes. Searches were not limited by language or publication year and included all terms related to feminizing and masculinizing gGAS and outcome measurement tools. The available evidence demonstrated extensive heterogeneity in outcome assessment and substantial reliance on non-validated or ad-hoc tools, highlighting the absence of standardized measurement modalities and preventing meaningful comparison of core outcomes across studies.

**Added value of this study**

This study reports on the first international consensus process to identify specific validated OMIs and additional reporting requirements for the established core outcome sets in gGAS. It operationalizes the GenderCOS framework by defining not only *what* to measure but also *how* and *when* to measure outcomes most meaningful to both healthcare providers and transgender and gender-diverse (TGD) individuals. The resulting modular checklist integrates both clinical and patient-reported outcome measures that are validated in TGD populations. Areas requiring further development are also identified.

**Implications of all the available evidence**

The findings from this project provide a ready-to-use framework for standardized outcome assessment in gGAS. Consistent adoption will enhance comparability across studies, enable registry implementation, facilitate evidence synthesis, and inform future evidence-based surgical practice and decision-making. Ongoing evaluation of implementation feasibility and continued instrument development will ensure the COS remains responsive to emerging evidence and evolving practice.

## Introduction

In the last decade there has been a significant expansion in global provision and research activity in genital gender-affirming surgery (gGAS).^1^ gGAS includes a variety of surgical procedures performed for transgender and gender-diverse (TGD) individuals who wish to align their genital anatomy with their perceived gender.^2^ Differences in perceived outcomes, clinical results and adverse events between available surgeries and techniques are critical factors in surgical decision-making. However, outcome evaluation in gGAS remains heterogenous, limiting comparability of interventions and the formulation of evidence-based recommendations.^3^^,^^4^

Standardization of outcome measurement is essential to ensure collection of relevant data that are comparable and reproducible. Core outcome sets can ensure standardization by defining a minimum number of outcomes that should always be measured and reported in studies within a clinical research field. The Core Outcome Measures in Effectiveness Trials (COMET) and the COnsensus-based Standards for the selection of health Measurement INstruments (COSMIN) have been established to provide guidance for COS development and selection of the most appropriate assessment instruments.^5^ Two separate Core Outcome Sets (COS) were developed for feminizing and masculinizing gGAS as part of the GenderCOS project,^6^^,^^7^ following recommendations from COMET.^5^ These two COS comprise eleven and ten core outcomes respectively, including patient-reported outcomes, clinical outcomes and adverse events. The modular structure of both COS facilitates their applicability to different surgical interventions and techniques.

An additional essential step in the development and implementation of a COS is the identification and agreement on the most appropriate outcome measurement instruments (OMIs) and their associated reporting requirements. This enables consistent COS implementation in clinical practice and research. Hence, the aim of this second phase of the GenderCOS study was to identify, evaluate, and reach consensus on measurement instruments (*how*), timing of assessment (*when*), and additional reporting requirements for core outcomes in the two COS. The final recommendations are intended to facilitate standardized, high-quality outcome measurement in all future gGAS research.

## Methods

The larger GenderCOS project entails the development of two COS for masculinizing and feminizing gGAS. In phase 1, consensus was reached on the outcomes to be included in the two COS.^6^^,^^7^ The present paper describes phase 2 for both COS, aiming to reach consensus on how and when these core outcomes should be measured. The project commenced in September 2021 with the formation of an international study steering group (SSG) consisting of 16 members, professional experts in gGAS and lived experience experts, based in Europe, North America, and South America. The SSG was involved in all stages of the project. All members are listed as authors. Phase 1 was finalized in September 2024. During phase 2, the SSG convened digitally on 27 March 2025 and 24 June 2025, concluding phase 2.

The methodology was informed by the Core Outcome Set–STAndards for Development (COS-STAD), the COMET initiative handbook, the COSMIN initiative, and the joint COSMIN–COMET guideline on selecting OMIs for the two COS.^5^^,^^8^^,^^9^

### Registry entry/protocol

Phase 1 was prospectively registered in the COMET database (study numbers 2064 and 2067). The study protocol was published prior to the start of the phase 1 consensus process.^10^ A systematic review conducted as part of the project was registered in PROSPERO CRD42022347400, search period; inception to September 2023.^4^ Data from an earlier systematic review, registered in PROSPERO CRD42020223430, search period; inception to November 2020, were also shared and reused for this project.^3^

### Participants

For phase 2, the SSG identified professional experts (PEs) as the relevant stakeholder group, given the technical focus on evaluating OMIs and the need for clinical familiarity with existing assessment modalities. Eligible PEs were healthcare professionals with various levels of clinical and research experience, proficient in English, and able to provide informed e-consent. Candidates were identified through clinical networks, professional associations, and authorship of relevant peer-reviewed publications, and recruited via email.

### Procedures

**Fig. 1:**
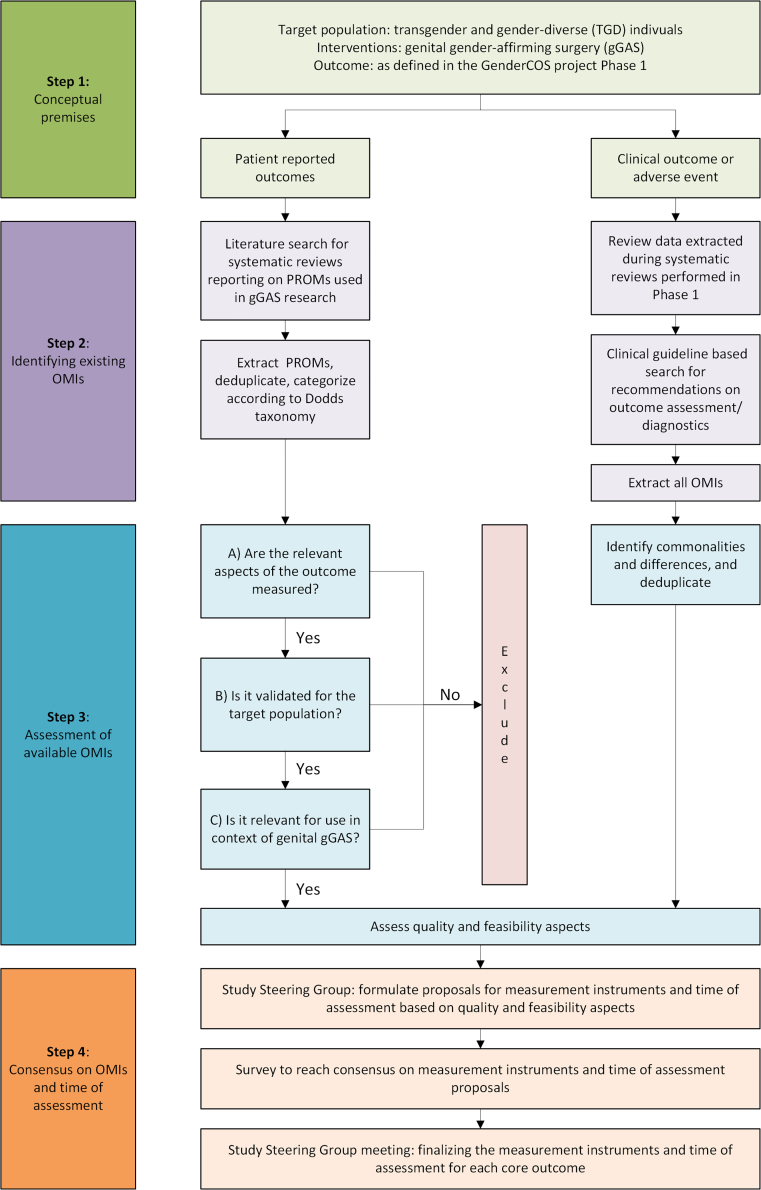
Overview of methods for Phase 2 of the GenderCOS project. OMIs: outcome measurement instruments; PROMs: patient-reported outcome measures; gGAS: genital gender-affirming surgery.

The development of recommendations on OMIs, timing of assessment, and additional reporting requirements for the two COS followed four predefined steps (Fig. 1).

#### Step 1: conceptual premises

The first step was to agree upon the construct (i.e., outcome or domain) to be measured and the target population. The target population comprised TGD individuals undergoing gGAS. The scope included all surgical interventions for masculinizing gGAS (i.e., phalloplasty or metoidioplasty, with or without urethral lengthening, scrotoplasty, and coronaplasty) and for feminizing gGAS (i.e., vaginoplasty, vulvoplasty, clitoroplasty, and labiaplasty), undertaken as either primary or revision procedures. The terms “feminizing” and “masculinizing” are used as established procedural descriptors and refer to surgical categories, rather than to gender identity or expression. During step 1 of the GenderCOS project, consensus was reached on the definition of each core outcome, its classification as patient-reported, clinical, or adverse event, and its scope.

#### Step 2: finding existing outcome measurement instruments

Phase 1 systematic reviews served as the primary source for identifying OMIs for both patient-reported and clinical/adverse event outcomes. Additional targeted literature searches were conducted for each core outcome to identify any instruments that might not have been captured previously.

Additional searches began with the COMET and COSMIN database to locate systematic reviews reporting on patient-reported outcome measures (PROMs) used in gGAS. Each systematic review identified was then subjected to backward citation tracking (screening its reference list) and forward citation tracking (screening articles that cited it) to identify other relevant systematic reviews. PROMs reported in the identified studies were extracted, deduplicated, and categorized according to the Dodds taxonomy.^11^ Only taxonomy categories and individual PROMs relevant to COS core outcomes were retained. For each retained PROM, the original instrument publication was reviewed to verify its characteristics. The recently published GENDER-Q (April 18, 2025) was also included, with permission from its developers.^12^

For clinical and adverse event outcomes, supplementary targeted searches were conducted to determine whether any expert-recommended OMIs existed. These included searches of professional organization websites—such as the WPATH Standards of Care version 8,^2^ and relevant guidelines from plastic surgery, urology, gynaecology, and abdominal surgery societies—for clinical guidelines addressing relevant outcomes. Relevant documents encountered opportunistically were also reviewed. Identified OMIs were compared across sources to determine commonalities and differences in recommendations. For each instrument, we documented which organizations or guidelines endorsed it, then removed duplicates and merged equivalent instruments to create a consolidated list for each domain. Clinical OMIs applicable to one or more core outcomes were retained.

All identified OMIs were categorized according to the COS outcome(s) they potentially address.

#### Step 3: assessing and preselecting instruments

PROMs were first screened against three content validity criteria: A) whether the instrument captures the outcome construct; B) whether the instrument had been validated for the target population (TGD individuals; or, in the case of more general outcomes, validated in the general population but not specifically cisgender men or women); and C) whether the instrument was relevant for use in gGAS. This initial screening was performed independently by two members of the study team, with discrepancies discussed and resolved by consensus. PROMs not meeting all three criteria were excluded from further consideration. Remaining instruments were reviewed for the quality of their development and validation processes, ensuring they were developed and validated in accordance with scientific standards and established methodology. When reported, evidence on sensitivity to change in TGD populations was evaluated The remaining PROMs were also assessed on feasibility aspects: applicability to the predefined scope for the core outcome, respondent burden (low, moderate, high), the extent of target population involvement in development (none, moderate, high), licensing requirements, costs and, recommended time of assessment (retrieved from the instrument manual when available, or, if not stated, from common usage in the literature).

For clinical outcome instruments, quality considerations included endorsement in professional guidelines, consistency with accepted clinical practice, and, when available, supporting evidence regarding validity or reliability. Assessment of clinical instruments was conducted by the same study team members and reviewed within the SSG to ensure clinical relevance and consistency. Feasibility aspects considered included: burden on both the patient and the administrator (low, moderate, or high); whether the measure was part of routine care; and costs.

The most suitable OMIs for each core outcome were listed, and instances where no suitable OMI existed were identified. Subsequent discussions among clinical and research experts were held to refine concept proposals for each outcome. Feasibility, completeness, and potential duplication of measurements across outcomes were evaluated during an online SSG meeting, after which a ‘draft OMI proposal’ was developed for each core outcome. Each proposal specified the recommended OMI, the timing of outcome assessment and any additional reporting requirements (e.g., scoring instructions, diagnostic criteria, classifications).

#### Step 4: consensus process

In Step 4, a structured online survey was first administered to PEs to determine agreement on the proposed OMIs. These proposals were circulated via a Google Forms survey, which was open for six weeks, with two reminders sent by email. Participants could choose to complete the survey for feminizing gGAS, masculinizing gGAS, or a combined version, depending on their self-identified expertise. The first part of the survey involved acquiring e-consent and collecting demographic data (gender identity, country, age, profession, years of professional experience, self-assessed clinical and/or research experience). Participants could also indicate whether they wanted to be acknowledged in the final publication. Responses to each proposal were collected using a 3-point Likert scale (“agree,” “mostly agree but minor refinements,” “disagree”). For the latter two categories, participants were prompted to provide comments explaining their refinements or alternative suggestions. For descriptive purposes, agreement distributions across outcomes were summarized using the median and interquartile range (IQR) of the percentage selecting “agree” only. Consensus was defined a priori, in line with phase 1 criteria, as >75% of respondents selecting “agree” or “mostly agree but minor refinements.” If consensus was not reached through the survey, a consensus meeting was planned to discuss those items and finalize recommendations.

Following the consensus process, the SSG met on 24 June 2025 to review the results. Refinements to the recommendations were made where indicated by survey feedback.

Lastly, the authors of the GENDER-Q were contacted to identify previously field-tested scales or items that might align with the core outcomes for which no existing validated tool could be recommended. This decision was based on the extensive qualitative data directly retrieved from the population that informed the Q scales, the rigorous methodology employed, and the identification of GENDER-Q scales for several other core outcomes, allowing for a more coherent assessment strategy and reducing respondent burden. These factors made it likely that conceptually relevant constructs might have been explored in earlier versions and later omitted. In addition to the published GENDER-Q scales, permission was obtained from developers to review and, if appropriate, include relevant unpublished short forms or stand-alone items from the field-tested pool. These candidate PROMs were evaluated using the same predefined quality and feasibility criteria as other OMIs, although they were not re-circulated for a second consensus vote.

### Ethics

The GenderCOS Project received ethical approval from the Amsterdam UMC, location VUmc ethical board (Reference number: 2022.0102). Participants could only begin the survey after providing informed e-consent.

### Role of the funding source

No funding was received for this study.

## Results

### Step 1: conceptual premises

Two separate COS were developed for masculinizing genital gender-affirming surgery (mgGAS) and feminizing genital gender-affirming surgery (fgGAS) as defined in Phase 1. Tables 1 and 2 summarize core outcomes and definitions.

**Table 1:** The final core outcome set for feminizing genital gender-affirming surgery.^6^

Scope	#	Outcome	Type of outcome	Definition
Feminizing gGAS (all)	1	Health related quality of life	Patient reported outcome	Evaluation of the patient's health-related quality of life, considering biological, psychological and social functioning, after genital gender-affirming surgery.
	2	Genital gender congruence	Patient reported outcome	The degree of experienced congruence between one's gender identity and one's physical genitals.
	3	Satisfaction with surgical result	Patient reported outcome	Patient assessed overall satisfaction with the outcome of genital surgery, in relation to expectations, desires, and gender affirmation.
	4	Satisfaction with aesthetic outcome	Patient reported outcome	Satisfaction with the aesthetic result of the surgically created vulva.
	5	Satisfaction with neo-genital sexual function	Patient reported outcome	Satisfaction with how the surgically created genitals function sexually.
	6	Erogenous sensibility of the genitals	Patient reported outcome	Ability to experience a sexually pleasurable or arousing sensation in the genital area.
	7	Additional surgery	Clinical outcome	The need for additional surgery to deal with complications, suboptimal outcomes, perceived problems or non-achieved desired results following previous genital gender affirming surgery.
Adverse events of vaginoplasty with depth	8	Loss of neovaginal tissue lining	Adverse event	Proportion of the tissue used for lining the surgically created vaginal canal that has been lost due to ischemia. infection. or other causes.
	9	Neo-vaginal stenosis	Adverse event	Narrowing of the surgically created neovaginal canal resulting in decreased functional vaginal depth and/or width causing symptoms in the patient.
	10	Stricture of the neovaginal introitus	Adverse event	Narrowing of or the presence of skin bridges at the surgically constructed opening of the neovagina.
	11	Rectovaginal fistula	Adverse event	Abnormal connection between the surgically constructed neo-vagina and the rectum.

gGAS: genital gender-affirming surgery.

**Table 2:** The final core outcome set for masculinizing genital gender-affirming surgery.^7^

Scope	#	Outcome	Type of outcome	Definition
Masculinizing gGAS (all)	1	Sensibility in the neo-phallus	Patient reported outcome	Degree of experienced sensation (i.e., pressure, touch, sexual, temperature) in the surgically constructed penis.
	2	Additional surgery	Clinical outcome	Additional surgical intervention needed to deal with complications, suboptimal outcomes, perceived problems or non-achieved desired results following previous genital gender affirming surgery.
	3	Ability to achieve orgasm	Patient reported outcome	Ability to experience a sexual climax.
	4	Sexual wellbeing	Patient reported outcome	A person's state of physical, emotional, mental and social wellbeing in relation to sexuality.
	5	Satisfaction with neo-genital aesthetic result	Patient reported outcome	Satisfaction with the aesthetic result of the surgically created phallus and, if applicable, scrotum.
Urethral lengthening surgery	6	Neo-urethral fistula	Adverse event	Unnatural connection between the surgically created urethra and another adjacent open space within or outside the body.
	7	Neo-urethral stricture	Adverse event	Narrowed segment of the surgically constructed urethra due to fibrosis and cicatrization of the urethral, associated with obstructive voiding symptoms.
	8	Ability to void in a standing position	Patient reported outcome	Ability to urinate while standing with a directable stream, through an unzipped fly without removing lower body clothing, without a urination device, allowing the use of public restrooms.
Phalloplasty	9	Flap necrosis of the neo-phallus	Adverse event	Proportion of the flap, used to surgically construct the phallus, that has died.
	10	Donor site morbidity	Patient reported outcome	Degree of experienced physical adverse effects (e.g., pain, sensation, tightness, discomfort, pulling) on the flap donor site.

gGAS: genital gender-affirming surgery.

### Step 2: identification of existing outcome measurement instruments

**Fig. 2:**
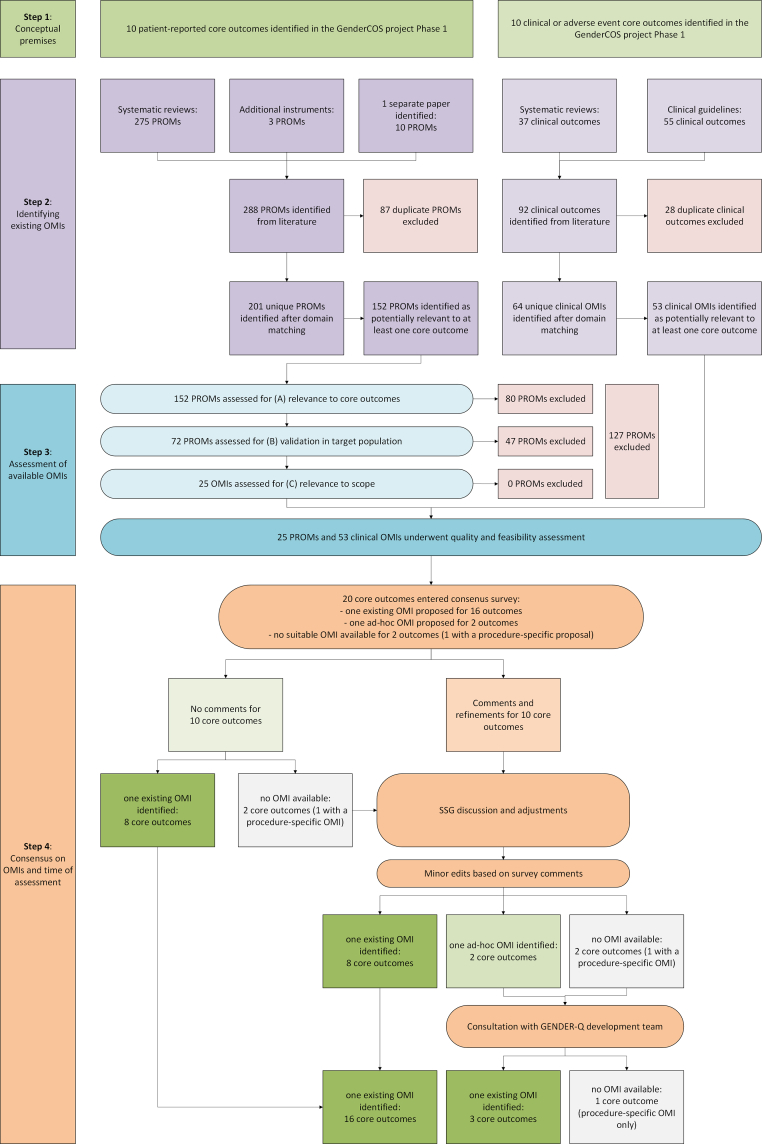
Flow diagram of outcome measurement instrument (OMI) identification, assessment, and selection process. OMIs: outcome measurement instruments; PROMs: patient-reported outcome measures.

The systematic search for PROMs historically used in gGAS identified nine systematic reviews,^3^^,^^4^^,^^13^, ^14^, ^15^, ^16^, ^17^, ^18^, ^19^ and one separate paper published after the included reviews.^12^ Additionally, three measurement tools were identified through forward citation tracking of the included studies. The search yielded 288 identified PROMs, of which 201 unique PROMs remained after duplicate removal and were catalogued according to the Dodds taxonomy.^11^ For clinical outcomes and adverse events, 64 unique instruments were identified through systematic literature review and relevant clinical guidelines after duplicate removal, largely representing expert-opinion-based measures endorsed in surgical and urological practice. A full inventory of identified instruments by outcome domain and source is provided in the Supplementary material. Overall, 152 PROMs and 53 clinical OMIs were considered potentially relevant to at least one core outcome and entered the quality and feasibility assessment. Fig. 2 presents the overall selection process.

### Step 3: quality and feasibility assessment

Using the predefined criteria, the applicability of the PROMs for each core PRO was evaluated. Of 152 PROMs assessed, 25 met all three criteria and proceeded to evaluation of their quality and feasibility, alongside 53 clinical instruments associated with at least one core outcome. Detailed quality and feasibility information for all PROMs and clinical instruments is provided in the Supplementary material.

### Step 4: selecting the outcome measurement instruments

#### Consensus survey

In line with the COSMIN-COMET guideline,^8^ a single OMI proposal was formulated for each core outcome based on results from previous steps. A total of 20 unique core outcomes–one of which applies to both COS–and 18 OMIs entered the consensus survey; two OMIs were ad-hoc proposals developed by the SSG when no suitable validated instrument existed. Ad-hoc OMIs were proposed only for outcomes with relatively simple constructs that could be captured through a direct straightforward measurement approach and were informed by similar constructs and field-tested items identified in the reviewed literature. For the remaining two outcomes in the feminizing COS–satisfaction with neogenital sexual function and erogenous sensibility of the genitals–no appropriate OMI applicable across the full feminizing scope could be identified and no ad-hoc proposal was formulated due to the complexity and multidimensional nature of these constructs. A partial recommendation was included for satisfaction with neovaginal sexual function, applicable only to the assessment of vaginoplasty with depth. A complete overview of the circulated survey, including the exact proposals for each outcome, is available in the Supplementary material.

Thirty-one experts participated in the feminizing COS consensus survey and 32 in the masculinizing COS survey, with 18 experts completing both surveys. Participants represented 12 countries and were predominantly experienced plastic surgeons and urologists, with the majority reporting over ten years of clinical experience and moderate-to-high research experience. An overview of survey participants’ demographics is presented in Table 3.

**Table 3:** Demographic data of feminizing and masculinizing COS survey participants.

Characteristic	Feminizing COS survey (n = 31)	Masculinizing COS survey (n = 32)
**Gender identity**		
Cisgender man	23 (74.2)	26 (81.3)
Cisgender woman	7 (22.6)	6 (18.8)
Transgender woman	1 (3.2)	0 (0)
**Country of residence**		
USA	9 (29.0)	8 (25.0)
Netherlands	5 (16.1)	7 (21.9)
Belgium	3 (9.7)	4 (12.5)
United Kingdom	4 (12.9)	4 (12.5)
Italy	3 (9.7)	3 (9.4)
Others (Germany, Argentina, Greece, Canada, Serbia, Spain, Sweden)	7 (22.6)	6 (18.8)
**Age**		
25–34 years	1 (3.2)	2 (6.3)
35–44 years	14 (45.2)	14 (43.8)
45–54 years	8 (25.8)	8 (25.0)
55–64 years	6 (19.4)	7 (21.9)
65+ years	2 (6.5)	1 (3.1)
**Years of experience in gender-affirming care**		
≤2 years	1 (3.2)	2 (6.3)
3–5 years	6 (19.4)	7 (21.9)
6–9 years	5 (16.1)	7 (21.9)
10–19 years	8 (25.8)	6 (18.8)
≥20 years	11 (35.5)	10 (31.3)
**Primary specialty**		
Plastic surgeon	18 (58.1)	16 (50.0)
Urologist	8 (25.8)	11 (34.4)
Gynaecologist	3 (9.7)	3 (9.4)
Trans health specialist (prescribing, dosing, and monitoring hormones, sexological and psychological advice/support)	1 (3.2)	1 (3.1)
Psychiatrist	1 (3.2)	1 (3.1)
**Clinical experience**		
Medical doctor not in residency training	0 (0)	1 (3.1)
Medical doctor in residency training	1 (3.2)	1 (3.1)
Recently completed specialist training	1 (3.2)	1 (3.1)
Early career specialist	3 (9.7)	3 (9.4)
Moderately experienced specialist	3 (9.7)	3 (9.4)
Experienced specialist	23 (74.2)	23 (71.9)
**Research experience (self-defined)**		
Limited	6 (19.4)	6 (18.8)
Moderate	6 (19.4)	5 (15.6)
Experienced	19 (61.3)	21 (65.6)

Median agreement (“agree” responses only) for feminizing gGAS outcomes was 83.9% (IQR 74.2–90.3%) and 87.5% (IQR 81.3–90.6%) for masculinizing gGAS. Consensus was reached for all 20 unique core outcome proposals, including all 18 OMIs and the two outcomes without a suitable OMI or with only a procedure-specific recommendation. As consensus was achieved for all items during the survey, no additional consensus meeting was required. While consensus was defined a priori as >75% selecting “agree” or “mostly agree but minor refinements,” the distribution between these two response options varied across outcomes and, for several adverse events, a substantial proportion of panelists selected “mostly agree”. This pattern was most apparent for items such as neo-urethral fistula (68.8% “agree,” 25.0% “mostly agree”) and loss of neovaginal tissue lining (61.3% “agree,” 38.7% “mostly agree”). Differing opinions on optimal measurement techniques primarily accounted for the lower rates of complete agreement observed for these outcomes, as shared in the accompanying quotes. An overview of survey results is presented in Table 4, and a complete inventory of agreement levels for each OMI and reasons for disagreement is provided in the Supplementary material.

**Table 4:** Summary of results from the consensus surveys on OMIs in feminizing and masculinizing COS.

Proposal for outcome	Agree, n (%)	Mostly agree, n (%)	Disagree, n (%)
**Feminizing COS (n = 31)**			
Additional surgery	27 (87.1)	4 (12.9)	0 (0)
Loss of neo-vaginal tissue lining	19 (61.3)	12 (38.7)	0 (0)
Neo-vaginal stenosis	23 (74.2)	7 (22.6)	1 (3.2)
Stricture of the neo-vaginal introitus	24 (77.4)	6 (19.4)	1 (3.2)
Rectovaginal fistula	23 (74.2)	8 (25.8)	0 (0)
Satisfaction with neogenital sexual function	27 (87.1)	4 (12.9)	0 (0)
Erogenous sensibility of the genitals	26 (83.9)	5 (16.1)	0 (0)
Genital gender congruence	26 (83.9)	4 (12.9)	1 (3.2)
Satisfaction with surgical result	29 (93.5)	2 (6.5)	0 (0)
Satisfaction with aesthetic outcome	29 (93.5)	2 (6.5)	0 (0)
Health-related quality of life	28 (90.3)	2 (6.5)	1 (3.2)
**Masculinizing COS (n = 32)**			
Additional surgery	28 (87.5)	4 (12.5)	0 (0)
Neo-urethral stricture	24 (75.0)	6 (18.8)	2 (6.3)
Neo-urethral fistula	22 (68.8)	8 (25.0)	2 (6.3)
Flap necrosis neo-phallus	28 (87.5)	4 (12.5)	0 (0)
Sensibility in the neo-phallus	29 (90.6)	1 (3.1)	2 (6.3)
Ability to achieve orgasm	30 (93.8)	2 (6.3)	0 (0)
Sexual well-being	29 (90.6)	3 (9.4)	0 (0)
Satisfaction with neo-genital aesthetic result	29 (90.6)	3 (9.4)	0 (0)
Donor site morbidity	28 (87.5)	4 (12.5)	0 (0)
Ability to void in a standing position	26 (81.3)	5 (15.6)	1 (3.1)

#### SSG meeting and final refinements

Survey findings informed final SSG discussions. Although consensus had been reached for all outcomes, ten OMIs showed either moderate consensus (i.e., ≤75% selecting “agree” but >75% selecting either “agree” or “mostly agree”) or generated substantial comments and were therefore reviewed in greater depth during the SSG meeting. These outcomes were selected for further discussion because the extent and nature of the comments indicated uncertainties or divergent interpretations that required clarification. Minor adjustments were made based on the feedback received. For two outcomes—erogenous sensibility in the genitals, and satisfaction with neogenital sexual function—the SSG confirmed the absence of a suitable existing instrument across the full feminizing gGAS spectrum and the need for future PROM development and validation.

In the final phase, the SSG consulted the GENDER-Q development team regarding the four outcomes for which either ad-hoc PROMs had reached consensus or no suitable OMI had been identified. For the two outcomes with ad-hoc tools, the developers indicated that previously field-tested and psychometrically evaluated items were available that corresponded to the same underlying constructs. These items had undergone the same qualitative development, cognitive testing, and field-testing procedures as the published scales, but were later removed during Rasch-based scale refinement for statistical or practical reasons rather than lack of content validity or population relevance. The overall field-test study included 601 participants across multiple countries. The 2-item “Satisfaction with urinating”, measuring the ability to void in a standing position, demonstrated acceptable reliability for a brief scale (Cronbach’s α = 0.93; PSI = 0.71), with both items showing ordered thresholds and individual fit to the Rasch model, although the global Rasch model fit statistic was significant (χ^2^ = 13.40; df = 4; p = 0.01). The 5-item “Female genital sensation” scale, applicable to erogenous sensibility of the genitals, showed acceptable model fit (χ^2^ = 45.46; df = 40; p = 0.25) and reliability (Cronbach’s α = 0.85; PSI = 0.77). For genital gender congruence, field-tested “Gender-affirmation questions” had poor fit within their original subscales and were removed during refinement. These items do not function as a multi-item scale but were retained as single stand-alone items due to their conceptual alignment with the agreed outcome construct and their prior qualitative development. These PROMs were not re-circulated for PE voting and were adopted by the SSG following internal review. However, no suitable additional tools could be identified to capture satisfaction with neogenital sexual function and this outcome therefore retains only a procedure-specific assessment recommendation. Nineteen OMIs were ultimately recommended for 20 unique core outcomes across the pre-defined scope. An overview of the complete consensus process is presented in Fig. 2.

### Final recommended assessment modalities

For the 12 PROs in the two COS, the PROMIS Global Health scale was selected for health-related quality of life in the feminizing COS, while GENDER-Q scales were selected for the majority of PROs in both the feminizing and masculinizing COS. Stand-alone questions and two additional independently functioning scales were formed from field test items that had been dropped from the GENDER-Q during item reduction and are recommended for the PROs genital gender congruence, erogenous sensibility in the genitals and ability to void in a standing position, respectively. One PRO–satisfaction with neogenital sexual function–had no validated PROM applicable to the full feminizing scope, and further research is required. The GENDER-Q Vagina scale can be used to assess the outcome in individuals who have undergone vaginoplasty with depth (i.e., the surgical creation of a vaginal canal, regardless of technique).

For clinical and adverse event outcomes, recommendations were aligned with international guidelines and adapted after expert feedback and SSG discussion: physical examination emerged as the most appropriate first-line evaluation for most outcomes, complemented by internal examination with speculum for vaginal complications, standardized uroflowmetry and imaging for urethral outcomes, and further imaging or endoscopic examination for inconclusive cases. Additional reporting information included further characterization of the adverse events, including extension, location, time of diagnosis and Clavien–Dindo classification for severity.^20^

Regarding the timing of outcome assessment, consensus was reached on recommending 12 months following primary surgery as the upper time point for assessing core outcomes in the two COS, with subsequent assessment at five-year intervals without upper limit of time. Clinical outcomes and adverse events occurring within the defined 12-month follow-up window should be reported and characterized according to the recommended assessment framework, irrespective of whether they have resolved by the time of assessment.

Tables 5 and 6 summarize the final recommended instruments and timing of assessment for each core outcome.

**Table 5:** Patient-reported outcomes for feminizing and masculinizing gGAS.

Scope	Outcome	Outcome assessment method	Required reporting
Feminizing gGAS	Health related quality of life	PROMIS scale: Global Health	Calculated T-score and Standard Error (SE) for scale.
	Genital gender congruence	GENDER-Q single items: Gender affirmation questions	Raw item score.
	Satisfaction with surgical result	GENDER-Q scale: Treatment outcome	Converted Rasch score for scale.
	Satisfaction with aesthetic outcome	GENDER-Q scales: Labia and Clitoris	Converted Rasch score for scale.
	Satisfaction with neo-genital sexual function	GENDER-Q scale: Vagina (only for surgeries including creation of a vaginal canal)	Converted Rasch score for scale.
	Erogenous sensibility of the genitals	GENDER-Q scale: Female genital sensation	Converted Rasch score for scale.
Masculinizing gGAS	Sensibility in the neo-phallus	GENDER-Q scale: Penis sensation	Converted Rasch score for scale.
	Ability to achieve orgasm	GENDER-Q scale: Orgasm	Converted Rasch score for scale.
	Sexual well-being	GENDER-Q scale: Sexual Well-being	Converted Rasch score for scale.
	Satisfaction with neo-genital aesthetic result	GENDER-Q scale: Penis	Converted Rasch score for scale.
		And, if applicable: GENDER-Q scale: Scrotum	
		And, if applicable: GENDER-Q scale: Glans	
Phalloplasty with or without urethral lengthening	Donor site morbidity	GENDER-Q scale: Donor site—Adverse effects	Converted Rasch score for scale.
Masculinizing gGAS with urethral lengthening	Ability to void in a standing position	GENDER-Q scale: Satisfaction with urinating	Converted Rasch score for scale.

**Table 6:** Adverse event and clinical outcomes for feminizing and masculinizing gGAS.

Scope	Outcome	Outcome assessment method	Required reporting	
Vaginoplasty with a vaginal canal	Loss of neovaginal tissue lining	Physical examination using a speculum	**Complete loss of lining characteristics:** • Time of diagnosis reported as number of weeks (up to 12 weeks) or months (starting from 3 months up) post-primary surgery	**Classification:** Graded according to the Clavien-Dindo Classification.
			**Partial loss of lining characteristics:** • Proportion of canal lining length lost, as measured: ○ <50% loss ○ ≥50% loss • Time of diagnosis reported as number of weeks (up to 12 weeks) or months (starting from 3 months up) post-primary surgery	
	Neo-vaginal stenosis	Clinical history for stenosis-related symptoms	**Characteristics:** Nature of symptoms (open)	**Classification:** Graded according to the Clavien-Dindo Classification.
		Physical examination using a speculum AND dilator insertion	**Characteristics:** • Location: vault (apex) or canal (except introitus) • Extension of stenosis: ○ <50% loss ○ ≥50% loss • Time of diagnosis reported as number of weeks (up to 12 weeks) or months (starting from 3 months up) post-primary surgery	
	Stricture of the neovaginal introitus	Physical examination	**Characteristics:** • Time of diagnosis reported as number of weeks (up to 12 weeks) or months (starting from 3 months up) post-primary surgery	**Classification:** Graded according to the Clavien-Dindo Classification.
	Rectovaginal fistula	Physical examination	**Characteristics:** • Location(s): in the neovaginal canal as distance in cm from the vaginal opening • Time of diagnosis reported as number of weeks (up to 12 weeks) or months (starting from 3 months up) post-primary surgery	**Classification:** Graded according to the Clavien-Dindo Classification.
		IF INCONCLUSIVE; Additional imaging		
Phalloplasty with or without urethral lengthening	Flap necrosis of the neo-phallus	Physical examination of final level of necrosis	**Complete flap necrosis characteristics:** • Specification following primary or secondary surgery Time of diagnosis reported as number of days (up to 14 days) or weeks (starting from 2 to 12 weeks) or months (starting from 3 months up) post-surgery	**Classification:** Graded according to the Clavien-Dindo Classification.
			**Partial flap necrosis characteristics:** • Location: proximal or distal • Depth: superficial or full-thickness • Proportion: calculated percentage of skin flap using a ruler in cm’s • Specification following primary or secondary surgery Time of diagnosis reported as number of days (up to 14 days) or weeks (starting from 2 to 12 weeks) or months (starting from 3 months up) post-surgery	
Masculinizing gGAS with urethral lengthening.	Neo-urethral fistula	Physical examination	**Characteristics:** • Number of fistula’s • Location(s): native, proximal anastomosis, pars fixa, distal anastomosis, and/or phallic part of neo-urethra • Time of diagnosis reported as number of weeks (up to 12 weeks) or months (starting from 3 months up) post-primary surgery	**Classification:** Graded according to the Clavien-Dindo Classification.
		AND OPTIONAL Urethrogram (retrograde urethrography or voiding cystourethrography)		
	Neo-urethral stricture	Uroflowmetry	**Characteristics:** • Q max (mL/sec) • Residual volume (mL)	**Classification:** Graded according to the Clavien-Dindo Classification.
		AND Ultrasound post-void residual volume		
		AND Urethrogram (retrograde urethrography or voiding cystourethrography)	**Characteristics:** • Number of strictures • Location(s): native, proximal anastomosis, pars fixa, distal anastomosis, and/or phallic part of neo-urethra • Length(s) (cm) • Time of diagnosis reported as number of weeks (up to 12 weeks) or months (starting from 3 months up) post-primary surgery	
		AND OPTIONAL:Urethroscopy		
Feminizing and masculinizing gGAS	Additional surgery	Clinical documentation	**Characteristics:** • Primary reason for additional surgery: a) Patient initiated (dissatisfaction/unmet expectations) i. Functional ii. Aesthetic iii. Combination functional & aesthetic b) Healthcare provider initiated (adverse event/medically necessary) • Type of additional surgery • If multiple, sequential numbering Time of additional surgery reported as number of days (up to 14 days) or weeks (starting from 2 to 12 weeks) or months (starting from 3 months up) post-primary surgery	

## Discussion

The second phase of the GenderCOS project focused on identifying and selecting the most appropriate OMIs and additional assessment details for the 20 unique core outcomes included in the two COS for gGAS.This represents the first standardized framework of core outcomes for gGAS, extending beyond defining *what* to measure to also include *how* and *when* core outcomes should be assessed. Consensus was reached on appropriate OMIs, time points, and supplemental information for 19 of the 20 unique core outcomes across the full scope of gGAS. These recommendations represent the minimum essential reporting required to evaluate surgical procedures and should be used in all gGAS research applicable to the scopes of the two COS. The establishment of both conceptual—in phase 1—and operational—in phase 2—components of the COS provides a unified framework to facilitate standardized outcome measurement, allowing comparability across studies, pooling of data, and evidence synthesis. For one outcome in the feminizing COS, satisfaction with neogenital sexual function, no suitable validated measure was available to assess the outcome across the full fgGAS scope. The final two COS integrate both patient-reported and clinical outcomes, reflecting a balanced perspective that captures both surgical results and patient experience. Selection of PROMs for inclusion and recommendation in the COS prioritized instruments validated for the TGD population.

Historically, outcome reporting in gGAS has been highly heterogeneous. The literature contains over 2000 individual outcomes, but for less than half of these, a specific OMI was reported.^3^^,^^4^ Most of these studies relied on non-validated or ad hoc questionnaires, limiting reliability, comparability, and interpretability of findings. Moreover, outcome measurement in gGAS has primarily emphasized clinical or surgical outcomes, with PRO assessment historically lacking consistency and standardization. The two COS directly address this gap by incorporating a comprehensive set of PROs and corresponding OMIs. This reflects an ongoing transition in gGAS toward patient-centered outcome reporting, as exemplified by the GENDER-Q initiative.^12^ Its close alignment with the core outcomes underscores the complementary nature of these tools and provides an unprecedented opportunity to consolidate outcome assessment across all dimensions of gGAS.

Compared to other COS development studies, the present work progresses beyond the conventional determination of core outcomes by also defining how and when to measure them.^21^ Our study achieved consensus on OMIs for 19 of the 20 unique core outcomes across the full scope of gGAS. This can largely be attributed to the GENDER-Q, which now provides validated measures for most core outcomes in the two COS and helps bridge the previous gap of PROM underrepresentation in gGAS research.

Although this study followed the methodological recommendations from COSMIN and COMET, certain unique considerations apply to the present work. The approach was intentionally pragmatic and tailored to unique considerations in gGAS research. Our search strategy was designed to identify all potentially relevant OMIs, irrespective of validation status, recognizing that suitable tools might exist outside the recommended search framework. We selected and excluded instruments that did not capture the targeted core outcomes, focusing only on conceptually relevant tools for quality appraisal. Although the COMET framework recommends reaching consensus on definitions as part of phase 2, we deemed essential to ensure conceptual agreement on core outcomes and definitions in Phase 1 before instrument selection. The use of a structured online survey as the primary consensus mechanism proved pragmatic and effective, allowing broad international participation. High levels of agreement were achieved through the survey, and only a small number of items required further refinement during the subsequent SSG meeting. This approach enabled balanced expert input and ensured feasibility, while maintaining a high level of participation and methodological rigor.

The GenderCOS now provides an immediately implementable package of core outcomes and respective assessment modalities. The next steps should focus on the integration of the recommended OMIs into registries, clinical trials, and guidelines, including consideration of COS use within journal author guidelines and by research ethics committees during protocol evaluation. The SPIRIT guidance for clinical trial protocols encourages investigators to determine whether a relevant COS exists for their study and to include those core outcomes within their trial design.^22^ Similarly, the IDEAL Framework guidelines for improving the quality of surgical studies recommend the use of standard, well-defined measures for reporting surgical outcomes.^23^ Importantly, the presence of a COS does not limit the inclusion of additional outcomes that may be pertinent to a specific study. Adherence to the recommended instruments and additional requirements is crucial to ensure consistency, comparability, reproducibility, and evidence synthesis. For these reasons, ready-to-use data collection forms and guidance documents have been developed to facilitate adoption across diverse research and clinical contexts. These forms are available in the Supplementary Material or online at https://www.gendercos.org. Additional efforts should focus on training clinicians and research staff, integrating COS items into electronic health records and case report forms, and ensuring clear instructions for timing and scoring of each measure. Importantly, the selection of either masculinizing or feminizing COS should be determined by the surgical intervention(s) performed rather than gender identity. This allows application of the COS to nonbinary individuals and to patients undergoing partial or procedure-specific interventions. While acknowledging the conceptual limitations of this gender binary-anchored classification, it provides a pragmatic framework for grouping procedures with comparable surgical intent. Implementation challenges include limited visibility of the COS, variability in clinical pathways, data collection capacity across centers, and the need for ongoing updates as new instruments are developed.^24^ Notably, the requirement for at least 12-month postoperative follow-up may be difficult to achieve in routine practice due to attrition, resource constraints, and patient compliance. Sustained engagement from stakeholders will be critical to successful and sustained adoption.

This study presents several strengths. First, it aligns with methodological standards set by the COMET Initiative, offering clear recommendations on how to assess core outcomes and thereby facilitating COS uptake and implementation. Second, the systematic search strategy, drawing from both previous systematic reviews and primary database search, ensured that all previously reported OMIs were retrieved and analyzed. A recent systematic review confirmed our findings and identified no additional PROMs beyond those included in our search.^25^ Third, the participation of clinical experts across multiple specialties and various centers worldwide in the consensus survey supports the generalizability of the findings. Lastly, the relatively simple and harmonized structure of assessment tools and timing across both the feminizing and masculinizing COS domains further supports easier COS implementation in clinical and research settings. OMIs were evaluated based on measurement quality, feasibility, clinical relevance, and compatibility with real-world implementation in clinical practice, striving to combine diagnostic accuracy with maximal uptake of the COS. This ensured that only parameters essential for research and data synthesis were collected, minimizing the burden of data collection.

Some limitations must be acknowledged. First, methodological adaptations from COMET recommendations were made to support feasibility and stakeholder participation. Although COSMIN-COMET development frameworks provide some guidance, there is no universally established standard for determining consensus on OMIs. Moreover, no formal protocol was published for this phase. We strived to ensure transparency by clearly reporting the procedures used and by applying the same consensus threshold as in Phase 1. The use of an electronic survey and English as the sole language of participation may have introduced selection bias. Geographic representation of the PEs was limited, with participants primarily based in Europe and the Americas. This may affect the generalizability of the recommendations to settings with different care pathways and resource availability. The employed 3-point Likert response scale, while intentionally pragmatic, could have inflated apparent agreement by restricting the range of dissent. Because the consensus threshold combined “agree” and “mostly agree,” variation in respondents’ confidence may be less visible. Full response distributions are therefore reported (Table 4), and qualitative feedback informed refinements during the SSG meeting. For this second phase, we chose to involve clinical experts only. The contribution of lived experience experts (LEEs) was fundamental during the first phase of the project, ensuring that selected outcomes reflect what matters most to TGD individuals. It was decided not to consult LEE in Phase 2 because of the clinical focus and the highly technical nature of selecting outcome measurement instruments. However, this decision may have affected the prioritization of OMIs. To reduce this risk and ensure continued population involvement and relevance, only TGD -validated PROMs were selected. For some core outcomes, no validated instruments currently exist. While this is a common challenge in COS development studies,^21^ it limited our ability to recommend established tools and may affect the uptake of the COS until suitable instruments become available. The use of question items and data that were previously field-tested in the GENDER-Q project partially fills this gap and provides validated instruments for some of these core outcomes. Although these items were selected to measure the same constructs that had achieved PE consensus and were developed within the same qualitative framework as the published GENDER-Q scales, they were not subjected to a second formal consensus vote. Item-level fit statistics and differential item functioning results for these specific item sets are not publicly available at the time of submission; however, psychometric summaries provided by the developers indicated acceptable item performance and reliability within the field-test dataset. No formal confirmatory expert review of these substituted instruments is currently planned. However, the included instruments may be reassessed during future update cycles as new evidence and implementation data emerge. Lastly, although the GENDER-Q scales are validated and robust, real-world performance data are still limited. Further validation studies are warranted to ensure optimal psychometric performance across diverse populations. Furthermore, information about the interpretability of the scores is needed.

The present COS should be regarded as a dynamic framework that will evolve alongside emerging evidence and real-world data. To ensure broader applicability, future work should focus on developing and validating instruments for outcomes currently lacking suitable assessment tools, particularly for satisfaction with neogenital sexual function in feminizing gGAS. The evaluation of implementation and uptake of the COS and its instruments across diverse care settings represents an additional essential step. The GenderCOS provides the first comprehensive and consensus-based framework of outcome measurement instruments for the two core outcome sets for feminizing and masculinizing gGAS. By defining not only the core outcome (*what* to measure) but also the appropriate instruments and timing (*how* and *when* to measure), these recommendations provide the foundation for standardized assessment and reporting. Adoption of the COS and adherence to the recommended assessment modalities will improve comparability across studies, enhance evidence synthesis and ensure that outcomes most meaningful to TGD individuals remain at the center of research and clinical evaluation.

## Contributors

Conceptualization: all authors contributed equally. Data curation: PJR, MA. Formal analysis: PJR, MA, MGM. Methodology: PJR, MA, MSV, MGM. Project administration: PJR, MA. Resources: all authors contributed equally. Software: PJR. Supervision: MGM. Writing–original draft: MA, PJR, MGM. Writing–review & editing: all authors contributed equally. PJR and MA accessed and verified the underlying data.

## Data sharing statement

The data supporting this study's findings are primarily available in the Supplementary material. Any additional data are available upon reasonable request from the corresponding author.

## Declaration of interests

Author WPB has served as a paid consultant for Karo Healthcare (Stockholm, Sweden), as an unpaid President of the WPATH (past), and as a paid Editor-in-Chief of the *International Journal of Transgender Health* (past). All other authors declare no conflicts of interest.
