# Supplementary material for: Outcome measurement instruments for the core outcome sets on genital gender-affirming surgery: the GenderCOS project
Source: eClinicalMedicine. 2026 Apr 18;95:103907. doi: 10.1016/j.eclinm.2026.103907 (PMC13098459; doi:10.1016/j.eclinm.2026.103907)
Supplement: Supplementary File S1 [file mmc1.docx]

**Checklist for Core Outcomes for Feminizing Genital Gender-affirming Surgery.**

Use at post-operative visits within the first 12 months.

**SECTION 1 – CLINICAL OUTCOMES**

**Additional Surgery**

- **Was any additional surgery performed?**
  ☐ Yes ☐ No
- **Reason for additional surgery:**
    ☐ Patient-initiated
     ☐ Functional ☐ Aesthetic ☐ Both
    ☐ Provider-initiated (adverse event/medically necessary)
- **Type of additional surgery:** ___________________________________________________________________
- **Date of additional surgery:** ___ / ___ / ______
- **Time since primary surgery:**
  - ☐ ___ Days (up to 14 days)
  - ☐ ___ Weeks (from 2 to 12 weeks)
  - ☐ ___ Months (from 3 months up)
- **Sequential number (if multiple):** ______ (fill in one form for each additional surgery)

**SECTION 2 – ADVERSE EVENTS**

**Loss of Neovaginal Tissue Lining**

- **Outcome assessment method:**
  - Physical examination with speculum
    - ☐ Complete loss of lining
    - ☐ Partial loss of lining
- **Characteristics:**
  - If partial loss of lining:
    - ☐ <50% of canal lining loss
    - ☐ ≥50% of canal lining lost
  - Time of diagnosis:
    - ☐ ___ Weeks (up to 12 weeks)
    - ☐ ___ Months (from 3 months up)
- **Classification**:
  - Clavien-Dindo Grade:

| **Grade** | **Definition** |  |
| --- | --- | --- |
| **I** | Any deviation from the normal postoperative course without the need for pharmacological treatment or surgical, endoscopic, and radiological interventions. Acceptable treatments: antiemetics, antipyretics, analgesics, diuretics, electrolytes, physiotherapy. Includes bedside wound opening. | ☐ |
| **II** | Complication requiring pharmacological treatment with drugs other than those allowed for Grade I. Includes blood transfusions and total parenteral nutrition. | ☐ |
| **III** | Complication requiring surgical, endoscopic, or radiological intervention |  |
|  | **IIIa**. Intervention not under general anesthesia | ☐ |
|  | **IIIb**. Intervention under general anesthesia | ☐ |
| **IV** | Life-threatening complication requiring ICU management |  |
|  | **IVa**. Single organ dysfunction | ☐ |
|  | **IVb**. Multi-organ dysfunction | ☐ |
| **V** | Death of the patient | ☐ |

**Neo-vaginal Stenosis**

- **Outcome assessment method:**
  - Clinical history for symptoms
    - Describe symptoms: _______________________
  - Physical examination with speculum and dilator insertion
- **Characteristics:**
  - Location: ☐ Vault (apex) ☐ Canal
  - Extension: ☐ <50% ☐ ≥50%
  - Time of diagnosis:
    - ☐ ___ Weeks (up to 12 weeks)
    - ☐ ___ Months (from 3 months up)
- **Classification**:
  - Clavien-Dindo Grade:

| **Grade** | **Definition** |  |
| --- | --- | --- |
| **I** | Any deviation from the normal postoperative course without the need for pharmacological treatment or surgical, endoscopic, and radiological interventions. Acceptable treatments: antiemetics, antipyretics, analgesics, diuretics, electrolytes, physiotherapy. Includes bedside wound opening. | ☐ |
| **II** | Complication requiring pharmacological treatment with drugs other than those allowed for Grade I. Includes blood transfusions and total parenteral nutrition. | ☐ |
| **III** | Complication requiring surgical, endoscopic, or radiological intervention |  |
|  | **IIIa**. Intervention not under general anesthesia | ☐ |
|  | **IIIb**. Intervention under general anesthesia | ☐ |
| **IV** | Life-threatening complication requiring ICU management |  |
|  | **IVa**. Single organ dysfunction | ☐ |
|  | **IVb**. Multi-organ dysfunction | ☐ |
| **V** | Death of the patient | ☐ |

**Stricture of the neovaginal introitus**

- **Outcome assessment method:**
  - Physical examination
- **Characteristics:**
  - Time of diagnosis:
    - ☐ ___ Weeks (up to 12 weeks)
    - ☐ ___ Months (from 3 months up)
- **Classification**:
  - Clavien-Dindo Grade:

| **Grade** | **Definition** |  |
| --- | --- | --- |
| **I** | Any deviation from the normal postoperative course without the need for pharmacological treatment or surgical, endoscopic, and radiological interventions. Acceptable treatments: antiemetics, antipyretics, analgesics, diuretics, electrolytes, physiotherapy. Includes bedside wound opening. | ☐ |
| **II** | Complication requiring pharmacological treatment with drugs other than those allowed for Grade I. Includes blood transfusions and total parenteral nutrition. | ☐ |
| **III** | Complication requiring surgical, endoscopic, or radiological intervention |  |
|  | **IIIa**. Intervention not under general anesthesia | ☐ |
|  | **IIIb**. Intervention under general anesthesia | ☐ |
| **IV** | Life-threatening complication requiring ICU management |  |
|  | **IVa**. Single organ dysfunction | ☐ |
|  | **IVb**. Multi-organ dysfunction | ☐ |
| **V** | Death of the patient | ☐ |

**Rectovaginal Fistula**

- **Outcome assessment method:**
  - Physical examination
  - IF INCONCLUSIVE: imaging: ☐ Performed ☐ Not performed
    - Imaging modality: _______________________
- **Characteristics:**
  - Location from vaginal opening: ______ cm
  - Time of diagnosis:
    - ☐ ___ Weeks (up to 12 weeks)
    - ☐ ___ Months (from 3 months up)
- **Classification**:
  - Clavien-Dindo Grade:

| **Grade** | **Definition** |  |
| --- | --- | --- |
| **I** | Any deviation from the normal postoperative course without the need for pharmacological treatment or surgical, endoscopic, and radiological interventions. Acceptable treatments: antiemetics, antipyretics, analgesics, diuretics, electrolytes, physiotherapy. Includes bedside wound opening. | ☐ |
| **II** | Complication requiring pharmacological treatment with drugs other than those allowed for Grade I. Includes blood transfusions and total parenteral nutrition. | ☐ |
| **III** | Complication requiring surgical, endoscopic, or radiological intervention |  |
|  | **IIIa**. Intervention not under general anesthesia | ☐ |
|  | **IIIb**. Intervention under general anesthesia | ☐ |
| **IV** | Life-threatening complication requiring ICU management |  |
|  | **IVa**. Single organ dysfunction | ☐ |
|  | **IVb**. Multi-organ dysfunction | ☐ |
| **V** | Death of the patient | ☐ |

**SECTION 3 – PATIENT-REPORTED OUTCOMES**

**Health-related Quality of Life**

- **Outcome assessment method:**
  - PROMIS scale: Global Health
    - Calculated T-score and Standard Error (SE) for scale: ___

| **Please respond to each question or statement by marking one box per row.** | | | | | | | | | | | | |
| --- | --- | --- | --- | --- | --- | --- | --- | --- | --- | --- | --- | --- |
|  | Excellent (5) | | Very good (4) | | Good (3) | | | Fair (2) | | | Poor (1) | |
| In general, would you say your health is: | □ | | □ | | □ | | | □ | | | □ | |
| In general, would you say your quality of life is: | □ | | □ | | □ | | | □ | | | □ | |
| In general, how would you rate your physical health? | □ | | □ | | □ | | | □ | | | □ | |
| In general, how would you rate your mental health, including your mood and your ability to think? | □ | | □ | | □ | | | □ | | | □ | |
| In general, how would you rate your satisfaction with your social activities and relationships? | □ | | □ | | □ | | | □ | | | □ | |
| In general, how would you rate your satisfaction with your social activities and relationships? | □ | | □ | | □ | | | □ | | | □ | |
|  | Completely (5) | | Mostly (4) | | Moderately (3) | | | A little (2) | | | Not at all (1) | |
| To what extent are you able to carry out your everyday physical activities such as walking, climbing stairs, carrying groceries, or moving a chair? | □ | | □ | | □ | | | □ | | | □ | |
| **In the past 7 days…** | | | | | | | | | | | | |
|  | Never (5) | | Rarely (4) | | Sometimes (3) | | | Often (2) | | | Always (1) | |
| How often have you been bothered by emotional problems such as feeling anxious, depressed or irritable? | □ | | □ | | □ | | | □ | | | □ | |
|  | None (1) | | Mild (2) | | Moderate (3) | | | Severe (4) | | | Very severe (5) | |
| How would you rate your fatigue on average? | □ | | □ | | □ | | | □ | | | □ | |
|  | 1 | 2 | 3 | 4 | 5 | 6 | 7 | | 8 | 9 | | 10 |
| How would you rate your pain on average? (1: No pain; 10: Worst pain imaginable) | □ | □ | □ | □ | □ | □ | □ | | □ | □ | | □ |

**Genital gender congruence**

- **Outcome assessment method:**
  - Gender affirmation questions
    - Score for question 1 (labia): ___
    - Score for question 2 (clitoris): ___

**GENDER-Q – GENDER AFFIRMATION QUESTIONS**

|  | EXTREMELY dissatisfied (1) | VERY  Dissatisfied (2) | SOMEWHAT dissatisfied (3) | SOMEWHAT satisfied (4) | VERY  Satisfied (5) | EXTREMELY satisfied (6) |
| --- | --- | --- | --- | --- | --- | --- |
| 1) How much having labia makes you feel affirmed in your gender? | □ | □ | □ | □ | □ | □ |
| 2) How much having a clitoris makes you feel affirmed in your gender? | □ | □ | □ | □ | □ | □ |

*The PROMs marked GENDER-Q are copyright of McMaster University and Brigham and Women’s Hospital (© 2024, McMaster University and Brigham and Women’s Hospital). The GENDER-Q must not be copied, distributed, or used in any way without the prior consent of McMaster University.*

*Use of any GENDER-Q scale, short form, or single item requires permission from the developers. Scoring manuals, licensing information, and the official GenderCOS package, including the outcome-specific forms used in this checklist, are available at:* [*https://www.genderq.org*](https://www.genderq.org)

**Satisfaction with surgical result**

- **Outcome assessment method:**
  - GENDER-Q scale: Treatment outcome
    - Converted Rasch score for scale: ___

**Satisfaction with aesthetic outcome**

- **Outcome assessment method:**
  - GENDER-Q scale: Labia
    - Converted Rasch score for scale: ___
  - GENDER-Q scale: Clitoris
    - Converted Rasch score for scale: ___

**Erogenous sensibility of the genitals**

- **Outcome assessment method:**
  - GENDER-Q scale: Female genital sensation
    - Converted Rasch score for scale: ___

**Satisfaction with neo-genital sexual function**

- **Outcome assessment method:**
  - Only if vaginoplasty with depth: GENDER-Q scale: Vagina
    - Converted Rasch score for scale: ___

**Checklist for Core Outcomes for Masculinizing Genital Gender-affirming Surgery.**

Use at post-operative visits within the first 12 months.

**SECTION 1 – CLINICAL OUTCOMES**

**Additional Surgery**

- **Was any additional surgery performed?**
  ☐ Yes ☐ No
- **Reason for additional surgery:**
    ☐ Patient-initiated
     ☐ Functional ☐ Aesthetic ☐ Both
    ☐ Provider-initiated (adverse event/medically necessary)
- **Type of additional surgery:** ___________________________________________________________________
- **Date of additional surgery:** ___ / ___ / ______
- **Time since primary surgery:**
  - ☐ ___ Days (up to 14 days)
  - ☐ ___ Weeks (from 2 to 12 weeks)
  - ☐ ___ Months (from 3 months up)
- **Sequential number (if multiple):** ______

**SECTION 2 – ADVERSE EVENTS**

**Flap Necrosis of the Neo-phallus**

- **Outcome assessment method:**
  - Physical examination of final level of necrosis
    - ☐ Partial flap necrosis
    - ☐ Complete flap necrosis
- **Characteristics:**
  - If partial:
    - Location: ☐ Proximal ☐ Distal
    - Depth: ☐ Superficial ☐ Full-thickness
    - Proportion of flap necrosed: _______% of skin flap
  - After: ☐ Primary surgery ☐ Secondary surgery
  - Time of diagnosis:
    - ☐ ___ Days (up to 14 days)
    - ☐ ___ Weeks (from 2 to 12 weeks)
    - ☐ ___ Months (from 3 months up)
- **Classification**:
  - Clavien-Dindo Grade:

| **Grade** | **Definition** |  |
| --- | --- | --- |
| **I** | Any deviation from the normal postoperative course without the need for pharmacological treatment or surgical, endoscopic, and radiological interventions. Acceptable treatments: antiemetics, antipyretics, analgesics, diuretics, electrolytes, physiotherapy. Includes bedside wound opening. | ☐ |
| **II** | Complication requiring pharmacological treatment with drugs other than those allowed for Grade I. Includes blood transfusions and total parenteral nutrition. | ☐ |
| **III** | Complication requiring surgical, endoscopic, or radiological intervention |  |
|  | **IIIa**. Intervention not under general anesthesia | ☐ |
|  | **IIIb**. Intervention under general anesthesia | ☐ |
| **IV** | Life-threatening complication requiring ICU management |  |
|  | **IVa**. Single organ dysfunction | ☐ |
|  | **IVb**. Multi-organ dysfunction | ☐ |
| **V** | Death of the patient | ☐ |

**Neo-urethral Fistula**

- **Outcome assessment method:**
  - Physical examination
  - OPTIONAL imaging:
    - Retrograde urethrogram: ☐ Performed ☐ Not performed
    - Voiding cystourethrography (VCUG): ☐ Performed ☐ Not performed
- **Characteristics:**
  - Number of fistulas: ______
  - Locations involved:
    - ☐ Native urethra
    - ☐ Proximal anastomosis
    - ☐ Pars fixa
    - ☐ Distal anastomosis
    - ☐ Phallic urethra
  - Time of diagnosis:
    - ☐ ___ Weeks (up to 12 weeks)
    - ☐ ___ Months (from 3 months up)
- **Classification**:
  - Clavien-Dindo Grade:

| **Grade** | **Definition** |  |
| --- | --- | --- |
| **I** | Any deviation from the normal postoperative course without the need for pharmacological treatment or surgical, endoscopic, and radiological interventions. Acceptable treatments: antiemetics, antipyretics, analgesics, diuretics, electrolytes, physiotherapy. Includes bedside wound opening. | ☐ |
| **II** | Complication requiring pharmacological treatment with drugs other than those allowed for Grade I. Includes blood transfusions and total parenteral nutrition. | ☐ |
| **III** | Complication requiring surgical, endoscopic, or radiological intervention |  |
|  | **IIIa**. Intervention not under general anesthesia | ☐ |
|  | **IIIb**. Intervention under general anesthesia | ☐ |
| **IV** | Life-threatening complication requiring ICU management |  |
|  | **IVa**. Single organ dysfunction | ☐ |
|  | **IVb**. Multi-organ dysfunction | ☐ |
| **V** | Death of the patient | ☐ |

**Neo-urethral Stricture**

- **Outcome assessment method:**
  - Uroflowmetry
  - Ultrasound post-void residual (PVR)
  - Urethrogram (retrograde urethrography or voiding cystourethrography (VCUG))
  - OPTIONAL: Urethroscopy: ☐ Performed ☐ Not performed
- **Characteristics:**
  - Qmax (mL/s): ______
  - Residual volume (mL): ______
  - Number of strictures: ______
  - Location(s):
    - ☐ Native
    - ☐ Proximal anastomosis
    - ☐ Pars fixa
    - ☐ Distal anastomosis
    - ☐ Phallic urethra
  - Length(s) (cm): ______
  - Time of diagnosis:
    - ☐ ___ Weeks (up to 12 weeks)
    - ☐ ___ Months (from 3 months up)
- **Classification**:
  - Clavien-Dindo Grade:

| **Grade** | **Definition** |  |
| --- | --- | --- |
| **I** | Any deviation from the normal postoperative course without the need for pharmacological treatment or surgical, endoscopic, and radiological interventions. Acceptable treatments: antiemetics, antipyretics, analgesics, diuretics, electrolytes, physiotherapy. Includes bedside wound opening. | ☐ |
| **II** | Complication requiring pharmacological treatment with drugs other than those allowed for Grade I. Includes blood transfusions and total parenteral nutrition. | ☐ |
| **III** | Complication requiring surgical, endoscopic, or radiological intervention |  |
|  | **IIIa**. Intervention not under general anesthesia | ☐ |
|  | **IIIb**. Intervention under general anesthesia | ☐ |
| **IV** | Life-threatening complication requiring ICU management |  |
|  | **IVa**. Single organ dysfunction | ☐ |
|  | **IVb**. Multi-organ dysfunction | ☐ |
| **V** | Death of the patient | ☐ |

**SECTION 3 – PATIENT-REPORTED OUTCOMES**

*The PROMs marked GENDER-Q are copyright of McMaster University and Brigham and Women’s Hospital (© 2024, McMaster University and Brigham and Women’s Hospital). The GENDER-Q must not be copied, distributed, or used in any way without the prior consent of McMaster University.*

*Use of any GENDER-Q scale, short form, or single item requires permission from the developers. Scoring manuals, licensing information, and the official GenderCOS package, including the outcome-specific forms used in this checklist, are available at:* [*https://www.genderq.org*](https://www.genderq.org)

**Sensibility in the neo-phallus**

- **Outcome assessment method:**
  - GENDER-Q scale: Penis sensation
    - Converted Rasch score for scale: ___

**Ability to achieve orgasm**

- **Outcome assessment method:**
  - GENDER-Q scale: Orgasm
    - Converted Rasch score for scale: ___

**Sexual well-being**

- **Outcome assessment method:**
  - GENDER-Q scale: Sexual well-being
    - Converted Rasch score for scale: ___

**Satisfaction with neo-genital aesthetic result**

- **Outcome assessment method:**
  - GENDER-Q scale: Penis
    - Converted Rasch score for scale: ___
  - IF APPLICABLE: GENDER-Q scale: Scrotum
    - Converted Rasch score for scale: ___
  - IF APPLICABLE: GENDER-Q scale: Glans
    - Converted Rasch score for scale: ___

**Donor site morbidity**

- **Outcome assessment method:**
  - GENDER-Q scale: Donor site – Adverse effects
    - Converted Rasch score for scale: ___

**Ability to void in a standing position**

- **Outcome assessment method:**
  - GENDER-Q scale: Satisfaction with urinating
    - Converted Rasch score for scale: ___
