# Supplementary material for: Outcome measurement instruments for the core outcome sets on genital gender-affirming surgery: the GenderCOS project
Source: eClinicalMedicine. 2026 Apr 18;95:103907. doi: 10.1016/j.eclinm.2026.103907 (PMC13098459; doi:10.1016/j.eclinm.2026.103907)
Supplement: Supplementary File S2 [file mmc2.docx]

**Table of Contents**

[Table S1. Identified outcome measurement instruments (OMIs) organized by domain. 1](#_Toc225155072)

[Table S2. Relevant guidelines for core outcomes included in the feminizing COS. 13](#_Toc225155073)

[Table S3. Relevant guidelines for core outcomes included in the masculinizing COS. 16](#_Toc225155074)

[Table S4. Quality assessment of identified patient-reported outcome measures (PROMs) for the core outcomes in feminizing genital gender-affirming surgery. 18](#_Toc225155075)

[Table S5. Quality assessment of identified patient-reported outcome measures (PROMs) for the core outcomes in masculinizing genital gender-affirming surgery. 26](#_Toc225155076)

[Table S6. Feasibility of identified patient-reported outcome measures (PROMs) to assess the core outcomes in feminizing genital gender-affirming surgery. 33](#_Toc225155077)

[Table S7. Feasibility of identified patient-reported outcome measures (PROMs) to assess the core outcomes in masculinizing genital gender-affirming surgery. 36](#_Toc225155078)

[Table S8. Feasibility of clinical OMIs for core outcomes applicable to both feminizing and masculinizing genital gender-affirming surgery. 37](#_Toc225155079)

[Table S9. Feasibility of clinical OMIs for core outcomes in feminizing genital gender-affirming surgery. 38](#_Toc225155080)

[Table S10. Feasibility of clinical OMIs for core outcomes in masculinizing genital gender-affirming surgery. 42](#_Toc225155081)

[Supplementary S11. Discussion around tools to assess “Health-related quality of life” in the feminizing COS. 44](#_Toc225155082)

[Supplementary S12. Discussion around tools to assess “Satisfaction with neo-genital sexual function” in the feminizing COS. 45](#_Toc225155083)

[Table S13. Extended results and quotes from feminizing and masculinizing COS consensus survey. 46](#_Toc225155084)

[Supplementary S14. Final consensus survey. 51](#_Toc225155085)

[Supplementary S15. Full search strategy. 68](#_Toc225155086)

# Table S1. Identified outcome measurement instruments (OMIs) organized by domain.

| **Renal and urinary outcome measurements** | |
| --- | --- |
| **OMI** | **Outcome(s)** |
| Amsterdam Hyperactive Pelvic Floor Scale – Women (AHPFS-W) | Urinary tract infection |
|  | Urinary outcomes |
| American Urological Association symptom index (AUA-SI) | Severity of lower urinary tract symptoms and impact on daily life |
| International Consultation on Incontinence Questionnaire – Short Form (ICIQ-SF) | Urinary function |
| International Consultation on Incontinence Questionnaire - Female Lower Urinary Tract Symptoms (ICIQ-FLUTS) | Urinary outcomes |
| International Consultation on Incontinence Questionnaire - Urinary Incontinence (ICIQ-UI) | Urinary outcomes |
| International prostate symptom score (IPSS) | Severity of urinary symptoms and quality of life impact. |
|  | Severity of urinary symptoms and quality of life impact. |
|  | Lower urinary tract symptoms severity |
|  | LUTS |
|  | Urinary function |
| King’s Health Questionnaire | Urinary function |
|  | Urinary incontinence impact on QoL |
|  | Urinary incontinence impact on quality of life |
|  | Urinary urgency and frquency |
|  | Urinary outcomes |
|  | Impact of urinary incontinence on quality of life. |
| Melloni Urinary Tract Questionnaire (ad hoc) | Lower urinary tract symptoms and impact on QoL. |
| No specific instrument provided | Urethral fistula |
| Novel/ad hoc questionnaires | Deviated urinary stream direction or spraying of urinary stream |
|  | Urinary incontinence |
|  | Urinary tract infection |
|  | Ability to void in a standing position |
| Pelvic Floor Distress Inventory (PFDI-20) | Urinary outcomes |
| Postphalloplasty Urinary Function Test (PP UFT) | Urinary function |
| Questionnaires from previously published work | Deviated urinary stream direction or spraying of urinary stream |
|  | Urinary incontinence |
| Sheffield Pelvic Organ Prolapse (SPS-Q) | Urinary urgency |
|  | Urinary outcomes |
| Urinary Distress Inventory (UDI-6) component of the Pelvic Floor Distress Inventory (PFDI-20) questionnaire | Urinary incontinence |
|  | Urinary outcomes |
|  | Urethral integrity and function |
|  | Urinary function |
|  | Neo-urethral fistula |
| Urethral Stricture Surgery Patient-Reported Outcome Measure (USS-PROM) | Urinary function |
| Visual prostate symptom score (VPSS) | Urinary function |
| Clinical assessment | Neo-urethral fistula |
| Clavien-Dindo complications classification system (sufficient for re-operation) | Urethral meatal stenosis |
| Clinical documentation (urinary retention requiring catheter) | Urinary retention |
| Clinical examination (obstructive urinary symptoms) | Urethral meatal stenosis |
| Cystoscope | Neo-urethral stricture |
|  | Bladder and urethra visualization |
| Frequency voiding chart | Urinary frequency and patterns |
| Urinary incontinence requiring antimuscarinic therapy | Urinary incontinence |
| Urinary stream not central or too wide | Deviated urinary stream direction or spraying of urinary stream |
| Uroflowmetry | Urinary flow rates |
|  | Neo-urethral stricture |
|  | Neo-urethral fistula |
| Retrograde urethrocystography | Neo-urethral stricture |
| History and physical exam | Urethral fistula |
| Voiding cystourethrography | Urethral stricture |
|  | Urethral fistula |
| Bladder ultrasound | Urethral fistula |
| MRI | Urethral fistula |
| Ultrasound urethrography | Urethral stricture |
| GENDER-Q – Standing Voiding Short Scale (2 items, field-tested version) | Ability to void in a standing position |
| **Blood and lymphatic system outcome measurements** | |
| **OMI** | **Outcome(s)** |
| Blood loss >500 ml | Bleeding/hemorrhage |
| Clinical documentation (blood units transfused) | Administration of blood transfusion |
|  | Administration of blood transfusion |
| Clinically detected impaired coagulation intra-operatively | Bleeding/hemorrhage |
| Intra-operative hemorrhage requiring transfusion | Bleeding/hemorrhage |
| Requirement for return to theater due to bleeding | Bleeding/hemorrhage |
| Transfusion for anemia (hemoglobin <7g/dl or symptomatic anemia and hemoglobin <8 g/dl) | Administration of blood transfusion |
| **Genitals and breasts outcome measurements** | |
| **OMI** | **Outcome(s)** |
| Amsterdam Hyperactive Pelvic Floor Scale - Women (AHPFS-W) | Hyperactive pelvic floor symptoms. |
|  | Pelvic floor hyperactivity |
|  | Symptoms of hyperactive pelvic floor dysfunction |
|  | Pelvic floor function |
| Colorectal-Anal Distress Inventory-8 (CRADI-8) | Pelvic floor function and ease of vaginal dilation |
| Colorectal-Anal Impact Questionnaire-7 (CRAIQ-7) | Pelvic floor function and ease of vaginal dilation |
| International Continence Society Pelvic Organ Prolapse (ICS-POP) | Symptoms and impact of pelvic organ prolapse on quality of life. |
| New women's gynecological index | N/A |
| Novel/ad hoc questionnaires | Neovaginal stenosis |
|  | Neovaginal depth/length |
|  | Neovaginal width/girth |
|  | Satisfaction with neovaginal depth |
| Patient-reported maximum dilator depth and size | Neovaginal width/girth |
| Patient-reported maximum dilator depth and size with depth assessed from the urethral meatus | Neovaginal depth/length |
| Patient-reported subjective feeling of a narrow introitus | Neovaginal stenosis |
| Pelvic Floor Disorders Inventory (PFDI-20) | Pelvic floor function and ease of vaginal dilation |
| Pelvic Floor Impact Questionnaire (PFIQ-7) | Pelvic floor function and ease of vaginal dilation |
| Post-Affirming Surgery Form and Function Individual Reporting Measure (AFFIRM) | Appearance, urologic (i.e., misdirected urinary stream, nocturia, urinary frequency), and gynecologic (i.e., vaginal pain, satisfaction with vaginal caliber) function |
| Questionnaires from previously published work | Neovaginal stenosis |
|  | Neovaginal depth/length |
|  | Satisfaction with neovaginal depth |
| Sheffield Pelvic Organ Prolapse Quality of Life Questionnaire (SPS-Q) | Vaginal prolapse impact on QoL |
|  | Symptoms and severity of pelvic organ prolapse and impact on daily life. |
|  | Symptoms and severity of pelvic organ prolapse and impact on daily life. |
| Short Questionnaire for Self-evaluation of Vaginoplasty (SQSV) | Self-assessment of satisfaction with appearance and function following vaginoplasty |
| Urinary Distress Inventory (UDI-6) | Pelvic floor function and ease of vaginal dilation |
| PROM ad hoc | Sensibility in the neo-phallus |
| Self-Assessment of Genital Anatomy and Sexual Function (L-SAGASF-F) | Self-perceived satisfaction with genital anatomy and sexual function |
| Patient reported questionnaire including items on vaginal dryness/tightness | Neovaginal stenosis |
| Late Effects of Normal Tissues, Subjective, Objective, Management, Analytic (LENT SOMA), vagina/sexual dysfunction section | Neovaginal stenosis |
| Bouman’s transparent plastic molds | Neovaginal depth/length |
|  | Neovaginal width/girth |
| Clavien-Dindo complications classification system (sufficient for re-operation) | Neovaginal stenosis |
|  | Rectovaginal fistula |
|  | Neovaginal prolapse (partial or total) |
| Clinician assessment using dilators/rod (generic, customized, standardized, Hagar, Young's, size 21 Hegar-Mosquito dilator) | Neovaginal depth/length |
|  | Neovaginal width/girth |
| Digital examination or recorded from patient observation | Neovaginal depth/length |
| MRI | Neovaginal width/girth |
|  | Neovaginal stenosis |
|  | Rectovaginal fistula |
|  | Neovaginal prolapse (partial or total) |
|  | Neovaginal depth/length |
| Neovaginal depth < 10.9 cm or less than two dots on the vaginal dilator | Neovaginal stenosis |
| Neovaginal length < 12 cm or width < 3.5 cm | Neovaginal stenosis |
| Vagina depth <2 cm requiring sigmoid vaginoplasty | Neovaginal stenosis |
| Vagina depth after complete vagina healing | Neovaginal depth/length |
| Application of a metal (cold) or plastic (in comparison warm) element of the TIP THERM device (TIP THERM GmbH, Wischhafen, Lower Saxony, Germany) | Sensibility in the neo-phallus |
| Semmes-Weinstein monofilaments (range 1.65e6.65; Touch Test Sensory Evaluators; North Coast Medical Inc, Morgan Hill, CA) | Sensibility in the neo-phallus |
| Neuropen (OwenMumford Ltd, Woodstock, United Kingdom) with calibrated pressure (40 g) | Sensibility in the neo-phallus |
| Intervals from 1 to 23 mm apart, using the Greulich star (Touch Test e Two-Point Discriminator, North Coast Medical Inc) | Sensibility in the neo-phallus |
| Application of a C64 Hz Rydel-Seiffer tuning fork with a 0e8 scale (vibration, Aesculap AG, Tuttlingen, Hessen, Germany) | Sensibility in the neo-phallus |
| Zachary and Holmes scheme (tactile) | Sensibility in the neo-phallus |
| Regular clinical inspection (early wound complication recognition) | Loss of neovaginal tissue lining |
| Structured follow‑up visits | Loss of neovaginal tissue lining |
| Inspection of neovaginal lining | Loss of neovaginal tissue lining |
| Common Terminology Criteria for Adverse Events (CTCAE v4.0) | Neovaginal stenosis |
|  | Loss of neovaginal tissue lining |
|  | Stricture of neovaginal introitus |
| GENDER-Q — Treatment Outcome Scale | Satisfaction with surgical result |
| GENDER-Q — Vagina/Labia/Clitoris Scales | Satisfaction with aesthetic result |
| **Skin and subcutaneous tissue outcome measurements** | |
| **OMI** | **Outcome(s)** |
| Patient and Observer Scar Assessment Scale (POSAS) | Observer assessment of scar appearance |
|  | Subjective and objective assessment of scar appearance and symptoms |
|  | Patient assessment of scar symptoms and satisfaction |
| Vancouver Scar Scale (VSS) | Objective assessment of scar characteristics, such as pigmentation, height, and pliability |
| Clinical assessment | Complete flap necrosis |
| Clavien-Dindo classification | Partial flap necrosis |
|  | Wound dehiscence |
| Involvement threshold (<10% of flap) | Partial flap necrosis |
| Involvement threshold (size of area cm2) | Partial flap necrosis |
| Histological-staining and examination using a light microscope | Tissue morphology and pathology |
| Flap-survival classification (complete/partial/zero) | Flap necrosis |
| Clinical flap monitoring | Flap necrosis |
| Indocyanine‑green perfusion imaging | Flap necrosis |
| Urgent return for salvage (haematoma evacuation, anastomosis revision) | Flap necrosis |
| Baecke Habitual Physical Activity Questionnaire | Donor-site morbidity |
| Disabilities of the Arm, Shoulder and Hand (DASH) | Donor-site morbidity |
| Quick Disabilities of Arm, Shoulder & Hand (QuickDASH) | Donor-site morbidity |
| SCAR-Q | Donor-site morbidity |
| Patient-Reported Impact of Scars Measure (PRISM) | Donor-site morbidity |
| Bock Scar QoL Questionnaire | Donor-site morbidity |
| Patient Scar Assessment Questionnaire (PSAQ) | Donor-site morbidity |
| GENDER-Q – Donor Site – Adverse effects | Donor-site morbidity |
| **Vascular outcome measurements** |  |
| **OMI** | **Outcome(s)** |
| Clavien-Dindo classification | Hematoma |
| **Physical functioning outcome measurements** |  |
| **OMI** | **Outcome(s)** |
| Baecke physical activity | Physical activity in work, leisure, and daily life |
| Disabilities of Arm, Ahoulder, and Hand (DASH) | Ability of a patient to perform certain upper extremity activities |
| **Sexual functioning outcome measurements** | |
| **OMI** | **Outcome(s)** |
| Arizona Sexual Experience Scale (ASEX) | Sexual functioning in terms of sex drive, arousal, vaginal lubrication/penile erection, ability to reach orgasm, and satisfaction from orgasm |
| Brief Index of Sexual Functioning for Women (BISF-W) | Sexual functioning |
|  | Sexual functioning in women, including arousal, satisfaction, frequency of activity, and overall sexual experiences |
| Brief Sexual Function Inventory (BSFI) | Sexual function |
| Changes in Sexual Functioning Questionnaire (CSFQ) | Changes in sexual functioning across multiple domains, including desire, arousal, orgasm, and sexual behavior |
| Constantino Questionnaire (ad hoc) | Satisfaction with surgical outcomes in 3 domains (sexual parameters and function, satisfaction and happiness with an ongoing relationship, mood, well-being, and aggressive behavior) |
| Derogatis Fantasy Scale (adapted version) | Sexual fantasy frequency and content |
| Erectile Dysfunction Inventory of Treatment Satisfaction (EDITS) | Erectile satisfaction |
|  | Satisfaction with erectile dysfunction treatment. |
| Erection Hardness Scores (EHS) | Erectile satisfaction |
| Female Sexual Distress Scale (FSDS-R) | Distress related to sexual functioning and experiences, particularly in the context of Hypoactive Sexual Desire Disorder |
| Female Sexual Dysfunction Index (FSDI) | Sexual dysfunction across key domains, including desire, arousal, orgasm, pain, and satisfaction |
| Female Sexual Function index (FSFI - original and adapted versions) | Sexual function |
|  | Sexual function across six domains: desire, arousal, lubrication, orgasm, satisfaction, and pain |
|  | Sexual functioning |
|  | Satisfaction with neovaginal depth |
|  | Attainment of orgasm |
|  | Dyspareunia |
|  | Attainment of orgasm |
|  | Sexual function |
| Gay and Lesbian Relationship Satisfaction Scale (GLRSS) | Relationship and sexual satisfaction |
| Golombok-Rust Inventory of Sexual Satisfaction (GRISS) | Sexual satisfaction and problems within relationships, covering aspects like desire, arousal, and frequency of activity. |
| International Index of Erectile Function (IIEF) | Assessment of erectile function |
|  | Sexual function |
| Male Sexual Function Index (MSFI) | Sexual function |
| Male Sexual Function Questionnaire (MSF-4) | Sexual function |
| Male Sexual Health Questionnaire (MSHQ) | Sexual function |
| Multidimensional Sexual Self-Concept Questionnaire (MSSCQ) | Sexual function |
| Multidimensional Sexuality Questionnaire (MSQ) | Sexual function |
| New Sexual Satisfaction Scale (NSSS) | Sexual satisfaction across two dimensions: personal satisfaction (individual experiences) and relational satisfaction |
| Novel/ad hoc questionnaires | Attainment of orgasm |
|  | Dyspareunia |
| Operated Male-to-Female Sexual Function Index (oMtFSFI) | Sexual function |
|  | Assessment of sexual function in male-to-female patients after surgery. |
| PROMIS Sexual Function and Satisfaction v2.0 (PROMIS) | Sexual function |
|  | Sexual function and satisfaction across various domains, including sexual desire, arousal, orgasm, and overall satisfaction |
|  | Sexual function |
| Quality of Sexual Experiences Scale (QSE) | Sexual function |
| Questionnaires from previously published work | Attainment of orgasm |
|  | Dyspareunia |
| Self-Esteem And Relationship questionnaire (SEAR) | Self-esteem and relationship satisfaction, particularly in men with erectile dysfunction |
|  | Sexual function |
| Sexual Arousal and Desire Inventory (SADI) | Levels of sexual arousal and desire, distinguishing between spontaneous and responsive desire |
| Sexual Desire Inventory (SDI) | Sexual desire in partnered and solitary contexts |
|  | Sexual desire |
|  | Sexual function |
| Sexual Function Questionnaire (SFQ-V1) | Comprehensive assessment of female sexual function across multiple domains: arousal, orgasm, pain, and emotional connection |
| Sexual Functioning Index-Gender Spectrum (SFI-GS) | Sexual function |
| Sexual Health Inventory for Men (SHIM) | Sexual function |
| Sexual Life Quality Questionnaire (SLQQ) | Impact of sexual health and functioning on quality of life (in patients with ED) |
| Sexual Satisfaction Scale for Women (SSS-W) | Sexual satisfaction and sexual distress across five domains (contentment, communication, compatibility, relational concern, and personal concern) |
| Short Questionnaire for Self-evaluation of Vaginoplasty (SQSV) | Attainment of orgasm |
| Symptom Checklist-90 (SCL 90) | Sexual function |
| Wierckx Questionnaire: sexual functioning | Sexual functioning, including desire and satisfaction. |
|  | Quality of life and sexual health after sex reassignment surgery in transsexual men |
| Ad hoc questionnaire | Ability to achieve orgasm |
|  | Sexual wellbeing |
|  | Ability to perform penetrative sexual intercourse |
| Modified hypospadias questionnaire | Sexual wellbeing |
| Sexual Satisfaction and Function (SatisFunction) survey | Satisfaction with neogenital sexual function |
| Patient-Reported Outcomes in Genital Reconstructive Surgeries (PROGRESS) | Satisfaction with neogenital sexual function |
| GENDER-Q — Sexual Well-Being Scale | Satisfaction with neogenital sexual function |
| GENDER-Q — Vagina Scale | Satisfaction with neogenital sexual function |
| Patient-Reported Outcomes in Genital Reconstructive Surgeries (PROGRESS) | Erogenous sensibility of the genitals |
| GENDER-Q — Sexual Well-Being Scale | Erogenous sensibility of the genitals |
| GENDER-Q – Female Genital Sensation Scale (5 items) | Erogenous sensibility of the genitals |
| Self-Assessment of Genital Anatomy & Sexual Function – Male (SAGASF-M) | Sensibility in the neo-phallus |
|  | Sexual well-being |
| GENDER-Q – Penis Sensation Scale | Sensibility in the neo-phallus |
| GENDER-Q – Orgasm Scale | Ability to achieve orgasm |
| GENDER-Q – Sexual Well-Being Scale | Sexual well-being |
| **Social and Role functioning outcome measurements** | |
| **OMI** | **Outcome(s)** |
| Freiburg Personality Inventory (FPI) | Personality traits, such as sociability, anxiety, and aggression |
|  | Emotional reactivity and personality traits |
| Gender Minority Stress and Resilience Scale (GMRS) | Gender-related discrimination and stress |
| Inventory of Interpersonal Problems (IIP) | Difficulties in interpersonal relationships |
| Social Role Performance schedule (SRP) | Ability to fulfill social roles and responsibilities |
| Bem Sex Role Inventory (BSRI) | Gender roles and identity, focusing on masculinity, femininity, and androgyny |
| Smith Job Satisfaction Scale/Job Descriptive Index (JDI) | Satisfaction with work performance and job roles |
| **Emotional functioning/wellbeing outcome measurements** | |
| **OMI** | **Outcome(s)** |
| Body Cathexis Scale (BCS) | Body satisfaction |
| Ryff’s Scale of Psychological Well-Being (SPWB) | Psychological well-being across self-acceptance, autonomy, and personal growth |
| Affect Balance Scale (ABS) | Positive and negative affect balance |
| Appearance Schemas Inventory (ASI) | Importance of appearance in self-worth |
|  | Body image |
| Body Congruency Score (BCS) | Body congruence |
| Beck Anxiety Inventory (BAI) | Severity of anxiety symptoms |
| Beck Depression Inventory (BDI) | Severity of depressive symptoms |
|  | Depressive symptom severity (including mood, sleep, and cognitive changes) |
|  | Severity of depressive symptoms |
| Biological Questionnaire for Transvestites and Transsexuals (BQTT) | Psychosocial outcomes |
| Body Image Quality of Life Inventory (BIQLI) | Impact of body image on emotional and social life |
|  | Body image |
| Body Image Scale (BIS) | Body image |
|  | Satisfaction with anatomic parts |
|  | Comfort and confidence in physical appearance in transexual people |
| Body Image Scale (BIS) for transsexuals questionnaire | Body image satisfaction specific to transgender individuals |
|  | Perception and satisfaction with body image |
|  | Body image |
| Body Uneasiness Test (BUT) | Body image dissatisfaction and related issues |
| Body Cathexis Scale (BCS) | Satisfaction with specific body parts |
| BREAST-Q: Augmentation module | Satisfaction with breast augmentation outcomes |
| Brief Symptom Inventory 18 (BSI-18) | Psychological symptoms inventory |
| Biografische Vragenlijst voor Transseksuelen (BVT, biographical questionnaire for transsexuals and transvestites) | Demographic and psychosocial information in transgender individuals |
| Center for Epidemiological Studies-Depression scale (CES-D) | Depressive symptoms in general populations |
|  | Depression and sexually risky behavior |
| Crown Crisp Experiential Index (CCEI)/ Middlesex Hospital Questionnaire (MHQ) | Neurotic symptoms: anxiety, obsessionality, phobia |
| Drug Abuse Screening Test (DAST-10) | Drug abuse |
| Defensive Style Questionnaire (DSQ) | Coping and defense mechanisms under stress |
|  | Psychometric |
| Dissociative Disorders Interview Schedule (DDIS) | Symptoms of dissociative disorders |
| Dissociative Experiences Scale (DES) | Frequency of dissociative experiences |
| Effort-Reward imbalance (ERI) | Work-related effort and reward balance |
| Experiences of Transphobia Scale (ETS) | Stigma |
| Feelings of Inadequacy Scale/ Janis-Field-Eagly Self-esteem measure | Self-esteem and adequacy |
| Female Genital Self Image Score (FGSIS) | Genital self-image |
|  | Perceptions and feelings about one's genitalia (including appearance and function) |
|  | Body image |
| Gender Dysphoria Questionnaire for Adults and Adolescents (GIDYQ-AA) | Gender dysphoria / gender identity |
| Gender Identity Reflection and Rumination Scale (GRRS) | Gender identity reflection and rumination |
| General Health Questionnaire | Psychological distress and mental health |
| Gender Identity Stigma scale (GIS) | Stigma |
| Global Response Assessment (GRA) | Overall patient satisfaction and symptom improvement post-treatment |
| Hospital Anxiety and Depression scale (HAD) | Anxiety and depression levels |
| Hamilton Depression rating scale (HAM-D) | Depression |
| Internal-External Locus of Control (IELoC) | Psychosocial outcomes |
| Kessler Psychological Distress Scale (K10) | Oppression |
| Kinsey Heterosexual/Homosexual rating scale | Spectrum of sexual orientation |
| Multiple Affect Adjective Checklist (MAAC) | Psychosocial outcomes |
| Morrisson Questionnaire (Ad hoc) | Satisfaction with rectosigmoid neocolporrhaphy across different domains (appearance, sexual function, postoperative recovery, and overall satisfaction) |
|  | Body image, sexual satisfaction, and quality of life post-surgery |
| Mooney Problem Checklist (MPC) | Psychosocial outcomes |
| Multiphasic Personality Inventory (MPI) | Psychosocial outcomes |
| Multidimensional Body-Self Relations Questionnaire (MBSRQ) | Body image and appearance orientation. |
|  | Body image |
| Patient Health Questionnaire-4 (PHQ-4) | General mental health, focusing on anxiety and depression symptoms |
|  | Generalized anxiety and depression symptoms |
| Perceived social support questionnaire (F-SozU) | Perceived social support |
| Psychological Gender Affirmation scale (PGA) | Comfort and satisfaction with gender affirmation |
| Patient Health Questionnaire-9 (PHQ-9) | Depression Psychosocial health |
|  | Depression Psychosocial health |
| Questions on Life Satisfaction Modules (FLZ M) | Satisfaction with specific areas of life, such as health, finances, and relationships |
|  | Satisfaction with different aspects of life |
| Rosenberg Self-Esteem Scale (RSES) | Global self-esteem and self-worth |
|  | Self-esteem |
|  | Global self-esteem and self-worth |
|  | Self-esteem and self-worth evaluation |
|  | Self-esteem |
| Sammons Body Image and Sexual Pleasure Questionnaire | Body image satisfaction and sexual pleasure |
| Strong Campbell Interest Inventory (SCII) | Psychometric |
| Sentence Completion (Miale-Holsopple) (SCMH) | Psychometric |
| Sense Of Coherence-13 (SOC-13) | Sense of coherence |
| Situational Inventory of Body Image Dysphoria (SIBID) | Body image |
|  | Body image distress in different situations |
| Clinical Anger Scale (CAS) | Frequency and intensity of anger |
| Spielberger's Trait Anger | Dispositional anger in various situations |
|  | Long-term tendencies toward anxious feelings |
| Short Questionnaire for Self-Evaluation of Vaginoplasty (SQSV) | Aesthetic results and satisfaction |
|  | Aesthetic results and satisfaction |
| Self-Rating Scale – Feelings (SRS-F) | Psychosocial outcomes |
| Strength of Transgender Identity Scale (STIS) | Strength of gender identity |
| Subjective Happiness Scale (SHS) | Overall subjective happiness |
|  | Quality of life |
|  | Subjective happiness and emotional well-being |
|  | Overall subjective happiness and perception of happiness compared to others |
| Symptom Checklist-27 (SCL-27) | Psychological distress |
| Symptom Checklist-90 (SCL-90) | Psychological distress and psychiatric symptoms |
| Tennessee Self-Concept Scale (TSCS) | Self-perception across personal, social, and academic domains |
| Transgender Adaptation and Integration Measure (TG AIM) | Stress and adaptation related to transgender and gender nonconforming (TGNC) individuals |
| Transgender Community Belongingness (TCB) | Community connectedness |
| Transgender Congruence Scale (TCS) | Gender congruence |
|  | Gender identity congruence |
| Transgender Positive Identity Measure (T-PIM) | Positive aspects of TGNC identity |
| Utrecht Gender Dysphoria Scale - Gender Spectrum (UGDS-GS) | Intensity of gender dysphoria and its effects |
|  | Gender dysphoria |
| Vanderbilt Mini Patient-Reported Outcome Measure – Gender (VMP-G) | Quality of life, self-concept, satisfaction, and gender dysphoria |
| Wechsler Adult Intelligence Scale (WAIS) | Psychometric |
| Male Genital Self-Image Scale (MGSIS-7) | Satisfaction with aesthetic appearance |
| Patient-Reported Outcomes in Genital Reconstructive Surgeries (PROGRESS) | Genital gender congruence |
| GENDER-Q — Vagina Scale | Genital gender congruence |
| Genital Gender-affirmation items from previous GENDER-Q field-test version | Genital gender congruence |
| **Global quality of life and Perceived health status outcome measurements** | |
| **OMI** | **Outcome(s)** |
| Cantril ladder of life satisfaction | Overall perceived life satisfaction |
|  | Perception of current and future life quality |
|  | Quality of life |
| Essen Transgender quality of Life Inventory (ETLI) | Quality of life in transgender individuals |
| Questions on Life Satisfaction Modules (FLZ M) | Quality of life and life satisfaction |
| Satisfaction With Life Scale (SWLS) | Overall life satisfaction and well-being |
|  | General satisfaction with life |
|  | Quality of life |
|  | Overall cognitive judgment of life satisfaction |
|  | General life satisfaction and subjective well-being |
| World Health Organization Quality of Life-BREF (WHOQOL-BREF) | Brief quality of life assessment across physical, psychological, social, and environmental domains |
| World Health Organization Quality of Life-100 (WHOQOL-100) | Quality of life across physical, psychological, social, and environmental domains |
| Cornell Medical Index (CMI) | Physical and psychological health symptoms |
| Short Form-36 health survey (SF-36) | Quality of life |
|  | Physical and mental health-related quality of life |
|  | General health status across eight domains, including physical and mental health |
| Short Form-12 health survey (SF-12) | Health-related quality of life |
|  | Physical and mental health-related quality of life |
| PROMIS – Global Health | Health-related quality of life |
| GENDER-Q — Health-Related Quality of Life concept | Health-related quality of life |
| **Delivery of care outcome measurements** | |
| **OMI** | **Outcome(s)** |
| No specific instrument provided | Length of hospital stay |
| Novel/ad hoc questionnaires | Regret about undergoing surgery |
| Questionnaires from previously published work | Regret about undergoing surgery |
| Surgical Satisfaction Questionnaire (SSQ-8) | Satisfaction with surgical results |
|  | Satisfaction with surgical outcomes across functional and aesthetic domains |
| Time from induction of anesthesia to final dressing | Duration of surgery |
| Time of incision to incision closure and dressing | Duration of surgery |
| Days from surgery | Duration of follow-up |
| **Need for further intervention and adverse events outcome measurements** | |
| **OMI** | **Outcome(s)** |
| Short Questionnaire for Self-evaluation of Vaginoplasty (SQSV) | Revisional/secondary surgery (no further details specified) |
| No specific instrument provided | Infection (site/severity not further specified) |
| Clinical documentation | Need for re-intervention neo-urethral fistula |
|  | Need for re-intervention neo-urethral stricture |
| Clavien-Dindo classification | Surgical complications |
| **Gastrointestinal outcome measurements** | |
| **OMI** | **Outcome(s)** |
| Colorectal-anal distress Inventory 8 (CRADI-8) component of the Pelvic floor distress Inventory (PFDI-20) questionnaire | Colorectal-anal distress and functional symptoms |
| Clavien-Dindo complications classification system (sufficient for re-operation) | Rectal injury |
| Intra-operative detection and closure (with/without colostomy) | Rectal injury |
| Clinical history and examination | Rectovaginal fistula |
| MRI or contrast fistulography | Rectovaginal fistula |
| Endoscopy | Rectovaginal fistula |
| MRI pelvis without and with IV contrast | Rectovaginal fistula |
| CT pelvis with IV contrast | Rectovaginal fistula |
| Common Terminology Criteria for Adverse Events (CTCAE v4.0) – Vaginal fistula | Rectovaginal fistula |
| **General outcomes** | |
| **OMI** | **Outcome(s)** |
| Brief Pain Inventory Short Form (BPI-SF) | Pain |
| (Modified) Mackinnon Pain Questionnaire | Pain levels and impact on daily functioning |
|  | Pain |

# Table S2. Relevant guidelines for core outcomes included in the feminizing COS.

| **Outcome** | **Guideline** | **Recommended measurement instruments or modalities** | **Source** | **Notes** | **Relevance to Core Outcome** |
| --- | --- | --- | --- | --- | --- |
| Satisfaction with neo‑genital sexual function | European Society for Sexual Medicine (ESSM) position statement on sexual wellbeing after gender‑affirming surgery (2022) | Patient‑reported sexual‑function questionnaires | [ESSM Position Statement "Sexual Wellbeing After Gender Affirming Surgery"](https://pubmed.ncbi.nlm.nih.gov/34971864/) | ESSM stresses using validated sexual‑function scales and adapting them for transgender patients; it does not prescribe a single instrument. | Low |
| Erogenous sensibility of the genitals | European Society for Sexual Medicine (ESSM) position statement on sexual wellbeing after gender affirming surgery (2022) | Patient‑reported assessment of genital sensation / arousal | [ESSM Position Statement "Sexual Wellbeing After Gender Affirming Surgery"](https://pubmed.ncbi.nlm.nih.gov/34971864/) | The statement notes the importance of evaluating clitoral and vulvar sensitivity but reports no standardized neuro‑sensory test for routine care. | Low |
| Loss of neovaginal tissue lining | Wounds International – International Best Practice Recommendations for Early Identification and Prevention of Surgical Wound Complications (2020) | Regular clinical inspection (early wound‑complication recognition) | [International Best Practice recommendations for the early indentification and prevention of Surgical Wound Complications – Wounds International](https://woundsinternational.com/best-practice-statements/international-best-practice-recommendations-early-indentification-and-prevention-surgical-wound-complications/) | The guideline advocates frequent wound assessment and early intervention; it does not provide a validated instrument for quantifying neovaginal tissue loss. | Low |
|  | ACOG Committee Opinion 694 – Management of Mesh and Graft Complications in Gynecologic Surgery (2017) | History and physical examination | [Management of Mesh and Graft Complications in Gynecologic Surgery \| ACOG](https://www.acog.org/clinical/clinical-guidance/committee-opinion/articles/2017/04/management-of-mesh-and-graft-complications-in-gynecologic-surgery) | Recommends thorough clinical evaluation for mesh‑ or graft‑related complications; no measurement tool is specified. | Low |
|  | Common Terminology Criteria for Adverse Events (CTCAE v4.0) – Vaginal perforation | Grading of rupture of the vaginal wall severity (Grades 1–5) | [Common Terminology Criteria for Adverse Events (CTCAE)](https://evs.nci.nih.gov/ftp1/CTCAE/CTCAE_4.03/Archive/CTCAE_4.0_2009-05-29_QuickReference_8.5x11.pdf) | Originally developed for oncology trials; provides anatomic severity grades but is not validated for gGAS. | Moderate |
|  | UCSF Transgender Care – Vaginoplasty guidelines | Structured follow‑up visits | [Vaginoplasty procedures, complications and aftercare \| Gender Affirming Health Program](https://transcare.ucsf.edu/guidelines/vaginoplasty) | The guidelines emphasise clinical inspection of the canal for ischemia/infection and adherence to dilation; measurement is clinician‑reported rather than via a formal instrument. | Moderate |
|  |  | Inspection of neovaginal lining |  |  |  |
|  |  | Teaching of dilation technique |  |  |  |
| Neo‑vaginal stenosis | Common Terminology Criteria for Adverse Events (CTCAE v4.0) – Vaginal obstruction; Vaginal stricture | Grading of vaginal stenosis severity (Grades 1–5) | [Common Terminology Criteria for Adverse Events (CTCAE)](https://evs.nci.nih.gov/ftp1/CTCAE/CTCAE_4.03/Archive/CTCAE_4.0_2009-05-29_QuickReference_8.5x11.pdf) | Originally developed for oncology trials; provides anatomic severity grades but is not validated for gGAS. | Moderate |
|  | EORTC Endometrial Module (EN24) | Patient‑reported questionnaire including items on vaginal dryness/tightness | [Form \| EORTC – Quality of Life](https://qol.eortc.org/form/#3) | Contains items on vaginal symptoms but is designed for endometrial‑cancer patients, not for neovaginal stenosis. | Low |
|  | International Guidelines on Vaginal Dilation after Pelvic Radiotherapy | Common Terminology Criteria for Adverse Events (CTACAE), reproductive (and breast disorders)  section: vaginal stricture, grade 1 to 3 (grade 3 being vaginal narrowing or shortening interfering with  the use of tampons, sexual activity or physical examination) | International Guidelines  on Vaginal Dilation after  Pelvic Radiotherapy | Recommends regular dilation to maintain canal patency. | Moderate |
|  |  | Late Effects of Normal Tissues, Subjective, Objective, Management, Analytic (LENT SOMA), vagina/sexual dysfunction section. |  |  |  |
|  | UCSF Transgender Care – Vaginoplasty guidelines | Assessment of neovaginal depth and width using dilators or digital exam | [Vaginoplasty procedures, complications and aftercare \| Gender Affirming Health Program](https://transcare.ucsf.edu/guidelines/vaginoplasty) | Describes measuring canal dimensions during follow‑up; these measurements are not part of a validated instrument. | Moderate |
|  |  | Patient‑reported maximum dilator size |  |  |  |
| Stricture of the neovaginal introitus | UCSF Transgender Care – Vaginoplasty guidelines | Physical examination | [Vaginoplasty procedures, complications and aftercare \| Gender Affirming Health Program](https://transcare.ucsf.edu/guidelines/vaginoplasty) | Recommends clinician inspection and patient self‑assessment to detect introital narrowing; no validated instrument provided. | Moderate |
|  |  | Measurement with finger or dilator |  |  |  |
|  |  | Patient‑reported ease of dilator passage |  |  |  |
|  | Common Terminology Criteria for Adverse Events (CTCAE v4.0) – Vaginal obstruction; Vaginal stricture | Grading of vaginal stricture severity (Grades 1–5) | [Common Terminology Criteria for Adverse Events (CTCAE)](https://evs.nci.nih.gov/ftp1/CTCAE/CTCAE_4.03/Archive/CTCAE_4.0_2009-05-29_QuickReference_8.5x11.pdf) | Originally developed for oncology trials; provides anatomic severity grades but is not validated for gGAS. | Moderate |
| Rectovaginal fistula | American Society of Colon and Rectal Surgeons (ASCRS) Clinical Practice Guideline for Management of Anorectal Abscess, Fistula‑in‑Ano, and Rectovaginal Fistula (2022) | Clinical history and examination | [The American Society of Colon and Rectal Surgeons Clinical Practice Guidelines for the Management of Anorectal Abscess, Fistula-in-Ano, and Rectovaginal Fistula](https://pubmed.ncbi.nlm.nih.gov/35732009/) | Advises clinical diagnosis followed by imaging (MRI, contrast fistulography) and endoscopic evaluation for complex fistulas; does not specify an outcome‑measurement instrument. | High |
|  |  | MRI or contrast fistulography |  |  |  |
|  |  | Endoscopy |  |  |  |
|  | American College of Radiology (ACR) Appropriateness Criteria on Anorectal Disease (2021) | MRI pelvis without and with IV contrast | [ACR Appropriateness Criteria® Anorectal Disease](https://pubmed.ncbi.nlm.nih.gov/34794588/) | These procedures are equivalent alternatives (ie, only one procedure will be ordered to provide the clinical information to effectively manage the patient’s care). | High |
|  |  | CT pelvis with IV contrast |  |  |  |
|  | Common Terminology Criteria for Adverse Events (CTCAE v4.0) – Vaginal fistula | Grading of vaginal fistula severity (Grades 1–5) | [Common Terminology Criteria for Adverse Events (CTCAE)](https://evs.nci.nih.gov/ftp1/CTCAE/CTCAE_4.03/Archive/CTCAE_4.0_2009-05-29_QuickReference_8.5x11.pdf) | Originally developed for oncology trials; provides anatomic severity grades but is not validated for gGAS. | Moderate |
|  | UCSF Transgender Care – Vaginoplasty guidelines | Clinical assessment of symptoms (feculent discharge, flatus) | [Vaginoplasty procedures, complications and aftercare \| Gender Affirming Health Program](https://transcare.ucsf.edu/guidelines/vaginoplasty) | Suggests diagnosing fistulas based on symptoms and confirmatory imaging; no validated measurement instrument. | Moderate |
|  |  | Imaging (e.g., MRI) when fistula suspected |  |  |  |

gGAS: genital gender-affirming surgery; ACOG: American College of Obstetricians and Gynecologists; UCSF: University of California, San Francisco; EORTC: European Organisation for Research and Treatment of Cancer; MRI: magnetic resonance imaging; CT: computed tomography; IV: intravenous.

# Table S3. Relevant guidelines for core outcomes included in the masculinizing COS.

| **Outcome** | **Guideline** | **Recommended measurement instruments or modalities** | **Source** | **Notes** | **Relevance to Core Outcome** |
| --- | --- | --- | --- | --- | --- |
| Flap necrosis | UK National Flap Registry (UKNFR) | Flap-survival classification (complete/partial/zero) | [https://bahno.org.uk/_userfiles/pages/files/uknfr_first_report_4dec_2019.pdf](https://bahno.org.uk/_userfiles/pages/files/uknfr_first_report_4dec_2019.pdf#:~:text=survival%20as%20complete%20survival%20of,would%20provide) | Registry requires recording unplanned returns to theatre | Low |
|  | ERAS free‑flap management protocols | Clinical flap monitoring | [Enhanced Recovery After Surgery–Based Perioperative Protocol for Head and Neck Free Flap Reconstruction](https://pmc.ncbi.nlm.nih.gov/articles/PMC7268136/) | ERAS protocols highlight flap monitoring and early detection of ischemia; no validated instrument is defined. | Moderate |
|  |  | Optional: indocyanine‑green perfusion imaging |  |  |  |
|  | UCSF Guidelines for the Primary and Gender-Affirming Care of Transgender and Gender Nonbinary People | Flap survival classification | [Phalloplasty and metoidioplasty - overview and postoperative considerations \| Gender Affirming Health Program](https://transcare.ucsf.edu/guidelines/phalloplasty#:~:text=Flap%20loss%20is%20rare%20and,C%20and%20S%20deficiency%2C%20and) | No device specified; some centres use intra‑operative perfusion imaging (e.g., ICG angiography) but not formally endorsed | Moderate |
|  |  | Clinical observation |  |  |  |
|  |  | Urgent return for salvage (haematoma evacuation, anastomosis revision |  |  |  |
| Neo‑urethral fistula | EAU Guidelines on Urethral Stricture Disease | History + physical exam | European Association of Urology. EAU Guidelines on Urethral Strictures (2021).  Eur Urol. 2021 Aug;80(2):190-212. doi:10.1016/j.eururo.2021.02.031 | Comprehensive evaluation for fistulae or strictures | High |
|  |  | Uroflowmetry + PVR |  |  |  |
|  |  | Bladder ultrasound |  |  |  |
|  |  | RUG |  |  |  |
|  |  | VCUG |  |  |  |
|  |  | Cystourethroscopy |  |  |  |
|  | Canadian Urological Associsation Guideline on Male Urethral Stricture | MRI (urethrosymphyseal fistula) | CUA Guideline on Male Urethral Stricture. Can Urol Assoc J. 2020;14(10):E309–E316.  doi: 10.5489/cuaj.6792 | Mentions fistula rates post-urethroplasty (8.4%) and in hypospadias strictures (16–30%) | Moderate |
| Neo‑urethral stricture | EAU Guidelines on Urethral Stricture Disease | Uroflowmetry + PVR | European Association of Urology. EAU Guidelines on Urethral Strictures (2021).  Eur Urol. 2021 Aug;80(2):190-212. doi:10.1016/j.eururo.2021.02.031 | Algorithm for evaluating stricture location and length | High |
|  |  | RUG + VCUG |  |  |  |
|  |  | Cystourethroscopy |  |  |  |
|  |  | Ultrasound urethrography |  |  |  |
|  | AUA Urethral Stricture Disease Guideline Amendment | Uroflowmetry + PVR | Wessells H, Morey A, Vanni AJ, Rahimi L, Souter L, et al. Urethral Stricture Disease Guideline Amendment (2023). J Urol. 2023;210(4):731–744. doi: 10.1097/JU.0000000000003482  . American Urological Association. | Similar to EAU; highlights endoscopic confirmation; RUG/VCUG and cystoscopy are emphasized for diagnosis and staging; flowmetry + PVR used for screening; MRI reserved for complex or associated pathologies (e.g., fistulae, diverticula). | High |
|  |  | Urethro-cystoscopy |  |  |  |
|  |  | RUG |  |  |  |
|  |  | VCUG |  |  |  |
|  |  | Ultrasound urethrography |  |  |  |
|  |  | MRI in selected cases |  |  |  |
|  | CUA Guideline on Male Urethral Stricture | Cystoscopy (first diagnosis) | CUA Guideline on Male Urethral Stricture. Can Urol Assoc J. 2020;14(10):E309–E316. | MRI not routinely recommended; cystoscopy widely available | High |
|  |  | RUG (staging recurrent strictures) |  |  |  |
|  | Dutch Guideline – Richtlijn urethrastricturen | Uroflowmetry | Dutch Association of Urology (Nederlandse Vereniging voor Urologie, NVU). Guideline on Urethral Strictures (Richtlijn Urethrastricturen). June 20, 2017.  Available at: https://radiologen.nl/sites/default/files/secties/abdominale/conceptrichtlijn_urethrastrictuur_20_juni_2017.pdf | RUG is preferred imaging modality; urethroscopy and flowmetry are standard components of diagnostic work-up; advises imaging entire urethra (native & reconstructed) for surgical planning | High |
|  |  | Urethroscopy (flexible) |  |  |  |
|  |  | RUG |  |  |  |
|  |  | VCUG |  |  |  |
|  |  | Urethral ultrasound |  |  |  |

ERAS: Enhanced Recovery After Surgery; UCSF: University of California, San Francisco; ICG: indocyanine green; EAU: European Association of Urology; PVR: post-void residual; RUG: retrograde urethrogram; VCUG: voiding cystourethrogram; MRI: magnetic resonance imaging; CUA: Canadian Urological Association; AUA: American Urological Association; NVU: Nederlandse Vereniging voor Urologie (Dutch Association of Urology).

# Table S4. Quality assessment of identified patient-reported outcome measures (PROMs) for the core outcomes in feminizing genital gender-affirming surgery.

| **Core Outcome** | **Identified PROM** | **Reference** | **Criterion A (Are the relevant aspects of the outcome measured?)** | **Criterion B (Is it validated for the target population?)** | **Criterion C (Is it relevant for use in context of genital gGAS?)** | **Rationale** |
| --- | --- | --- | --- | --- | --- | --- |
| Health-related quality of life | Satisfaction With Life Scale (SWLS) | Diener *et al.* (1985) | No | Yes (general ppl) | Yes | Global life satisfaction; not HRQoL domains; not TGD-validated; sometimes used as QoL proxy post-op |
|  | Questions on Life Satisfaction Modules (FLZ M) | Henrich *et al.* (2000) | No | Yes (general ppl) | Yes | Life-domain satisfaction, not health-focused; no TGD validation; can reflect broad well-being |
|  | 36-Item Short Form Health Survey (SF-36) | Ware *et al.* (1992) | Yes | Yes (general ppl) | Yes | Eight HRQoL domains; robust generic utility; not TGD-validated but commonly applied after surgery |
|  | 12-Item Short Form Health Survey (SF-12) | Ware *et al.* (1996) | Yes | Yes (general ppl) | Yes | PCS/MCS summaries capture HRQoL; efficient; not TGD-validated |
|  | WHO Quality of Life — 100-item (WHOQOL-100) | WHOQOL Group (1998) | Yes | Yes (general ppl) | Yes | Multidomain QoL; broad applicability; no TGD validation |
|  | WHO Quality of Life — Brief (WHOQOL-BREF) | WHOQOL Group (1998) | Yes | Yes (general ppl) | Yes | Brief 4-domain QoL; generic and practical; not TGD-validated |
|  | PROMIS – Global Health | Hays *et al.* (2009) | Yes | Yes (general ppl) | Yes | 10-item generic HRQoL instrument covering physical function, fatigue, pain, emotional distress, social health, and general health/QoL; broadly used across clinical contexts |
|  | Cantril Ladder of Life Satisfaction | Cantril (1965) | No | Yes (general ppl) | No | Single-item life evaluation; lacks HRQoL structure; not TGD-validated; poor specificity for gGAS |
|  | Essen Transgender Quality of Life Inventory (ETLI) | Rücker *et al.* (2025) | Yes | Yes | Yes | TGD-specific HRQoL facets; developed/validated in TGD; directly relevant post-gGAS |
|  | Cornell Medical Index (CMI) | Abramson *et al.* (1965) | No | Yes (general ppl) | No | Symptom checklist rather than HRQoL; no TGD validation; limited relevance to gGAS outcomes |
|  | GENDER-Q — Health-Related Quality of Life concept | Kaur *et al.* (2025) | Yes | Yes | Yes | TGD-developed/validated PRO system; includes HRQoL scales designed for post-GAS assessment |
| Genital gender congruence | Transgender Congruence Scale (TCS) | Kozee *et al.* (2012) | No | Yes | Yes | Measures overall gender congruence (identity/appearance); validated for TGD; not genital-specific but conceptually relevant |
|  | Female Genital Self-Image Scale (FGSIS) | Herbenick *et al.* (2010) | No | No | Yes | Developed for cis women; genital self-image focus; not TGD-validated; appearance relevant post-gGAS |
|  | Post-Affirming Surgery Form and Function Individual Reporting Measure (AFFIRM) | Huber *et al.* (2021) | No | Yes | Yes | Post-gGAS symptom PROM; validated for TGD; congruence not primary but indirectly addressed |
|  | Vanderbilt Mini Patient-Reported Outcome Measure — Gender (VMP-G) | Park *et al.* (2025) | No | Yes | Yes | TGD PROM assessing dysphoria and QoL; not gGAS-focused but relevant to gender congruence |
|  | Body Image Scale (BI-1) | Lindgren *et al.* (1975) | No | Yes | Yes | Assesses satisfaction with body parts; validated for TGD; genital congruence captured indirectly |
|  | Short Questionnaire for Self-Evaluation of Vaginoplasty (SQSV) | Buncamper *et al.* (2015) | No | No | Yes | Post-vaginoplasty evaluation tool; no validation; gGAS-specific |
|  | Self-Assessment of Genital Anatomy and Sexual Function — Feminizing (L-SAGASF-F) | Schober *et al.* (2015) | No | No | Yes | Evaluates anatomy and function of neogenitalia; no formal validation |
|  | Multidimensional Body-Self Relations Questionnaire (MBSRQ) | Cash (1990) | No | No | Yes | Generic body image PROM; includes genital aspects indirectly; no TGD validation |
|  | Sammons Body Image and Sexual Pleasure Questionnaire | Sammons (2010) | No | No | No | Limited psychometric data; not validated; uncertain gGAS relevance |
|  | Sense of Coherence Scale (SOC-13) | Saravia *et al.* (2014) | No | No | No | Psychological resilience measure; unrelated to gender congruence |
|  | Situational Inventory of Body-Image Dysphoria (SIBID / SIBID-S) | Cash (2002) | No | No | Yes | Assesses body-image distress in contexts; includes genital concerns indirectly |
|  | Strength of Transgender Identity Scale (STIS) | Barr *et al.* (2016) | No | Yes | No | Validated for TGD; measures identity strength; not gGAS-related |
|  | Transgender Positive Identity Measure (T-PIM) | Riggle *et al.* (2015) | No | Yes | No | Positive identity facets (authenticity, belonging); TGD validated; not gGAS-focused |
|  | Psychological Gender Affirmation Scale (PGA) | Sevelius *et al.* (2021) | No | Yes | Yes | Measures satisfaction with gender affirmation; validated for TGD; gender congruence addressed conceptually |
|  | Patient-Reported Outcomes in Genital Reconstructive Surgeries (PROGRESS) | *Kanthabalan A. et al.* (2025) | Yes | Yes | Yes | Captures patient perceptions of genital appearance and its impact on daily life; validated in TGD patients undergoing fGRS |
|  | GENDER-Q — Vagina Scale | Kaur *et al.* (2025) | Yes (only one item within the Vagina scale asking about genital gender congruence) | Yes | Yes | TGD-specific and validated; congruence captured in some items, not the main construct |
|  | Genital Gender-affirmation items from previous GENDER-Q field-test version | Kaur *et al.* (2024) | Yes | Yes | Yes | TGD-specific items developed, cognitively tested, and field-tested as part of a previous version of the GENDER-Q; removed from final scales during Rasch-based item reduction for statistical reasons, not for lack of content validity |
| Satisfaction with surgical result | Short Questionnaire for Self-Evaluation of Vaginoplasty (SQSV) | Buncamper *et al.* (2015) | Yes | No | Yes | Developed for postoperative self-assessment; addresses satisfaction and complications; no validation |
|  | Morrison Postoperative Satisfaction Questionnaire | Morrison *et al.* (2015) | Yes | No | Yes | Ad hoc questionnaire; evaluates satisfaction after rectosigmoid vaginoplasty; not validated |
|  | Female Genital Self-Image Scale (FGSIS) | Herbenick *et al.* (2010) | Yes | No | Yes | Measures perception of genital appearance/function; not TGD validated but relevant post-surgery |
|  | Surgical Satisfaction Questionnaire — 8 item (SSQ-8) | Baker (1991) | No | No | No | Generic postoperative satisfaction PROM; not specific to gGAS; not validated for TGD |
|  | Global Response Assessment (GRA) | — | No | No | No | Single global change item; lacks psychometric validation; nonspecific |
|  | Post-Affirming Surgery Form and Function Individual Reporting Measure (AFFIRM) | Huber *et al.* (2021) | Yes | Yes | Yes | TGD-validated postoperative PROM; includes satisfaction and complication domains |
|  | Self-Assessment of Genital Anatomy and Sexual Function — Feminizing (L-SAGASF-F) | Schober *et al.* (2015) | Yes | No | Yes | Evaluates anatomical and functional outcomes post-surgery; no validation but relevant content |
|  | GENDER-Q — Treatment Outcome Scale | Kaur *et al.* (2025) | Yes | Yes | Yes | TGD-specific validated PROM; explicitly measures satisfaction with outcomes after GAS |
| Satisfaction with aesthetic result | Female Genital Self-Image Scale (FGSIS) | Herbenick *et al.* (2010) | Yes | No | Yes | Focuses on perception of genital appearance/function; not TGD validated but relevant post-vaginoplasty |
|  | Morrison Postoperative Satisfaction Questionnaire | Morrison *et al.* (2015) | Yes (only one technique) | No | Yes | Ad hoc measure including appearance items; not validated; rectosigmoid technique-specific |
|  | Short Questionnaire for Self-Evaluation of Vaginoplasty (SQSV) | Buncamper *et al.* (2015) | Yes | No | Yes | Simple post-vaginoplasty PROM addressing aesthetic and functional outcomes; no validation |
|  | Sexual Satisfaction and Function (SatisFunction) survey | Sahmoud *et al.* (2025) | Yes | No | Yes | Domain specifically targets post-surgical genital aesthetics (captures both satisfaction and gender dysphoria/euphoria dimensions); TGD-specific, community-informed tool; only content validity established, full psychometric validation pending |
|  | Post-Affirming Surgery Form and Function Individual Reporting Measure (AFFIRM) | Huber *et al.* (2021) | Yes | Yes | Yes | TGD-validated PROM; includes appearance-related domains relevant to gGAS |
|  | Self-Assessment of Genital Anatomy and Sexual Function — Feminizing (L-SAGASF-F) | Schober *et al.* (2015) | No | No | Yes | Anatomical/functional focus; aesthetic satisfaction not primary; no validation |
|  | GENDER-Q — Vagina/Labia/Clitoris Scales | Kaur *et al.* (2025) | Yes | Yes | Yes | TGD-specific validated PROM suite; includes multiple scales directly assessing aesthetic satisfaction with neogenitalia |
| Satisfaction with neogenital sexual function | Operated Male-to-Female Sexual Function Index (oMtFSFI) | Vedovo *et al.* (2020) | Yes | No (only Italian and French validation) | Yes | Adapted FSFI for transfeminine patients; some validation data; covers multiple sexual domains |
|  | Post-Affirming Surgery Form and Function Individual Reporting Measure (AFFIRM) | Huber *et al.* (2021) | Yes | Yes | Yes | TGD-validated PROM; includes sexual function and satisfaction, plus neovaginal pain and caliber |
|  | Female Sexual Function Index (FSFI) | Rosen *et al.* (2000) | Yes | No | No | Validated for cis women; covers arousal, orgasm, lubrication, satisfaction; no TGD validation |
|  | Arizona Sexual Experience Scale (ASEX) | McGahuey *et al.* (2000) | Yes | No | No | Brief 5-item scale (drive, arousal, orgasm, satisfaction); generic; no TGD validation |
|  | Brief Index of Sexual Functioning for Women (BISF-W) | Taylor *et al.* (1994) | Yes | No | No | Multidomain sexual function; developed for cis women; no TGD validation |
|  | Brief Sexual Function Inventory (BSFI) | O’Leary *et al.* (1995) | Yes | No | No | Male sexual function instrument; no adaptation for TGD; sometimes used in transmasculine cohorts |
|  | Changes in Sexual Functioning Questionnaire (CSFQ) | Bobes *et al.* (2000) | Yes | No | No | Evaluates desire through orgasm; no TGD validation; occasionally used in hormone therapy studies |
|  | Costantino Sexual Function Questionnaire | Costantino *et al.* (2013) | Yes | No | Yes | Ad hoc questionnaire developed for trans men; targets hormonal therapy and GAS; not validated |
|  | Derogatis Sexual Functioning Inventory — Fantasy Scale (DSFI) | Derogatis *et al.* (1979) | No | No | No | Measures sexual fantasy; not validated for TGD; not directly relevant to post-gGAS function |
|  | Female Sexual Distress Scale — Revised (FSDS-R) | Derogatis *et al.* (2002) | No | No | No | Measures sexual distress; not TGD validated; secondary relevance |
|  | Female Sexual Dysfunction Index — 6 item (FSDI-6) | Maseroli *et al.* (2016) | Yes | No | No | Brief screen for female sexual dysfunction; not TGD validated |
|  | Golombok Rust Inventory of Sexual Satisfaction (GRISS) | Rust *et al.* (1985) | Yes | No | No | Addresses relationship sexual satisfaction and difficulties; generic; no TGD validation |
|  | Multidimensional Sexuality Questionnaire (MSQ) | Snell *et al.* (1993) | Yes | No | No | Covers multidimensional sexual functioning and attitudes; no TGD validation; sometimes used for baseline sexuality |
|  | New Sexual Satisfaction Scale (NSSS) | Stulhofer *et al.* (2010) | Yes | No | Yes | TGD validated; assesses personal and relational sexual satisfaction comprehensively |
|  | PROMIS Sexual Function and Satisfaction Measures v2.0 (PROMIS SexFS v2.0) | Weinfurt *et al.* (2015) | Yes | No | Yes | Item bank approach; adaptable; not TGD validated but domains relevant to gGAS |
|  | Sexual Function Index — Gender Spectrum (SFI-GS) | Spencer *et al.* (2017) | Yes | No | Yes | Developed for gender-diverse populations; covers multiple sexual domains; limited validation data |
|  | Sexual Life Quality Questionnaire (SLQQ) | Woodward *et al.* (2002) | Yes | No | Yes | Developed for ED interventions; sometimes applied to neovaginal outcomes; not TGD validated |
|  | Sexual Satisfaction Scale for Women (SSS-W) | Meston *et al.* (2005) | Yes | No | Yes | Focused on satisfaction/distress across sexual domains; not TGD validated but applicable |
|  | Short Questionnaire for Self-Evaluation of Vaginoplasty (SQSV) | Buncamper *et al.* (2015) | Yes | No | Yes | Postoperative instrument including orgasm attainment; no validation; gGAS-specific |
|  | Wierckx Sexual Function Questionnaire | Wierckx *et al.* (2011) | Yes | No | Yes | Used in transmasculine cohorts post-GAS; covers sexual activity and satisfaction; not formally validated |
|  | Sexual Satisfaction and Function (SatisFunction) survey | Sahmoud *et al.* (2025) | Yes | No | Yes | Assessment of satisfaction across 8 domains including desire, arousal, orgasm, satisfaction, pain, anatomy; TGD-specific, community-informed tool; only content validity, full psychometric validation pending |
|  | Patient-Reported Outcomes in Genital Reconstructive Surgeries (PROGRESS) | *Kanthabalan A. et al.* (2025) | Yes | Yes | Yes | Comprehensive sexual function domain, significant post-operative improvements, captures satisfaction and functional outcomes; validated in TGD patients undergoing fGRS |
|  | GENDER-Q — Sexual Well-Being Scale | Kaur *et al.* (2025) | No | Yes | Yes | TGD-developed and validated; sexual function captured as part of the broader well-being construct |
|  | GENDER-Q — Vagina Scale | Kaur *et al.* (2025) | Yes | Yes | Yes | TGD-specific and validated; includes satisfaction and function of neovagina explicitly |
| Erogenous sensibility of the genitals | Modified Female Sexual Function Index for transgender women (oMtFSFI) | Vedovo *et al.* (2020) | No | Partial (only Italian and French validation) | Yes | Adapted FSFI; includes limited items indirectly related to genital sensitivity; partial TGD validation |
|  | Post-Affirming Surgery Form and Function Individual Reporting Measure (AFFIRM) | Huber *et al.* (2021) | No | Yes | Yes | TGD-validated PROM; includes some items on vaginal pain and caliber but not direct erotic sensation |
|  | Self-Assessment of Genital Anatomy and Sexual Function — Feminizing (L-SAGASF-F) | Schober *et al.* (2015) | No | No | Yes | Evaluates anatomy and sexual function; includes some sensitivity ratings; not validated |
|  | Short Questionnaire for Self-Evaluation of Vaginoplasty (SQSV) | Buncamper *et al.* (2015) | No | No | Yes | Simple postoperative PROM; genital specificity but limited focus on erotic sensation |
|  | Patient-Reported Outcomes in Genital Reconstructive Surgeries (PROGRESS) | *Kanthabalan A. et al.* (2025) | No | Yes | Yes | Captures subjective genital sensory experience during sexual activity, including arousal and orgasm, but not erogenous sensation; validated in TGD patients undergoing fGRS |
|  | GENDER-Q — Sexual Well-Being Scale | Kaur *et al.* (2025) | No | Yes | Yes | TGD-validated PROM; includes sexual satisfaction but not direct measurement of erogenous sensibility |
|  | GENDER-Q – Female Genital Sensation Scale (5 items) | Kaur et al. (2024) | Yes | Yes | Yes | TGD-specific, qualitatively developed, cognitively tested, and field-tested; assesses vaginal, labial and clitoral sensation; items removed from final GENDER-Q scales during Rasch-based reduction for statistical reasons |

gGAS: genital gender-affirming surgery; HRQoL: health-related quality of life; QoL: quality of life; TGD: transgender and gender-diverse; PCS: physical component summary; MCS: mental component summary; PROM: patient-reported outcome measure; fGRS: feminizing genital reconstructive surgery; ED: erectile dysfunction.

# Table S5. Quality assessment of identified patient-reported outcome measures (PROMs) for the core outcomes in masculinizing genital gender-affirming surgery.

| **Core Outcome** | **Identified PROM** | **Reference** | **Criterion A (Are the relevant aspects of the outcome measured?)** | **Criterion B (Is it validated for the target population?)** | **Criterion C (Is it relevant for use in context of genital gGAS?)** | **Rationale** |
| --- | --- | --- | --- | --- | --- | --- |
| Sensibility in the neo-phallus | Sexual Functioning Index – Gender Spectrum (SFI-GS) | Spencer & Vencill (2017) | No | No | Yes | Covers broad sexual function (activity, interest, arousal, orgasm, pain) but not tactile/temperature/erogenous sensation directly; designed for GAC contexts |
|  | Sexual Health Inventory for Men (SHIM/IIEF-5) | Rosen *et al.* (1999) | No | No | No | Screens erectile dysfunction only; no sensory items; cis-male validation only |
|  | Wierckx sexual-functioning questionnaire (ad-hoc) | Wierckx *et al.* (2011) | No | No | Yes | Trans-men surgical cohort tool; broad function but no explicit tactile/erogenous sensation items |
|  | Self-Assessment of Genital Anatomy & Sexual Function – Male (SAGASF-M) | Schober *et al.* (2009) | Yes | No | Yes | Site-specific ratings of erotic and pain sensitivity, orgasm intensity/effort across glans/shaft/scrotum/perineum; cis-male validation only but directly measures sensation |
|  | GENDER-Q – Penis Sensation Scale | Kaur *et al.* (2025) | Yes | Yes | Yes | TGD-developed/validated scale; directly assesses tactile and erogenous sensation in the neo-phallus; purpose-built for GAS |
| Ability to achieve orgasm | Arizona Sexual Experience Scale (ASEX) | McGahuey *et al.* (2000) | Yes | No | Yes | Includes items on orgasm ability and satisfaction; generic tool, not gGAS-specific |
|  | Brief Sexual Function Inventory (BSFI) | O’Leary *et al.* (1995) | No | No | Yes | Covers erection/ejaculation/satisfaction; orgasm not directly scored |
|  | Changes in Sexual Functioning Questionnaire (CSFQ) | Bobes *et al.* (2000) | Yes | No | No | Has an orgasm domain; designed for med-side-effects, not gGAS |
|  | Constantino Questionnaire (ad hoc) | Costantino *et al.* (2013) | Yes | No | Yes | Asks orgasm among other domains; developed in trans men cohort, no psychometric validation |
|  | Male Sexual Function Index (MSFI) | Kalmbach *et al.* (2015) | Yes | No | Yes | Includes orgasm items; cis-male validation, not TGD |
|  | Male Sexual Function Questionnaire (MSF-4) | Marquis & Marrel (2001) | Yes | No | No | 4-item screener including orgasm; brief, non-gGAS context |
|  | Male Sexual Health Questionnaire (MSHQ) | Rosen *et al.* (2004) | No | No | Yes | Covers broad male function; orgasm intensity not explicit |
|  | Multidimensional Sexual Self-Concept Questionnaire (MSSCQ) | Snell (1998) | No | No | No | Covers attitudes/self-concept; not physiological orgasmic function |
|  | Multidimensional Sexuality Questionnaire (MSQ) | Snell *et al.* (1993) | No | No | No | Emphasizes psychological facets; not orgasm ability |
|  | New Sexual Satisfaction Scale (NSSS) | Stulhofer *et al.* (2010) | No | No | Yes | Addresses satisfaction (including orgasm satisfaction) but not functional ability |
|  | PROMIS Sexual Function & Satisfaction v2.0 (PROMIS SexFS) | Weinfurt *et al.* (2015) | Yes | No | Yes | Item bank includes orgasm ability/pleasure; generic, no TGD validation |
|  | Quality of Sexual Experiences Scale (QSE) | Sanders *et al.* (2013) | No | No | No | Addresses event-level pleasure; doesn’t test orgasm capacity; no TGD validation; not applicable to gGAS |
|  | Sexual Function Questionnaire – Version 1 (SFQ-V1) | Quirk *et al.* (2002) | Yes | No | No | Female SD tool; includes orgasm but not applicable to masculinizing gGAS |
|  | Sexual Functioning Index – Gender Spectrum (SFI-GS) | Spencer & Vencill (2017) | Yes | No | Yes | Includes an orgasm domain; no published validation yet |
|  | Sexual Health Inventory for Men (SHIM/IIEF-5) | Rosen *et al.* (1999) | No | No | No | ED screener; no orgasm items |
|  | Symptom Checklist-90 (sexual dysfunction subscale) (SCL-90 SD) | Derogatis & Unger (2010) | No | No | No | Addresses distress about sexual problems; not orgasm performance |
|  | Wierckx sexual-functioning questionnaire (ad hoc) | Wierckx *et al.* (2011) | Yes | No | Yes | Asks orgasm ability/difficulty; developed in trans men surgical cohort, no validation |
|  | Self-Assessment of Genital Anatomy & Sexual Function – Male (SAGASF-M) | Schober *et al.* (2009) | Yes | No | Yes | Rates orgasm intensity/effort and site-specific sensitivity; cis-male validation |
|  | International Index of Erectile Function - 15-item (IIEF-15) | Rosen *et al.* (1997) | Yes | No | Yes | Has a two-item orgasmic function domain; no TGD validation |
|  | Golombok-Rust Inventory of Sexual Satisfaction (GRISS) | Rust & Golombok (1985) | No | No | No | Addresses relationship satisfaction/dysfunction; not explicit orgasm capacity |
|  | Sexual Quotient Scale – Male (SQSV) | Abdo (2007) | Yes | No | Yes | Includes orgasm item among others; cis-male validation only |
|  | GENDER-Q – Orgasm Scale | Kaur *et al.* (2025) | Yes | Yes | Yes | Purpose-built for GAS, TGD-validated; directly measures neogenital orgasmic function |
| Sexual well-being | Arizona Sexual Experience Scale (ASEX) | McGahuey *et al.* (2000) | No | No | Yes | Focuses on sexual function (drive, arousal, orgasm); does not assess emotional/relational well-being, but useful as a dysfunction screener |
|  | Constantino Questionnaire (ad hoc) | Costantino *et al.* (2013) | No | No | Yes | Covers sexual function in trans men; does not include mental or relational well-being; ad-hoc, no validation |
|  | Derogatis Fantasy Scale (DFS) | Derogatis *et al.* (1979) | No | No | No | Measures fantasy frequency/content only; not a well-being tool |
|  | Gay and Lesbian Relationship Satisfaction Scale (GLRSS) | Belous *et al.* (2016) | No | No | Yes | Relationship satisfaction measure; captures relational aspects, not sexual function; validated in LGB samples |
|  | Golombok-Rust Inventory of Sexual Satisfaction (GRISS) | Rust & Golombok (1985) | No | No | Yes | Evaluates sexual satisfaction/dysfunction within couples; covers some relational satisfaction but omits broader well-being |
|  | Male Sexual Function Index (MSFI) | Kalmbach *et al.* (2015) | No | No | Yes | Assesses sexual function but excludes psychological/social well-being |
|  | Male Sexual Function Questionnaire (MSF-4) | Marquis & Marrel (2001) | No | No | Yes | Brief male function screener; no psychosocial content |
|  | Male Sexual Health Questionnaire (MSHQ) | Rosen *et al.* (2004) | No | No | Yes | Covers broad male function, no mental/relational well-being |
|  | Multidimensional Sexual Self-Concept Questionnaire (MSSCQ) | Snell (1998) | No | No | No | Targets psychological domains (confidence, anxiety, satisfaction) but not physical or surgical aspects; general validation |
|  | Multidimensional Sexuality Questionnaire (MSQ) | Snell *et al.* (1993) | No | No | Yes | Covers some psychological and attitudinal aspects; not comprehensive for well-being |
|  | New Sexual Satisfaction Scale (NSSS) | Stulhofer *et al.* (2010) | No | No | Yes | Measures personal and relational sexual satisfaction; does not assess psychological well-being |
|  | PROMIS Sexual Function & Satisfaction v2.0 (PROMIS SexFS) | Weinfurt *et al.* (2015) | No | No | Yes | Generic sexual function/satisfaction tool; lacks psychosocial depth |
|  | Self-Esteem and Relationship Questionnaire (SEAR-Q) | Cappelleri *et al.* (2004) | No | No | Yes | Targets self-esteem and sexual relationships in men with ED; captures psychosocial impacts |
|  | Sexual Arousal and Desire Inventory (SADI) | Toledano & Pfaus (2006) | No | No | Yes | Assesses arousal/desire dimensions; not overall well-being, but postoperative changes can be detected |
|  | Sexual Desire Inventory (SDI) | Spector *et al.* (1996) | No | No | Yes | Measures libido (partnered/solitary), not well-being |
|  | Sexual Function Questionnaire – Version 1 (SFQ-V1) | Quirk *et al.* (2002) | No | No | No | Female sexual dysfunction tool; irrelevant to masculinizing gGAS |
|  | Sexual Functioning Index – Gender Spectrum (SFI-GS) | Spencer & Vencill (2017) | No | No | Yes | Covers function, not broader well-being; no validation |
|  | Sexual Health Inventory for Men (SHIM / IIEF-5) | Rosen *et al.* (1999) | No | No | No | ED screener; no psychosocial or relational content |
|  | Sexual Life Quality Questionnaire (SLQQ) | Woodward *et al.* (2002) | No | No | Yes | Focuses on sexual QoL in ED patients; lacks transgender validation |
|  | Sexual Satisfaction Scale for Women (SSS-W) | Meston & Trapnell (2005) | No | No | No | Female satisfaction tool; not applicable to trans men |
|  | Symptom Checklist-90 (sexual dysfunction subscale) (SCL-90 SD) | Derogatis & Unger (2010) | No | No | Yes | Psychological distress measure with some sexual items; not positive well-being but can track distress post-surgery |
|  | Wierckx sexual-functioning questionnaire | Wierckx *et al.* (2011) | No | No | Yes | Covers sexual activity/satisfaction, not emotional well-being; developed within trans men surgical cohort but not validated |
|  | Modified hypospadias questionnaire | Bubanj *et al.* (2004) | No | No | Yes | Male sexual function after hypospadias repair; no mental/social domains, but surgically relevant |
|  | Brief Sexual Function Inventory (BSFI) | O’Leary *et al.* (1995) | No | No | Yes | Male function tool; no psychosocial focus |
|  | International Index of Erectile Function - 15-item (IIEF-15) | Rosen *et al.* (1997) | No | No | Yes | Covers sexual function domains; does not assess sexual well-being |
|  | Changes in Sexual Functioning Questionnaire (CSFQ) | Bobes *et al.* (2000) | Yes | No | Yes | Covers multiple sexual function domains, some psychological elements; not a full well-being measure |
|  | Erectile Dysfunction Inventory of Treatment Satisfaction (EDITS) | Althof *et al.* (1999) | No | No | No | Measures satisfaction with ED treatment; not relevant to well-being after gGAS |
|  | Erection Hardness Score (EHS) | Mulhall *et al.* (2007) | No | No | Yes | Erection rigidity rating; not psychosocial, but clinically relevant post-surgery |
|  | Quality of Sexual Experiences Scale (QSE) | Sanders *et al.* (2013) | No | No | No | Measures event-level sexual quality; does not cover broader well-being |
|  | Sexual Quotient Scale – Male (SQSV) | Abdo (2007) | No | No | Yes | Covers sexual domains (desire, erection, orgasm); no psychological or social well-being |
|  | Self-Assessment of Genital Anatomy & Sexual Function – Male (SAGASF-M) | Schober *et al.* (2009) | No | No | Yes | Focuses on genital sensation and orgasm; not well-being |
|  | GENDER-Q – Sexual Well-Being Scale | Kaur *et al.* (2025) | Yes | Yes | Yes | Purpose-built for GAS, TGD-validated; directly measures sexual well-being postoperatively |
| Satisfaction with neogenital aesthetic result | Surgical Satisfaction Questionnaire (SSQ-8) | Baker (1991) | No | No | Yes | Global “surgical results” item; does not isolate neo-genital appearance, but captures overall postoperative satisfaction |
|  | Male Genital Self-Image Scale (MGSIS-7) | Herbenick *et al.* (2013) | Yes | No | Yes | Directly measures satisfaction with genital appearance (size/shape/overall); validated in cis men only |
|  | Global Response Assessment (GRA) | — | No | No | Yes | Single global improvement item; not aesthetics-specific, but reflects overall perceived change |
|  | Body Image Scale for Transsexuals (BIS) | Lindgren & Pauly (1975) | Yes | Yes | Yes | TGD-developed body image tool with a genitals item; not neo-genital–specific, but applicable post-gGAS |
|  | Index of Male Genital Image (IMGI) | Davis *et al.* (2013) | Yes | No | Yes | Penis/scrotum appearance domains; robust in cis men; conceptually fits neo-genital aesthetics |
|  | Self-Assessment of Genital Anatomy & Sexual Function – Male (SAGASF-M) | Schober *et al.* (2009) | Yes | No | Yes | Includes site-specific appearance ratings (glans/shaft/scrotum); validated in healthy cis men |
|  | GENDER-Q – Penis / Glans / Scrotum Scales | Kaur *et al.* (2025) | Yes | Yes | Yes | Purpose-built, TGD-validated scales that directly assess neo-genital aesthetic satisfaction after GAS |
| Donor-site morbidity | Patient and Observer Scar Assessment Scale (POSAS) | Draaijers *et al.* (2004) | Yes | No | Yes | Captures pain, pruritus, thickness, pliability, color; strong psychometrics across scar populations; not TGD-specific |
|  | Vancouver Scar Scale (VSS) | Sullivan *et al.* (1990) | No | No | Yes | Rates vascularity, height, pliability, pigmentation only; omits symptoms like pain/itch |
|  | Baecke Habitual Physical Activity Questionnaire | Baecke *et al.* (1982) | No | No | No | Tracks lifestyle activity; does not address donor-site sensations or scar morbidity |
|  | Disabilities of the Arm, Shoulder and Hand (DASH) | Wylie *et al.* (2014) | No | No | Yes | Measures UE disability/symptoms; useful for radial-forearm donor sites; not designed for scars per se |
|  | Quick Disabilities of Arm, Shoulder & Hand (QuickDASH) | Gummesson *et al.* (2006) | No | No | Yes | 11-item version of DASH; functional, not symptom-of-scar focused; useful for UE donor site |
|  | SCAR-Q | Klassen *et al.* (2018) | Yes | No | Yes | Patient-reported appearance, symptoms and psychosocial impact; validated broadly, not TGD-specific |
|  | Patient-Reported Impact of Scars Measure (PRISM) | Brown *et al.* (2010) | No | No | Yes | Two unidimensional scales (symptoms and QoL); lacks appearance domain; still relevant to donor-site burden |
|  | Bock Scar QoL Questionnaire | Bock *et al.* (2006) | No | No | Yes | Focuses on pain, pruritus, mobility restriction, psychosocial effects in raised scars |
|  | Patient Scar Assessment Questionnaire (PSAQ) | Durani *et al.* (2009) | Yes | No | Yes | Multi-domain patient scale; applicable to linear donor scars (e.g., ALT, RFF) |
|  | GENDER-Q – Donor Site – Adverse effects | Kaur *et al.* (2025) | Yes | Yes | Yes | TGD-developed/validated; directly assesses donor-site morbidity after GAS |
| Ability to void in a standing position | Post-Phalloplasty Urinary Function Test (PP UFT) | Liu *et al.* (2022) | Yes | No | Yes | Specifically designed for trans men post-phalloplasty, with items on voiding position, stream control, postvoid dribbling, and QoL impact; not validated yet |
|  | GENDER-Q – Standing Voiding Short Scale (2 items, field-tested version) | Kaur *et al.* (2024) | Yes | Yes | Yes | TGD-specific items; cognitively tested, and field-tested within the GENDER-Q; removed from the longer Penis scale during Rasch-based reduction for statistical rather than content-validity reasons |

PROM: patient-reported outcome measure; GAC: gender-affirming care; TGD: transgender and gender-diverse; gGAS: genital gender-affirming surgery; SD: sexual dysfunction; ED: erectile dysfunction; LGB: lesbian, gay, bisexual; UE: upper extremities; ALT: antero-lateral thigh; RFF: radial forearm flap.

# Table S6. Feasibility of identified patient-reported outcome measures (PROMs) to assess the core outcomes in feminizing genital gender-affirming surgery.

| **Core Outcome** | **Instrument** | **Citation** | **Applicability to Core Outcome** | **Respondent Burden** | **Target Population Involvement** | **Licensing Requirements** | **Costs** | **Recommended Time of Assessment** |
| --- | --- | --- | --- | --- | --- | --- | --- | --- |
| Health-related quality of life | Short Form Health Survey (SF-36) | Ware and Sherbourne (1992) | Yes (assesses 8 HRQoL domains; broad post-surgical QoL; not gGAS-specific) | Moderate (36 items) | High (general-population development appropriate for a general outcome; no TGD involvement) | Free for v1.0 (RAND); v2 requires licence (QualityMetric) | None for v1.0; v2 license fee (undisclosed) | Pre-surgery; 3–6 months and 12 months post-op |
|  | Short Form Health Survey (SF-12) | Ware et al. (1996) | Yes (captures PCS/MCS HRQoL summaries; not gGAS-specific) | Low (12 items) | High (general-population development appropriate for a general outcome; no TGD involvement) | Licence required (QualityMetric) | License fee typically required | Baseline; 3–6 months post-op when brief HRQoL overview is needed |
|  | WHOQOL-100 | WHOQOL Group (1998) | Yes (multidomain QoL; broad assessment; not gGAS-specific) | High (100 items) | High (general-population development appropriate for a general outcome; international field centres; not TGD-specific) | User licence required (WHOQOL group) | No fee for non-commercial research | Pre-surgery; annual follow-up for comprehensive QoL profiling |
|  | WHOQOL-BREF | WHOQOL Group (1998) | Yes (brief multidomain QoL; not gGAS-specific) | Moderate (26 items) | High (general-population development appropriate for a general outcome; no TGD involvement) | User licence required (WHOQOL group) | No fee for non-commercial research (user agreement) | Baseline; 6 and 12 months post-op |
|  | PROMIS – Global Health | Hays *et al.* (2009) | Yes (multidomain HRQoL with Global Physical & Mental Health T-scores; not gGAS-specific) | Low (10 items) | High (general-population development appropriate for a general outcome; no TGD involvement) | No license needed for paper PDFs; electronic platforms/APIs may require agreements | None for instrument/scoring | Baseline; 3–6 months; 12 months post-op (general HRQoL follow-up windows; not PROMIS-specific) |
|  | Essen Transgender Quality of Life Inventory (ETLI) | Rücker et al. (2025) | Partial (TGD-specific HRQoL; directly applicable after gGAS but risk of being overly specific and failing to capture general outcomes which can be influenced by many factors not directly affected by surgery) | Moderate (30 items) | Moderate (developed/validated within TGD participants only) | Non-commercial use with citation; permission needed for commercial use | None for non-commercial use | Pre-surgery; 6–12 months post-op |
|  | GENDER-Q — HRQoL concept | Kaur et al. (2025) | Partial (GAS-specific HRQoL across multiple dimensions but risk of being overly specific and failing to capture general outcomes which can be influenced by many factors not directly affected by surgery) | High (≈61 items) | Moderate (co-developed with TGD people only and clinicians) | License required via platform | No cost for research use | Across care pathway: baseline and post-treatment follow-ups |
| Genital gender congruence | GENDER-Q — Vagina scale | Kaur et al. (2025) | No (only one item addresses genital gender congruence) | Low (10 items) | High (TGD development/validation) | License required via platform | No cost for research use | Baseline and post-treatment follow-ups |
|  | Genital Gender-affirmation items from previous GENDER-Q field-test version | Kaur *et al.* (2024) | Yes (captures genital gender affirmation across the neogenitals) | Low (2 items) | High (TGD development/validation; dropped later due to poor fit to the other scales) | License required via platform | No cost for research use | Baseline and post-treatment follow-ups |
| Satisfaction with surgical result | Post-Affirming Surgery Form and Function Individual Reporting Measure (AFFIRM) | Huber et al. (2021) | Yes (post-operative symptoms and satisfaction across appearance/urologic/gynecologic domains) | Moderate (33 items) | High (developed/validated with transgender women) | Developed by authors; no public licence | No fees reported | ≈1 year post-op; baseline/early follow-up possible |
|  | GENDER-Q — Treatment outcome scale | Kaur et al. (2025) | Yes (items directly evaluate satisfaction with GAS outcomes) | Low (≈10 items) | High (TGD development/validation) | License required via platform | No cost for research use | Baseline and post-treatment follow-ups |
| Satisfaction with aesthetic outcome | Post-Affirming Surgery Form and Function Individual Reporting Measure (AFFIRM) | Huber et al. (2021) | Yes (appearance domain captures aesthetic satisfaction) | Moderate (33 items) | High (developed/validated with transgender women) | Developed by authors; no public license | No fees reported | ≈1 year post-op; can be used pre-op and during recovery |
|  | Patient-Reported Outcomes in Genital Reconstructive Surgeries (PROGRESS) | Kanthabalan A. et al. (2025) | Yes (cosmetic domain evaluates perceived appearance, distress, avoidance, social impact) | Moderate (cosmetic domain: 10 items; part of full 45-item questionnaire) | High (developed/validated with TGD focus groups and clinical experts) | Open access (CC-BY 4.0); no license required | None for clinical/research use | Baseline, 10 weeks, 52 weeks post-op to track changes in appearance satisfaction |
|  | GENDER-Q — Vagina, Labia and Clitoris scales | Kaur et al. (2025) | Yes (items directly evaluate satisfaction with aesthetic outcomes) | Moderate (28 items) | High (TGD development/validation) | License required via platform | No cost for research use | Baseline and post-treatment follow-ups |
| Satisfaction with neo-genital sexual function | Post-Affirming Surgery Form and Function Individual Reporting Measure (AFFIRM) | Huber et al. (2021) | Yes (captures satisfaction with function and symptoms including vaginal pain/caliber) | Moderate (33 items) | High (developed/validated with transgender women) | Developed by authors; no public license | No fees reported | ≈1 year post-op; baseline and early post-op possible |
|  | Patient-Reported Outcomes in Genital Reconstructive Surgeries (PROGRESS) | Kanthabalan A. et al. (2025) | Yes (dedicated sexual function domain covering desire, arousal, orgasm, pain, satisfaction) | Moderate (sexual domain: 15 items; part of full 45-item questionnaire) | High (developed & validated specifically in transgender women undergoing fGRS) | Open access (CC-BY 4.0); no special license required | None for clinical/research use | Baseline (pre-op), 10 weeks, and 52 weeks post-op (validated time points) |
|  | GENDER-Q — Vagina scale | Kaur et al. (2025) | Yes (relevant items on neovaginal sexual function; not full fgGAS scope) | Low (10 items) | High (TGD development/validation) | License required via platform | No cost for research use | Baseline and post-treatment follow-ups |
| Erogenous sensibility of the genitals | GENDER-Q – Female Genital Sensation Scale (5 items) | Kaur et al. (2024) | Yes (multiple items asking on neovaginal erogenous sensation) | Low (5 items) | High (TGD development/validation) | License required via platform | No cost for research use | Baseline and post-treatment follow-ups |

HRQoL: health-related quality of life; QoL: quality of life; gGAS: genital gender-affirming surgery; TGD: transgender and gender-diverse; PCS: physical component summary; MCS: mental component summary; fGRS: feminizing genital reconstructive surgery.

# Table S7. Feasibility of identified patient-reported outcome measures (PROMs) to assess the core outcomes in masculinizing genital gender-affirming surgery.

| **Core Outcome** | **Instrument** | **Citation** | **Applicability to Core Outcome** | **Respondent Burden** | **Target Population Involvement** | **Licensing Requirements** | **Costs** | **Recommended Time of Assessment** |
| --- | --- | --- | --- | --- | --- | --- | --- | --- |
| Sensibility in the neo-phallus | GENDER-Q — Penis sensation scale | Kaur et al. (2025) | Yes (focuses on tactile and erogenous sensation of the neo-phallus) | Low (11 items) | High (developed with TGD people and clinicians) | License required via platform | No cost for research use | Baseline and post-treatment follow-ups |
| Ability to achieve orgasm | GENDER-Q — Orgasm scale | Kaur et al. (2025) | Yes (assesses neogenital orgasmic function) | Low (8 items) | High (TGD development and validation) | License required via platform | No cost for research use | Baseline and post-treatment follow-ups |
| Sexual well-being | GENDER-Q — Sexual well-being scale | Kaur et al. (2025) | Yes (directly assesses sexual well-being after GAS) | Low (12 items) | High (TGD development and validation) | License required via platform | No cost for research use | Baseline and post-treatment follow-ups |
| Satisfaction with neo-genital aesthetic result | Body Image Scale for Transsexuals (BIS) | Lindgren and Pauly (1975) | Yes (includes genital item; assesses body satisfaction broadly rather than phallus/scrotum specifically) | Moderate (30 items) | Yes (developed in transgender populations; not validated) | Public domain | None | Post-surgery; can be used longitudinally |
|  | GENDER-Q — Penis, Glans and Scrotum scales | Kaur et al. (2025) | Yes (assesses satisfaction with aesthetic outcomes of neo-phallus and scrotum) | Moderate (31 items) | High (TGD development and validation) | License required via platform | No cost for research use | Baseline and post-treatment follow-ups |
| Ability to void in a standing position | GENDER-Q – Standing Voiding Short Scale (2 items, field-tested version) | Kaur et al. (2024) | Yes (two items directly asking about ability to urinate in a standing position) | Low (2 items) | High (TGD development and validation) | License required via platform | No cost for research use | Baseline and post-treatment follow-ups |
| Donor site morbidity | GENDER-Q — Donor site – Adverse effects scale | Kaur et al. (2025) | Yes (assesses symptoms such as pain, tightness, and discomfort at donor site) | Low (12 items) | High (TGD development and validation) | License required via platform | No cost for research use | Baseline and post-treatment follow-ups |

TGD: transgender and gender-diverse; GAS: gender-affirming surgery.

# Table S8. Feasibility of clinical OMIs for core outcomes applicable to both feminizing and masculinizing genital gender-affirming surgery.

| **Outcome** | **OMI** | **Description** | **Patient / Admin Burden** | **Routine Use** | **Cost** | **Recommended Assessment Time** |
| --- | --- | --- | --- | --- | --- | --- |
| Additional surgery | Physical exam + history (structured follow-up visits for functional/aesthetic, Clavien–Dindo for complications) | Structured clinical assessment including dilation adherence, urinary and bowel function, and physical examination to identify functional, aesthetic, or complication-related indications for revision surgery. | Patient: moderate (multiple visits); Admin: low | Yes (routine follow-up) | Low | 2 week, 3 months, 6 months, 9 months, 1 year, annually |
|  | Clinical documentation | Standardized recording of indications and need for additional surgery based on complications, functional issues, or patient dissatisfaction. | Patient: low; Admin: low | Yes | Low | At revision or follow-up visit |
|  | Clavien–Dindo classification | Standard grading of complications by therapeutic consequence. | Patient: low; Admin: low | Yes (widely used) | Low | At time of complication or re-operation |

# Table S9. Feasibility of clinical OMIs for core outcomes in feminizing genital gender-affirming surgery.

| **Outcome** | **OMI** | **Description** | **Patient / Admin Burden** | **Routine Use** | **Cost** | **Recommended Assessment Time** |
| --- | --- | --- | --- | --- | --- | --- |
| Loss of neovaginal tissue lining | Clinical observation | Internal physical examination assessing tissue viability, infection, and granulation to detect ischemic or inflammatory loss of neovaginal lining. | Patient: moderate; Admin: low | Yes | Low | 2 weeks, 3 months, 6 months, 9 months, 1 year, annually (internal exam ~3 months) |
|  | MRI | Radiological evaluation of neovaginal depth, contours, angle, and rectovaginal thickness to identify tissue loss, stenosis, prolapse, or fistula. | Patient: moderate; Admin: high | No (complex cases / research) | High | ~3 mo post-surgery (research) or when complications suspected |
|  | Clinician assessment using dilators/rod (generic, customized, standardized, Hagar, Young's, size 21 Hegar-Mosquito dilator) | Depth and width measurement of the neovaginal canal using standardized dilators to assess canal dimensions and healing progression. | Patient: moderate; Admin: low | Yes | Low–moderate | 2 weeks, 3 months, 6 months, 9 months, 1 year; final depth at ~1 year |
|  | Digital examination or patient observation | Manual assessment of canal depth and width through finger palpation, supplemented by patient-reported observations when applicable. | Patient: low; Admin: low | Yes | Low | At each follow-up visit |
|  | Patient-reported maximum dilator depth and size | Self-reported maximum dilator depth and size during home dilation or clinic visits. | Patient: moderate; Admin: low | Occasionally | Low | During follow-up visits or home dilation |
|  | Transparent plastic molds | Transparent plastic molds used to measure neovaginal depth and width. | Patient: moderate; Admin: moderate | No (research) | Moderate | Single postoperative measurement (3–12 months) |
|  | Clavien–Dindo (re-operation) | Complication grading system applied when neovaginal tissue loss necessitates re-operation, providing standardized severity classification. | Patient: low; Admin: low | Yes | Low | At time of complication or re-operation |
| Neo-vaginal stenosis | Physical examination with dilators/speculum | Clinical assessment of vaginal length and width using dilators or speculum to detect narrowing (typically length < 12 cm, width < 3.5 cm). | Patient: moderate; Admin: low | Yes | Low | 3 months, 6 months, 9 months, 1 year, or when symptoms occur |
|  | MRI | Radiological evaluation of canal dimensions and scar bands to detect stenosis or associated complications. | Patient: moderate; Admin: high | No (research/suspected cases) | High | ~3 mo (research) or when stenosis suspected |
|  | Patient-reported subjective feeling of a narrow introitus (BISF-W, oMtFSFI) | Self-administered questionnaires evaluating sexual dissatisfaction, pain, and genital self-image related to perceived introital narrowing. | Patient: moderate; Admin: low | No (research) | Low | Baseline and follow-up (e.g., 3–6 months); oMtFSFI retest after 4 weeks |
|  | Transparent plastic molds | Transparent plastic molds used to measure neovaginal depth and width. | Patient: moderate; Admin: moderate | No | Moderate | Single assessment once healing stabilizes (6–12 months) |
|  | Clavien–Dindo (re-operation) | Complication grading system applied when stenosis requires surgical intervention, ensuring standardized reporting. | Patient: low; Admin: low | Yes | Low | At time of complication |
|  | Clinician assessment using dilators/rod | Measurement of neovaginal depth and width with standardized dilators to monitor healing and detect narrowing. | Patient: moderate; Admin: low | Yes | Low–moderate (dilator sets) | 2 weeks, 3 months, 6 months, 9 months, 1 year; final depth ~1 year |
|  | Digital examination | Manual palpation of canal depth and width during physical examination. | Patient: low; Admin: low | Yes | Low | At each follow-up visit |
|  | Measurement thresholds (e.g., depth < 10.9 cm, length < 12 cm, width < 3.5 cm, vagina depth < 2 cm) | Objective thresholds used to define clinically significant stenosis and guide decisions for further intervention. | Patient: moderate; Admin: low | Yes | Low | Follow-up visits (e.g., 3 months, 6 months, 1 year) |
|  | Patient-reported maximum dilator depth and size | Self-reported dilator insertion depth and size used to monitor canal patency during home dilation or when clinical assessment is limited. | Patient: moderate; Admin: low | Occasionally | Low | Follow-up visits or home dilation |
|  | Vaginal depth after complete healing | Measurement of final canal depth after epithelialization to confirm long-term outcome. | Patient: moderate; Admin: low | Yes | Low | ~1 year post-surgery |
| Stricture of neovaginal introitus | Physical examination (visual inspection and finger/dilator insertion) | Visual inspection and manual assessment of introital caliber using a finger or dilator to detect narrowing or bridging. | Patient: moderate; Admin: low | Yes | Low | Routine visits and when symptoms occur |
|  | MRI | Radiological assessment of introital caliber and associated complications when physical examination is inconclusive. | Patient: moderate; Admin: high | No | High | When needed for diagnostic clarification or surgical planning |
|  | Patient-reported subjective feeling of a narrow introitus (BISF-W, oMtFSFI) | Self-administered questionnaires evaluating sexual dissatisfaction, pain, and genital self-image related to introital narrowing. | Patient: moderate; Admin: low | No (research) | Low | Baseline and follow-up (≥3 months) |
|  | Transparent plastic molds | Transparent plastic molds used to measure neovaginal depth and width. | Patient: moderate; Admin: moderate | No | Moderate | Once healing has stabilized (6–12 months) |
|  | Clavien–Dindo classification | Complication grading system applied when introital stricture necessitates surgical intervention, allowing standardized reporting. | Patient: none; Admin: low | Yes | Low | At time of complication or re-operation |
|  | Clinician assessment using dilators/rod | Standardized dilator-based measurement of introital caliber during clinical follow-up. | Patient: moderate; Admin: low | Yes | Low | Follow-up visits |
|  | Digital examination | Manual finger assessment of introital opening during physical examination. | Patient: low; Admin: low | Yes | Low | Each follow-up visit |
|  | Patient-reported maximum dilator depth and size | Self-reported ability to pass dilators through the introitus during home dilation or clinic visits. | Patient: moderate; Admin: low | Occasionally | Low | During home dilation or clinic follow-up |
| Rectovaginal fistula | Physical examination (inspection of neovagina, digital rectal exam; ± speculum/proctoscope) | Clinical identification of feculent discharge or gas passage through the neovagina with confirmatory digital rectal and speculum assessment. | Patient: moderate; Admin: low | Yes | Low | When symptoms suggest fistula |
|  | Patient-reported symptoms | History of feculent discharge, flatus, or gas from the neovagina indicating possible fistulous communication. | Patient: low; Admin: low | Yes | Low | Any follow-up or symptom onset |
|  | MRI | Cross-sectional delineation of fistula tract, size, and relation to adjacent organs to guide management. | Patient: high; Admin: high | No | High | When exam is non-diagnostic or for surgical planning |
|  | Fistulogram / CT | Fluoroscopic or CT contrast visualization of fistulous pathway and external openings. | Patient: moderate; Admin: high | No | Moderate–high | When physical exam is inconclusive |
|  | Clavien–Dindo classification (re-operation) | Standard complication severity classification applied when fistula requires operative management. | Patient: low; Admin: low | Yes | Low | At complication or re-operation |
|  | Methylene-blue dye test (retention enema with vaginal tampon) | Confirmation of fistulous communication by tampon blue staining following rectal dye instillation. | Patient: moderate; Admin: low | No | Low | When physical exam cannot localize fistula |
|  | Intra-operative detection and closure (± colostomy) | Identification and repair of fistula during operative exploration, occasionally requiring fecal diversion. | Patient: high; Admin: high | No (not a tool) | High | During vaginoplasty or fistula repair |

MRI: magnetic resonance imaging; OMI: outcome measurement instrument; BISF-W: Brief Index of Sexual Functioning for Women; oMtFSFI: Operated Male-to-Female Sexual Function Index; CT: computed tomography.

# Table S10. Feasibility of clinical OMIs for core outcomes in masculinizing genital gender-affirming surgery.

| **Outcome** | **OMI** | **Description** | **Patient / Admin Burden** | **Routine Use** | **Cost** | **Recommended Assessment Time** |
| --- | --- | --- | --- | --- | --- | --- |
| Flap necrosis of the neo-phallus | Clinical assessment (inspection and palpation) | Bedside examination assessing flap color, capillary refill, and tissue viability to detect partial or complete necrosis. | Patient: low; Admin: low | Yes | Low | Daily during hospital stay; 1–2 weeks, 3–6 months, 1 year |
|  | Clavien–Dindo classification | Universal grading system categorizing necrosis and wound dehiscence severity based on required treatment. | Patient: none; Admin: low | Yes | Low | At complication or re-operation |
|  | Partial necrosis measurement (<10 % or cm²) | Estimation of affected flap area as a percentage or in cm² to document severity and progression. | Patient: low; Admin: low | Yes | Low | At necrosis detection (usually within 2 weeks) |
|  | Histological staining and examination | Microscopic analysis of biopsied necrotic tissue to characterize morphology and rule out infection or atypical pathology. | Patient: moderate; Admin: moderate | No (uncertain cases or research) | High (~US$128/specimen) | When infection or atypical pathology suspected |
|  | SKIN score | Grading system evaluating depth (epidermal to full thickness) and surface area of necrosis; validated in mastectomy flap necrosis but not in phalloplasty. | Patient: moderate; Admin: moderate | No (research) | Low | Early follow-up if applied in research; no standard schedule |
|  | Classification of partial and fat necrosis in DIEP flaps | Categorization of necrosis severity from minimal (<5 %) to complete (100 %), developed for breast reconstruction; not validated in phalloplasty. | Patient: moderate; Admin: moderate | No (research) | Low | At time of necrosis detection |
| Neo-urethral fistula | Clinical assessment (inspection and history) | Clinical evaluation combining patient-reported leakage, spraying, or dysuria with physical examination to detect ectopic urinary leakage sites. | Patient: low; Admin: low | Yes | Low | At catheter removal (≈2–6 weeks), 3 months, and until resolution or repair |
|  | Cystoscopy (flexible urethroscopy) | Endoscopic visualization of urethra and bladder to identify persistent fistulas or strictures when physical exam or imaging is inconclusive. | Patient: moderate; Admin: moderate | Occasionally | Moderate | When fistula persists >3 mo or before revision |
|  | Frequency voiding chart (FVC) | Recording of voiding times and volumes over 24–48 h to assess urinary patterns pre- and post-operatively. | Patient: moderate; Admin: low | Occasionally | Low | Pre-surgery; ≥1 year post-surgery; if urinary symptoms occur |
|  | Uroflowmetry with post-void residual (PVR) | Non-invasive measurement of urinary flow rate and residual volume to detect obstruction, fistula, or stricture. | Patient: low; Admin: low | Yes | Low | Pre-operative baseline; after catheter removal; ≥1 year |
|  | Retrograde urethrocystography (RUG) / voiding cystourethrogram (VCUG) | Contrast fluoroscopy to visualize urethral and bladder anatomy, delineating fistula or stricture location and length. | Patient: moderate; Admin: moderate | Occasionally (symptomatic patients or pre-revision planning) | Moderate | When uroflowmetry suggests obstruction or fistula; typically 3–6 mo post-surgery or pre-repair |
| Neo-urethral stricture | Clavien–Dindo classification (re-operation) | Complication grading system categorizing stricture severity based on the type of intervention required. | Patient: low; Admin: low | Yes | Low | At complication or re-operation |
|  | Clinical documentation (urinary retention requiring catheterization) | Recording of urinary retention episodes necessitating catheter insertion, indicating clinically significant stricture. | Patient: low; Admin: low | Yes | Low | Whenever retention occurs, typically within first months |
|  | Clinical examination (obstructive urinary symptoms) | History and physical examination assessing weak stream, straining, and post-void dribbling to identify signs of urethral narrowing. | Patient: low; Admin: low | Yes | Low | At each follow-up visit, especially after catheter removal or with new symptoms |
|  | Cystoscopy (flexible urethroscopy) | Endoscopic visualization of the urethral lumen to evaluate stricture location, severity, and plan intervention. | Patient: moderate; Admin: moderate | Occasionally | Moderate | When uroflowmetry or symptoms suggest obstruction; typically ≥3 mo post-surgery or pre-repair |
|  | Frequency voiding chart (FVC) | Serial recording of voiding frequency and volume to detect changes consistent with obstruction or altered bladder function. | Patient: moderate; Admin: low | Occasionally | Low | Pre-surgery and ≥1 year post-surgery |
|  | Uroflowmetry with post-void residual (PVR) | Non-invasive test measuring maximum urinary flow rate and residual urine volume; values <15 mL/s suggest obstruction and prompt further evaluation. | Patient: low; Admin: low | Yes | Low | Baseline; after catheter removal (~6 weeks); ≥1 year or sooner if symptoms develop |
|  | Retrograde urethrocystography (RUG) / voiding cystourethrogram (VCUG) | Contrast fluoroscopy visualizing urethral anatomy, stricture location, and length. | Patient: moderate; Admin: moderate | Occasionally (pre-operative evaluation before repair) | Moderate | When obstruction suspected; typically 3–6 mo post-surgery or before revision |

OMI: outcome measurement instrument; DIEP: deep inferior epigastric perforator.

# Supplementary S11. Discussion around tools to assess “Health-related quality of life” in the feminizing COS.

GENDER-Q Quality of Life construct and PROMIS Global Health Scale provide two relevant alternatives to assess the outcome health-related quality of life (HRQoL) after feminizing genital gender-affirming surgery, with difference in target population, sensibility to detect changes and administration burden.

GENDER-Q is a modular, gender-affirming care-specific PROM developed and psychometrically validated in a large, international transgender and gender diverse (TGD) population. Its Quality of Life construct is designed to capture HRQoL domains directly relevant to TGD individuals, including psychological well-being, social functioning, body image, sexual health, and satisfaction with gender-related outcomes. The instrument demonstrates strong test-retest reliability and construct validity, with scores correlating with self-reported psychological and social well-being. Its strengths are comprehensiveness, cultural relevance, and sensitivity to changes in HRQoL specific to gender-affirming interventions. Limitations include length and complexity, which may affect feasibility in routine clinical settings, and the need for digital administration in some contexts. Additionally, while the instrument captures multiple domains of HRQoL, it does not assess overall quality of life as a single global construct but rather evaluates each domain separately. ^1, 2^

PROMIS Global Health Scale is a generic HRQoL instrument developed for broad use across medical populations. It assesses global physical, mental, and social health using a brief set of items. Its strengths are brevity, ease of administration, robust psychometric properties in general populations, and the ability to enable comparison across patient populations undergoing analogous reconstructive procedures for different indications. Its recognized quality and increasing implementation in research further support its use in future studies. It lacks content specific to TGD experiences, such as gender congruence, body image, and unique psychosocial stressors related to gender-affirming surgery. While this may limit sensitivity for TGD-specific domains, it allows assessment of global health status and facilitates benchmarking across surgical populations ^3-6^

In summary, while the GENDER-Q Quality of Life construct offers superior sensitivity and relevance for evaluating HRQoL in TGD patients after feminizing genital gender-affirming surgery, a generic and brief tool like the PROMIS Global Health Scale may be more valuable to assess the outcome in the population. Its use supports widespread implementation of the COS and enables comparison not only within gender-affirming care but also with analogous reconstructive procedures performed for other indications.

# Supplementary S12. Discussion around tools to assess “Satisfaction with neo-genital sexual function” in the feminizing COS.

The operated Male-to-Female Sexual Function Index (oMtFSFI), Post-Affirming Surgery Form and Function Individual Reporting Measure (AFFIRM), Patient-Reported Outcomes in Genital Reconstructive Surgeries (PROGRESS), and GENDER-Q questionnaires each offer distinct approaches to measuring satisfaction with neo-genital sexual function in transgender women after feminizing genital gender surgery, with important differences in validity, reliability, and domain coverage.

oMtFSFI is specifically validated to assess sexual function in transgender women after gender-affirming surgery. It covers domains of sexual dissatisfaction, sexual pain, and genital self-image, with demonstrated internal consistency and test-retest reliability. Its content validity is high for the target population, and it directly assesses satisfaction with sexual function of the surgically created genitals. However, some limitations include assumptions about partnered or penetrative intercourse, which may not apply to all patients. The instrument has undergone cross-cultural adaptation and face validation, supporting its relevance and comprehensiveness for post-vaginoplasty sexual function. Terminology may require adaptation for broader populations. English language validation of the instruments is still lacking. ^7, 8^

AFFIRM is a broader patient-reported outcome measure developed for trans women post-genital surgery, with validated domains for appearance, urological, and gynecologic outcomes. While it includes items on clitoral sensation and satisfaction with vaginal caliber, its sexual satisfaction domain is less developed and not as psychometrically robust as other tools. Major strength lies in its multidimensional approach, with broader coverage of postoperative symptoms and quality of life, and moderate-to-good reliability, but its sexual function assessment is limited. ^9^

PROGRESS is a newly validated PROM for feminizing genital reconstructive surgery, with a dedicated sexual function domain showing high internal consistency. It assesses functional improvement, including sexual satisfaction, and is suitable for standardized outcome measurement. However, as a newer instrument, its content validity and clinical utility for sexual satisfaction specifically require further study, though initial results are promising. ^10^

GENDER-Q is an internationally validated, gender-affirming care–specific PROM. The Sexual Well-being Scale assesses overall sexual well-being, including aspects of sexual confidence, comfort, and satisfaction, demonstrating robust psychometric properties and test-retest reliability in a large, international sample. However, the scale does not specifically assess satisfaction with the function of surgically created genitals; only one item in the Vagina scale directly addresses sexual satisfaction after feminizing genital surgery. Thus, it may not fully capture the core outcome as defined and does not allow full application across the entire scope of the COS.

# Table S13. Extended results and quotes from feminizing and masculinizing COS consensus survey.

| **Outcome** | **OMI** | **Agree % (n)** | **Mostly agree % (n)** | **Quotes for “mostly agree”** | **Disagree % (n)** | **Quotes for “disagree”** |
| --- | --- | --- | --- | --- | --- | --- |
| Loss of neo-vaginal tissue lining | Physical examination using a speculum | 61 (19) | 39 (12) | Would recommend objectively measuring length in cm lost versus using <1/2, <2/3 etc. In addition that would mean intra-operatively the length should be documented. (3x) | 0 |  |
|  |  |  |  | It’s very difficult to measure the degree of loss, and be precise in the clinic real life. (2x) |  |  |
|  |  |  |  | Maybe make the division up in 4ths, so loss of tissue lining 1/4 etc. Mostly there is loss of the distal part / top, which is more likely loss of 1/4 instead of 1/3 |  |  |
|  |  |  |  | Not only report on length of vaginal tissue loss (1/3 - 1/3-2/3 - > 2/3) but also on the extent of circumferential tissue loss. Clinically, it makes a difference whether the reported vaginal tissue loss affects the entire circumference or only a small area of the circumference. |  |  |
|  |  |  |  | Measuring the proportion of the lost canal won´t add any benefit and might make confusions. IF there is a partial or total loss of the canal, the treatment will be surgical, so it would be graded accordingly through Clavien Dindo´s POC classification |  |  |
| Stricture of the neo-vaginal introitus | Physical examination | 77 (24) | 19 (6) | We should standardize some caliber. | 3 (1) | I am unclear as to the wording ('skin bridge') Does this refer to introital stenosis? |
|  |  |  |  | I would specify that this is JUST the introitus, so that the vaginal canal is open and accessible, except for the introital problem. |  |  |
| Neo-vaginal stenosis | Clinical history for stenosis-related symptoms AND Physical examination using a speculum AND dilator insertion | 74 (23) | 23 (7) | Would recommend objectively measuring length in cm as well as commenting on vaginal diameter (3x) | 3 (1) | It would be the same as canal loss, I suggest joining both |
|  |  |  |  | At clinical history maybe add the post operatively dilation schedule of the patient |  |  |
|  |  |  |  | Classification: CTCAE/Scielo Brasil |  |  |
| Rectovaginal fistula | Physical examination: IF INCONSCLUSIVE: additional imaging | 74 (23) | 26 (8) | I would add in timing intraoperatively (2x) | 0 |  |
|  |  |  |  | Will all practitioners be able to delineate where the dentate line is? Maybe we just keep it as cm from the introits? |  |  |
|  |  |  |  | I would say that the number of cm's from the dentate line is a valuable measurement, but to include it in the COS seems a little too much. |  |  |
|  |  |  |  | Should also include size in cm of the defect |  |  |
| Health-related quality of life | PROMIS scale: Global health | 90 (28) | 6 (2) |  | 3 (1) |  |
| Satisfaction with surgical result | GENDER-Q scale: Treatment outcome | 94 (29) | 6 (2) |  | 0 |  |
| Satisfaction with aesthetic outcome | GENDER-Q scales: Labia and clitoris | 94 (29) | 6 (2) |  | 0 |  |
| Satisfaction with neo-genital sexual function | No instrument (for entire scope): GENDER-Q scale: Vagina (if vaginoplasty procedure) | 87 (27) | 13 (4) |  | 0 |  |
| Erogenous sensibility of the genitals | No instrument | 84 (26) | 16 (5) | You will have to suggest a way for a clinician to measure this outcome in order to record it in the COS. You can stipulate that this is limited and further research is required but the clinicians will still require direction or will not use the outcome at all. | 0 |  |
|  |  |  |  | GENDER-Q sexual well-being, Orgasm and Vagina scales do ask relevant questions. With no specific other instruments, I'd suggest mentioning these scales as the closest available option. |  |  |
| Genital gender congruence | No instrument | 84 (26) | 13 (4) |  | 3 (1) |  |
| Neo-urethral stricture | Uroflowmetry AND Ultrasound post-void residual AND Retrograde urethrography AND Voiding cystourethrography | 75 (24) | 19 (6) |  | 6 (2) |  |
| Neo-urethral fistula | Physical examination AND Retrograde urethrography AND Voiding cystourethrography | 69 (22) | 25 (8) | Voiding cystourethrography should be optional or dropped (4x) | 6 (2) | RUG no longer considered necessary, optional urethrocystoscopy |
|  |  |  |  | Urethrocystoscopy should be added as option (3x) |  |  |
|  |  |  |  | The how is too complicated here |  |  |
| Flap necrosis of the neo-phallus | Physical examination | 88 (28) | 13 (4) | Need to specify that it is the final level of full necrosis not an interim assessment | 0 |  |
|  |  |  |  | Urethral loss can occur independent of shaft loss so this is very difficult to assess without a cystoscopy. Also what will we do if a patient has no UL but has a meatus and there is loss there? |  |  |
| Sensibility in the neo-phallus | GENDER-Q scale: Pensi sensation | 91 (29) | 3 (1) |  | 6 (2) |  |
| Ability to achieve orgasm | GENDER-Q scale: Orgasm | 94 (30) | `6 (2) |  | 0 |  |
| Sexual well-being | GENDER-Q scale: Sexual well-being | 91 (29) | 9 (3) |  | 0 |  |
| Satisfaction with neo-genital aesthetic result | GENDER-Q scales: Penis, Scrotum, Glans (if applicable) | 91 (29) | 9 (3) |  | 0 |  |
| Donor site morbidity | GENDER-Q scale: Donor site – Adverse effects | 88 (28) | 13 (4) |  | 0 |  |
| Ability to void in a standing position | Novel questionnaire | 81 (26) | 19 (6) | The questions have good face validity, but I expect some questions to be influenced by factors other than the ABILIT to void standing (eg availability of urinals, safety in public toilets). Wouldn't it make sense to survey only the ability for this outcome? | 3 (1) |  |
| Additional surgery | Clinical documentation | 87 (27) | 13 (4) | Include as a subsection the need for revision for acute complications | 0 |  |
|  |  |  |  | CD score toevoegen ihgv complicatie (“Add CD when adverse event”) |  |  |
|  |  |  |  | The how is too complicated here |  |  |
| Time of assessment for clinical outcomes & adverse events included in the COS | At least 12 months post-primary surgery | 77 (24) | 23 (7) |  | 0 |  |
| Time of assessment for all patient-reported outcomes included in the COS | 12 months post-primary surgery | 71 (22) | 29 (9) |  | 0 |  |

POC: post-operative complication; CTCAE: Common Terminology Criteria for Adverse Events; COS: core outcome set; PROMIS: Patient-Reported Outcome Measure Information System; RUG: retrograde urethrogram; UL: urethral lengthening; CD: Clavien-Dindo.

# Supplementary S14. Final consensus survey.

**Demographics**

1. **Consent**

“I consent to the collection of my demographic data for this study and its anonymous use for the examination and reporting of study results. I authorize the research team to securely retain this data on their institution’s server for five years, after which it will be permanently deleted. I confirm that I have no remaining questions regarding participation or data processing, or that any questions I had were answered by the study team after I reached out via the invitation email.”

- ☐ Yes
- ☐ No

1. **Gender identity**
   Please select the category that best represents your gender identity:

- ☐ Agender
- ☐ Cisgender woman
- ☐ Cisgender man
- ☐ Genderqueer
- ☐ Non-binary
- ☐ Transgender woman
- ☐ Transgender man
- ☐ Other / not listed (please specify): ____________

1. **Country of residence**
   Free text
2. **Age category**
   Please select your age category:

- ☐ 18–24 years old
- ☐ 25–34 years old
- ☐ 35–44 years old
- ☐ 45–54 years old
- ☐ 55–64 years old
- ☐ 65+ years old

1. **Professional experience in gender-affirming healthcare**
   Please select the category that best represents the duration of your professional experience in gender-affirming healthcare:

- ☐ 2 years or less
- ☐ 3–5 years
- ☐ 6–9 years
- ☐ 10–19 years
- ☐ More than 20 years

1. **Clinical experience in genital gender-affirming surgery**
   Please select the category that best represents your level of clinical experience:

- ☐ Recently completed surgical training
- ☐ Early career surgeon
- ☐ Moderately experienced surgeon
- ☐ Experienced surgeon
- ☐ Highly skilled surgeon

1. **Research experience in genital gender-affirming surgery**
   Please select the category that best represents your level of research experience:

- ☐ Little to no research experience
- ☐ Limited research experience
- ☐ Moderate research experience
- ☐ Experienced researcher
- ☐ Expert researcher

1. **Acknowledgment preference**
   If you wish to be named as a contributor in the acknowledgement section of publications resulting from this study, please state your full name below. If you wish to remain anonymous, you may leave this blank.
   Free text

**Outcome measurement proposals for mgGAS**

For each **core outcome**, please indicate whether you agree that the proposed measurement instruments and required reporting represent the *minimum necessary assessment and reporting to enable comparison in research*.
Rating options:

- AGREE
- MOSTLY AGREE *(with minor comments)*
- DISAGREE *(please explain your reasons)*

For each outcome, include a **comments box**:

“Please specify your minor modifications or explain your reason for disagreeing.”

**Additional surgery**

Please indicate whether you agree that this proposal represents the minimum necessary assessment and reporting to enable comparison of the core outcome in research.

| **Outcome assessment method** | **Required reporting** |
| --- | --- |
| **Clinical documentation** | **Characteristics:**   - Primary reason for additional surgery:   1. Patient initiated (dissatisfaction/unmet expectations)      1. Functional      2. Aesthetic      3. Combination functional & aesthetic   2. Healthcare provider initiated (adverse event/medically necessary) - Type of additional surgery - If multiple, sequential numbering - Time of additional surgery reported as number of days (up to 14 days), or weeks (2–12 weeks), or months (≥3 months) post-primary surgery |

**Rating:**

- ☐ Agree
- ☐ Mostly agree (minor comments)
- ☐ Disagree (with reasons)

Please specify your minor modifications or explain your reason for disagreeing:

- **Comment box:** Free text

**Neo-urethral stricture**

Please indicate whether you agree that this proposal represents the minimum necessary assessment and reporting to enable comparison of the core outcome in research.

| **Outcome assessment method** | **Required reporting** | **Classification** |
| --- | --- | --- |
| **Voiding assessment**  1. Uroflowmetry  **AND**  2. Ultrasound post-void residual volume | **Characteristics:**   - Q max (mL/sec) - Residual volume (mL) | Graded according to Clavien–Dindo |
| **Stricture assessment**  3. Retrograde urethrography  **AND**  4. Voiding cystourethrography | **Characteristics:**   - Number of strictures - Location(s): native, proximal anastomosis, pars fixa, distal anastomosis, and/or phallic part of neo-urethra - Length(s) (cm) - Time of diagnosis: weeks (≤12) or months (≥3) post-primary surgery | Graded according to Clavien–Dindo |

**Rating:**

- ☐ Agree
- ☐ Mostly agree (minor comments)
- ☐ Disagree (with reasons)

Please specify your minor modifications or explain your reason for disagreeing:

- **Comment box:** Free text

**Neo-urethral fistula**

Please indicate whether you agree that this proposal represents the minimum necessary assessment and reporting to enable comparison of the core outcome in research.

| **Outcome assessment method** | **Required reporting** | **Classification** |
| --- | --- | --- |
| 1. Physical examination  **AND**  2. Retrograde urethrography  **AND**  3. Voiding cystourethrography | **Characteristics:**   - Number of fistulae - Location(s): native, proximal anastomosis, pars fixa, distal anastomosis, phallic part of neo-urethra - Length(s) (cm) - Time of diagnosis: weeks (≤12) or months (≥3) post-primary surgery | Graded according to Clavien–Dindo |

**Rating:**

- ☐ Agree
- ☐ Mostly agree (minor comments)
- ☐ Disagree (with reasons)

Please specify your minor modifications or explain your reason for disagreeing:

- **Comment box:** Free text

**Flap necrosis of the neo-phallus**

Please indicate whether you agree that this proposal represents the minimum necessary assessment and reporting to enable comparison of the core outcome in research.

| **Outcome assessment method** | **Required reporting** | **Classification** |
| --- | --- | --- |
| **Physical examination** | **Complete flap necrosis:**   - Specify if after primary/secondary surgery - Involvement of flap used for neo-urethra - Time of diagnosis: days (≤14), weeks (2–12), or months (≥3)   **Partial flap necrosis:**   - Location (proximal/distal) - Depth (superficial/full-thickness) - Proportion (% of skin flap area, measured in cm²) - Specify if after primary/secondary surgery - Involvement of flap used for neo-urethra - Time of diagnosis: days, weeks, or months as above | Graded according to Clavien–Dindo |

**Rating:**

- ☐ Agree
- ☐ Mostly agree (minor comments)
- ☐ Disagree (with reasons)

Please specify your minor modifications or explain your reason for disagreeing:

- **Comment box:** Free text

**Time of assessment for clinical and adverse events**

Please indicate whether you agree with the proposed time of assessment of at least 12 months post-primary surgery for all clinical- and adverse event core outcomes.

| **Proposal** |
| --- |
| **Time of assessment for clinical outcomes & adverse events included in the COS** **The proposed required clinical evaluation period is set at a minimum of 12 months after primary surgery*** for all clinical outcomes and adverse events.  This is a minimum; additional time points are optional.*In cases where a staged approach is used for the surgical procedures within the scope of the COS, the final stage marks the completion of the primary surgery. From that point, the proposed minimum 12-month clinical evaluation period begins.* |
| **Rationale:** This period reflects the typical timeframe for clinical follow-up and captures early and later-onset adverse events/clinical outcomes. While some outcomes may also occur beyond this time point, extending the assessment period further may limit feasibility of the Core Outcome Set (COS). This proposal offers a balanced approach between comprehensive outcome capture and practical, unified application of the COS. |

**Rating:**

- ☐ Agree
- ☐ Mostly agree (minor comments)
- ☐ Disagree (with reasons)

Please specify your minor modifications or explain your reason for disagreeing:

- **Comment box:** Free text

**Sensibility in the neo-phallus**

Please indicate whether you agree that this proposal represents the minimum necessary assessment and reporting to enable comparison of the core outcome in research.

| **Outcome measurement instrument** | **Required reporting** |
| --- | --- |
| **GENDER-Q Scale: Penis sensation** | Converted Rasch score |

**Rating:**

- ☐ Agree
- ☐ Mostly agree (minor comments)
- ☐ Disagree (with reasons)

Please specify your minor modifications or explain your reason for disagreeing:

- **Comment box:** Free text

**Ability to achieve orgasm**

Please indicate whether you agree that this proposal represents the minimum necessary assessment and reporting to enable comparison of the core outcome in research.

| **Outcome measurement instrument** | **Required reporting** |
| --- | --- |
| **GENDER-Q Scale: Orgasm** | Converted Rasch score |

**Rating:**

- ☐ Agree
- ☐ Mostly agree (minor comments)
- ☐ Disagree (with reasons)

Please specify your minor modifications or explain your reason for disagreeing:

- **Comment box:** Free text

**Sexual well-being**

Please indicate whether you agree that this proposal represents the minimum necessary assessment and reporting to enable comparison of the core outcome in research.

| **Outcome measurement instrument** | **Required reporting** |
| --- | --- |
| **GENDER-Q Scale: Sexual well-being** | Converted Rasch score |

**Rating:**

- ☐ Agree
- ☐ Mostly agree (minor comments)
- ☐ Disagree (with reasons)

Please specify your minor modifications or explain your reason for disagreeing:

- **Comment box:** Free text

**Satisfaction with neo-genital aesthetic result**

Please indicate whether you agree that this proposal represents the minimum necessary assessment and reporting to enable comparison of the core outcome in research.

| **Outcome measurement instrument** | **Required reporting** |
| --- | --- |
| **GENDER-Q Scale: Penis**  AND, if applicable:  **GENDER-Q Scale: Scrotum**  AND, if applicable:  **GENDER-Q Scale: Glans** | Converted Rasch score per applicable scale |

**Rating:**

- ☐ Agree
- ☐ Mostly agree (minor comments)
- ☐ Disagree (with reasons)

Please specify your minor modifications or explain your reason for disagreeing:

- **Comment box:** Free text

**Donor site morbidity**

Please indicate whether you agree that this proposal represents the minimum necessary assessment and reporting to enable comparison of the core outcome in research.

| **Outcome measurement instrument** | **Required reporting** |
| --- | --- |
| **GENDER-Q Scale: Donor site – Adverse effects** | Converted Rasch score |

**Rating:**

- ☐ Agree
- ☐ Mostly agree (minor comments)
- ☐ Disagree (with reasons)

Please specify your minor modifications or explain your reason for disagreeing:

- **Comment box:** Free text

**Ability to void in a standing position**

Please indicate whether you agree that this proposal represents the minimum necessary assessment and reporting to enable comparison of the core outcome in research.

| **Outcome measurement instrument** | **Required reporting** | | | |
| --- | --- | --- | --- | --- |
| **Novel questionnaire: Urinate while standing** | Score per item | | | |
| These questions ask about your ability to URINATE WHILE STANDING.  Please answer thinking of the past week. How much do you disagree or agree with each statement? | | | | |
|  | **DISAGREE** | **SLIGHTLY AGREE** | **MOSTLY AGREE** | **STRONGLY AGREE** |
| **1.** I am able to pee while standing with control over the stream, through an unzipped fly. | 1 | 2 | 3 | 4 |
| **2.** I am comfortable using a public urinal to pee while standing. | 1 | 2 | 3 | 4 |
| **3.** When I pee, it is usually while standing. | 1 | 2 | 3 | 4 |
| **4.** When I pee, it is usually **without** the use of a device. | 1 | 2 | 3 | 4 |

**Rating:**

- ☐ Agree
- ☐ Mostly agree (minor comments)
- ☐ Disagree (with reasons)

Please specify your minor modifications or explain your reason for disagreeing:

- **Comment box:** Free text

**Time of assessment for patient-reported outcomes**

Please indicate whether you agree with the proposed time of questionnaire administration—12 months post-primary surgery—for all patient-reported core outcomes.

| **Proposal** |
| --- |
| **Time of assessment for patient-reported outcomes included in the COS** **The proposed time of assessment is set at 12 months post-primary surgery*** for all patient-reported outcomes (PROs) included in the COS.  This is a minimum; additional time points are optional.*In cases where a staged approach is used for the surgical procedures within the scope of the COS, the final stage marks the completion of the primary surgery. From that point, the proposed time of assessment is determined.* |
| **Rationale:** Aligning this timing across PROs simplifies questionnaire administration and use of the COS. While one year may not be sufficient to capture all personal experiences, this timeframe balances adequate time post-primary surgery with feasibility. We also recognize global differences in healthcare systems and aim to avoid imposing excessive assessment periods. |

**Rating:**

- ☐ Agree
- ☐ Mostly agree (minor comments)
- ☐ Disagree (with reasons)

Please specify your minor modifications or explain your reason for disagreeing:

- **Comment box:** Free text

**Final comments**

If you have any additional questions or final comments, please share them below.

- **Comment box:** Free text

**Outcome measurement proposals for fgGAS**

For each **core outcome**, please indicate whether you agree that the proposed measurement instruments and required reporting represent the *minimum necessary assessment and reporting to enable comparison in research*.
Rating options:

- AGREE
- MOSTLY AGREE *(with minor comments)*
- DISAGREE *(please explain your reasons)*

For each outcome, include a **comments box**:

“Please specify your minor modifications or explain your reason for disagreeing.”

**Loss of neo-vaginal tissue lining**

Please indicate whether you agree that this proposal represents the minimum necessary assessment and reporting to enable comparison of the core outcome in research.

| **Outcome assessment method** | **Required reporting** | **Classification** |
| --- | --- | --- |
| **Physical examination using a speculum** | **Complete loss of lining characteristics:**   - Time of diagnosis: weeks (≤12) or months (≥3) post-primary surgery   **Partial loss of lining characteristics:**   - Proportion of canal lining length lost:   - <1/3   - 1/3–2/3   - >2/3 - Time of diagnosis: weeks (≤12) or months (≥3) post-primary surgery | Graded according to Clavien–Dindo |

**Rating:**

- ☐ Agree
- ☐ Mostly agree (minor comments)
- ☐ Disagree (with reasons)

Please specify your minor modifications or explain your reason for disagreeing:

- **Comment box:** Free text

**Stricture of the neo-vaginal introitus**

Please indicate whether you agree that this proposal represents the minimum necessary assessment and reporting to enable comparison of the core outcome in research.

| **Outcome assessment method** | **Required reporting** | **Classification** |
| --- | --- | --- |
| **Physical examination** | **Characteristics:**   - Time of diagnosis: weeks (≤12) or months (≥3) post-primary surgery | Graded according to Clavien–Dindo |

**Rating:**

- ☐ Agree
- ☐ Mostly agree (minor comments)
- ☐ Disagree (with reasons)

Please specify your minor modifications or explain your reason for disagreeing:

- **Comment box:** Free text

**Neo-vaginal stenosis**

Please indicate whether you agree that this proposal represents the minimum necessary assessment and reporting to enable comparison of the core outcome in research.

| **Outcome assessment method** | **Required reporting** | **Classification** |
| --- | --- | --- |
| **Symptom assessment** 1. Clinical history for stenosis-related symptoms  **AND** | - Nature of symptoms (open) | Graded according to Clavien–Dindo |
| **Stenosis assessment** 2. Physical examination using a speculum **AND**  3. dilator insertion | - Location: vault or canal (except introitus) - Time of diagnosis: weeks (≤12) or months (≥3) post-primary surgery | Graded according to Clavien–Dindo |

**Rating:**

- ☐ Agree
- ☐ Mostly agree (minor comments)
- ☐ Disagree (with reasons)

Please specify your minor modifications or explain your reason for disagreeing:

- **Comment box:** Free text

**Rectovaginal fistula**

Please indicate whether you agree that this proposal represents the minimum necessary assessment and reporting to enable comparison of the core outcome in research.

| **Outcome assessment method** | **Required reporting** | **Classification** |
| --- | --- | --- |
| 1. Physical examination  **IF INCONCLUSIVE:** 2. Additional imaging | - Location(s): in the rectum, as distance in cm from the dentate line - Time of diagnosis: weeks (≤12) or months (≥3) post-primary surgery | Graded according to Clavien–Dindo |

**Rating:**

- ☐ Agree
- ☐ Mostly agree (minor comments)
- ☐ Disagree (with reasons)

Please specify your minor modifications or explain your reason for disagreeing:

- **Comment box:** Free text

**Time of assessment for clinical outcomes & adverse events**

Please indicate whether you agree with the proposed time of assessment of at least 12 months post-primary surgery for all clinical- and adverse event core outcomes.

| **Proposal** |
| --- |
| **The proposed required clinical evaluation period is set at a minimum of 12 months after primary surgery*** for all clinical outcomes and adverse events. This is a minimum; additional time points are optional.  *In cases where a staged approach is used, the final stage marks the completion of the primary surgery. From that point, the proposed minimum 12-month clinical evaluation period begins.* |
| **Rationale:** This timeframe reflects typical clinical follow-up and captures early and later-onset outcomes while maintaining feasibility for COS implementation. |

**Rating:**

- ☐ Agree
- ☐ Mostly agree (minor comments)
- ☐ Disagree (with reasons)

Please specify your minor modifications or explain your reason for disagreeing:

- **Comment box:** Free text

**Health-related quality of life**

Please indicate whether you agree that this proposal represents the minimum necessary assessment and reporting to enable comparison of the core outcome in research.

| **Outcome measurement instrument** | **Required reporting** |
| --- | --- |
| **PROMIS scale: Global Health** | Calculated T-score and Standard Error (SE) |

**Rating:**

- ☐ Agree
- ☐ Mostly agree (minor comments)
- ☐ Disagree (with reasons)

Please specify your minor modifications or explain your reason for disagreeing:

- **Comment box:** Free text

**Satisfaction with surgical result**

Please indicate whether you agree that this proposal represents the minimum necessary assessment and reporting to enable comparison of the core outcome in research.

| **Outcome measurement instrument** | **Required reporting** |
| --- | --- |
| **GENDER-Q scale: Treatment outcome** | Converted Rasch score |

**Rating:**

- ☐ Agree
- ☐ Mostly agree (minor comments)
- ☐ Disagree (with reasons)

Please specify your minor modifications or explain your reason for disagreeing:

- **Comment box:** Free text

**Satisfaction with aesthetic outcome**

Please indicate whether you agree that this proposal represents the minimum necessary assessment and reporting to enable comparison of the core outcome in research.

| **Outcome measurement instrument** | **Required reporting** |
| --- | --- |
| **GENDER-Q scales: Labia and Clitoris** | Converted Rasch score per scale |

**Rating:**

- ☐ Agree
- ☐ Mostly agree (minor comments)
- ☐ Disagree (with reasons)

Please specify your minor modifications or explain your reason for disagreeing:

- **Comment box:** Free text

**Satisfaction with neo-genital sexual function**

Please indicate whether you agree that this proposal represents the minimum necessary assessment and reporting to enable comparison of the core outcome in research.

| **Outcome measurement instrument** | **Required reporting** |
| --- | --- |
| All procedures (entire scope): **No instrument available** |  |
| If vaginoplasty procedure (with vaginal canal): **GENDER-Q scale: Vagina** | Converted Rasch score |

**Rating:**

- ☐ Agree
- ☐ Mostly agree (minor comments)
- ☐ Disagree (with reasons)

Please specify your minor modifications or explain your reason for disagreeing:

- **Comment box:** Free text

**Erogenous sensibility of the genitals**

Please indicate whether you agree that this proposal represents the minimum necessary assessment and reporting to enable comparison of the core outcome in research.

| **Outcome measurement instrument** | **Required reporting** |
| --- | --- |
| No instrument available | — |

**Rating:**

- ☐ Agree
- ☐ Mostly agree (minor comments)
- ☐ Disagree (with reasons)

Please specify your minor modifications or explain your reason for disagreeing:

- **Comment box:** Free text

**Genital gender congruence**

Please indicate whether you agree that this proposal represents the minimum necessary assessment and reporting to enable comparison of the core outcome in research.

| **Outcome measurement instrument** | **Required reporting** | | | |
| --- | --- | --- | --- | --- |
| **Novel questionnaire: Genital gender congruence** | Score per item | | | |
| These questions ask about **GENITAL GENDER CONGRUENCE**. Please answer thinking of the past week. How much do you disagree or agree with each statement? | | | | |
|  | **DISAGREE** | **SLIGHTLY AGREE** | **MOSTLY AGREE** | **STRONGLY AGREE** |
| 1. I feel that my genitals match my gender identity. | 1 | 2 | 3 | 4 |
| 2. My genitals make me feel affirmed in my gender. | 1 | 2 | 3 | 4 |

**Rating:**

- ☐ Agree
- ☐ Mostly agree (minor comments)
- ☐ Disagree (with reasons)

Please specify your minor modifications or explain your reason for disagreeing:

- **Comment box:** Free text

**Time of assessment for patient-reported outcomes**

Please indicate whether you agree with the proposed time of questionnaire administration—12 months post-primary surgery—for all patient-reported core outcomes.

| **Proposal** |
| --- |
| **The proposed time of assessment is set at 12 months post-primary surgery*** for all patient-reported outcomes (PROs) included in the COS. This is a minimum; additional time points are optional.  *In cases where a staged approach is used, the final stage marks the completion of the primary surgery. From that point, the proposed time of assessment is determined.* |
| **Rationale:** Aligning this timing across PROs simplifies questionnaire administration and use of the COS. While one year may not be sufficient to capture all personal experiences, this timeframe balances adequate time post-primary surgery with feasibility. We also recognize global differences in healthcare systems and aim to avoid imposing excessive assessment periods. |

**Rating:**

- ☐ Agree
- ☐ Mostly agree (minor comments)
- ☐ Disagree (with reasons)

Please specify your minor modifications or explain your reason for disagreeing:

- **Comment box:** Free text

**Final comments**

If you have any additional questions or final comments, please share them below.

- **Comment box:** Free text

# Supplementary S15. Full search strategy.

**Search strategy for Ovid/Medline (12 January 2025)**

| **1** | (metoidioplast* or metaidioplast* or phalloplast* or scrotoplast* or neo-phall* or neophall* or coronaplast* or corona-plast* or glans-plast* or glansplast* or (masculin* adj3 (genitoplast* or genital*))).ti,ab,kf. | 1,179 |
| --- | --- | --- |
| **2** | ((penile or penis) adj3 reconstruct*).ti,ab,kf. | 803 |
| **3** | exp Sex Reassignment Procedures/ or exp Gender Dysphoria/ or exp Transgender Persons/ or exp Transsexualism/ or (gender-disorder* or gender-identity-disorder* or gender-dysphor* or sexual-dysphor* or sex-dysphor* or gender-incongruen* or transgender* or trans-gender* or transsex* or trans-sex* or sex-reassign* or sex-change* or gender-reassign* or gender-chang* or gender-affirm* or transwom* or trans-wom* or transman or transmen or trans-man or trans-men or transmale* or trans-male* or female-to-male or assigned-female-at-birth or assigned-female-sex or biological female* or biological-women or transboy* or trans-boy* or gender-divers*).ti,ab,kf. | 58,616 |
| **4** | (1 or 2) and 3 | 595 |

**Search strategy for Embase.com (12 January 2025)**

| **No.** | **Query** | **Results** |
| --- | --- | --- |
| **#5** | #3 NOT #4 | 7**57** |
| **#4** | #3 AND ('conference abstract'/it OR 'conference paper'/it OR 'conference review'/it) | 3**62** |
| **#3** | #1 AND #2 | 1,119 |
| **#2** | 'sex reassignment'/exp OR 'gender dysphoria'/exp OR 'transgender'/de OR 'female to male transgender'/exp OR 'gender disorder*':ti,ab,kw OR 'gender identity disorder*':ti,ab,kw OR 'gender dysphor*':ti,ab,kw OR 'sexual dysphor*':ti,ab,kw OR 'sex dysphor*':ti,ab,kw OR 'gender incongruen*':ti,ab,kw OR transgender*:ti,ab,kw OR 'trans gender*':ti,ab,kw OR transsex*:ti,ab,kw OR 'trans sex*':ti,ab,kw OR 'sex reassign*':ti,ab,kw OR 'sex change*':ti,ab,kw OR 'gender reassign*':ti,ab,kw OR 'gender chang*':ti,ab,kw OR 'gender affirm*':ti,ab,kw OR transwom*:ti,ab,kw OR 'trans wom*':ti,ab,kw OR transman:ti,ab,kw OR transmen:ti,ab,kw OR 'trans man':ti,ab,kw OR 'trans men':ti,ab,kw OR transmale*:ti,ab,kw OR 'trans male*':ti,ab,kw OR 'female to male':ti,ab,kw OR 'assigned female at birth':ti,ab,kw OR 'assigned female sex':ti,ab,kw OR 'biological female*':ti,ab,kw OR 'biological women':ti,ab,kw OR transboy*:ti,ab,kw OR 'trans boy*':ti,ab,kw OR 'gender divers*':ti,ab,kw | 40,264 |
| **#1** | 'metoidioplasty'/exp OR 'scrotoplasty'/exp OR 'phalloplasty'/exp OR 'neophallus'/exp OR 'glansplasty'/exp OR 'penis reconstruction'/exp OR metoidioplast*:ti,ab,kw OR metaidioplast*:ti,ab,kw OR phalloplast*:ti,ab,kw OR scrotoplast*:ti,ab,kw OR 'neo phall*':ti,ab,kw OR neophall*:ti,ab,kw OR coronaplast*:ti,ab,kw OR 'corona plast*':ti,ab,kw OR 'glans plast*':ti,ab,kw OR glansplast*:ti,ab,kw OR ((masculin* NEAR/3 (genitoplast* OR genital*)):ti,ab,kw) OR (((penile OR penis) NEAR/3 reconstruct*):ti,ab,kw) | 3,972 |

**Search strategy for Clarivate Analytics/Web of Science Core Collection (12 January 2025)**

| **#3** | #1 AND #2 | 7**09** |
| --- | --- | --- |
| **#2** | TS=("gender-disorder*" OR "gender-identity-disorder*" OR "gender-dysphor*" OR "sexual-dysphor*" OR "sex-dysphor*" OR "gender-incongruen*" OR "transgender*" OR "trans-gender*" OR "transsex*" OR "trans-sex*" OR "sex-reassign*" OR "sex-change*" OR "gender-reassign*" OR "gender-chang*" OR "gender-affirm*" OR "transwom*" OR "trans-wom*" OR "transman" OR "transmen" OR "trans-man" OR "trans-men" OR "transmale*" OR "trans-male*" OR "female-to-male" OR "assigned-female-at-birth" OR "assigned-female-sex" OR "biological-female*" OR "biological-women" OR "transboy*" OR "trans-boy*" OR "gender-divers*") | 4**4,781** |
| **#1** | TS=("metoidioplast*" OR "metaidioplast*" OR "phalloplast*" OR "scrotoplast*" OR "neo-phall*" OR "neophall*" OR "coronaplast*" OR "corona-plast*" OR "glans-plast*" OR "glansplast*" OR ("masculin*" NEAR/3 ("genitoplast*" OR "genital*")) OR (("penile" OR "penis") NEAR/3 "reconstruct*")) | 2,1**60** |

**Search strategy for Elsevier/Scopus (12 January 2025)**

| **History Count** | **Search Terms** | **Results** |
| --- | --- | --- |
| **#3** | **#1 AND #2** | 8**46** |
| **#2** | **TITLE-ABS-KEY (("gender-disorder*" OR "gender-identity-disorder*" OR "gender-dysphor*" OR "sexual-dysphor*" OR "sex-dysphor*" OR "gender-incongruen*" OR "transgender*" OR "trans-gender*" OR "transsex*" OR "trans-sex*" OR "sex-reassign*" OR "sex-change*" OR "gender-reassign*" OR "gender-chang*" OR "gender-affirm*" OR "transwom*" OR "trans-wom*" OR "transman" OR "transmen" OR "trans-man" OR "trans-men" OR "transmale*" OR "trans-male*" OR "female-to-male" OR "assigned-female-at-birth" OR "assigned-female-sex" OR "biological-female*" OR "biological-women" OR "transboy*" OR "trans-boy*" OR "gender-divers*"))** | **55,828** |
| **#1** | **TITLE-ABS-KEY (("metoidioplast*" OR "metaidioplast*" OR "phalloplast*" OR "scrotoplast*" OR "neo-phall*" OR "neophall*" OR "coronaplast*" OR "corona-plast*" OR "glans-plast*" OR "glansplast*" OR ("masculin*" W/3 ("genitoplast*" OR "genital*")) OR (("penile" OR "penis") W/3 "reconstruct*")))** | **2,901** |

**Search strategy for Ebsco/APA PsycINFO (12 January 2025)**

| **#** | **Query** | **Results** |
| --- | --- | --- |
| **S3** | **S1 AND S2** | **83** |
| **S2** | **DE "Transgender" OR DE "Gender Reassignment" OR DE "Gender Identity" OR DE "Gender Nonbinary" OR DE "Gender Nonconforming" OR DE "Transsexualism" OR DE "Gender Dysphoria" OR TI("gender-disorder*" OR "gender-identity-disorder*" OR "gender-dysphor*" OR "sexual-dysphor*" OR "sex-dysphor*" OR "gender-incongruen*" OR "transgender*" OR "trans-gender*" OR "transsex*" OR "trans-sex*" OR "sex-reassign*" OR "sex-change*" OR "gender-reassign*" OR "gender-chang*" OR "gender-affirm*" OR "transwom*" OR "trans-wom*" OR "transman" OR "transmen" OR "trans-man" OR "trans-men" OR "transmale*" OR "trans-male*" OR "female-to-male" OR "assigned-female-at-birth" OR "assigned-female-sex" OR "biological-female*" OR "biological-women" OR "transboy*" OR "trans-boy*" OR "gender-divers*") OR AB("gender-disorder*" OR "gender-identity-disorder*" OR "gender-dysphor*" OR "sexual-dysphor*" OR "sex-dysphor*" OR "gender-incongruen*" OR "transgender*" OR "trans-gender*" OR "transsex*" OR "trans-sex*" OR "sex-reassign*" OR "sex-change*" OR "gender-reassign*" OR "gender-chang*" OR "gender-affirm*" OR "transwom*" OR "trans-wom*" OR "transman" OR "transmen" OR "trans-man" OR "trans-men" OR "transmale*" OR "trans-male*" OR "female-to-male" OR "assigned-female-at-birth" OR "assigned-female-sex" OR "biological-female*" OR "biological-women" OR "transboy*" OR "trans-boy*" OR "gender-divers*") OR KW("gender-disorder*" OR "gender-identity-disorder*" OR "gender-dysphor*" OR "sexual-dysphor*" OR "sex-dysphor*" OR "gender-incongruen*" OR "transgender*" OR "trans-gender*" OR "transsex*" OR "trans-sex*" OR "sex-reassign*" OR "sex-change*" OR "gender-reassign*" OR "gender-chang*" OR "gender-affirm*" OR "transwom*" OR "trans-wom*" OR "transman" OR "transmen" OR "trans-man" OR "trans-men" OR "transmale*" OR "trans-male*" OR "female-to-male" OR "assigned-female-at-birth" OR "assigned-female-sex" OR "biological-female*" OR "biological-women" OR "transboy*" OR "trans-boy*" OR "gender-divers*")** | **49,312** |
| **S1** | **TI("metoidioplast*" OR "metaidioplast*" OR "phalloplast*" OR "scrotoplast*" OR "neo-phall*" OR "neophall*" OR "coronaplast*" OR "corona-plast*" OR "glans-plast*" OR "glansplast*" OR ("masculin*" N3 ("genitoplast*" OR "genital*")) OR (("penile" OR "penis") N3 "reconstruct*")) OR AB("metoidioplast*" OR "metaidioplast*" OR "phalloplast*" OR "scrotoplast*" OR "neo-phall*" OR "neophall*" OR "coronaplast*" OR "corona-plast*" OR "glans-plast*" OR "glansplast*" OR ("masculin*" N3 ("genitoplast*" OR "genital*")) OR (("penile" OR "penis") N3 "reconstruct*")) OR KW("metoidioplast*" OR "metaidioplast*" OR "phalloplast*" OR "scrotoplast*" OR "neo-phall*" OR "neophall*" OR "coronaplast*" OR "corona-plast*" OR "glans-plast*" OR "glansplast*" OR ("masculin*" N3 ("genitoplast*" OR "genital*")) OR (("penile" OR "penis") N3 "reconstruct*"))** | **141** |

**Search strategy for ProQuest/International Bibliography of Social Sciences (IBSS) (12 January 2025)**

| **#** | **Query** | **Results** |
| --- | --- | --- |
| **#3** | **#1 AND #2** | **753** |
| **#2** | **NOFT("gender-disorder*" OR "gender-identity-disorder*" OR "gender-dysphor*" OR "sexual-dysphor*" OR "sex-dysphor*" OR "gender-incongruen*" OR "transgender*" OR "trans-gender*" OR "transsex*" OR "trans-sex*" OR "sex-reassign*" OR "sex-change*" OR "gender-reassign*" OR "gender-chang*" OR "gender-affirm*" OR "transwom*" OR "trans-wom*" OR "transman" OR "transmen" OR "trans-man" OR "trans-men" OR "transmale*" OR "trans-male*" OR "female-to-male" OR "assigned-female-at-birth" OR "assigned-female-sex" OR "biological-female*" OR "biological-women" OR "transboy*" OR "trans-boy*" OR "gender-divers*")** | **68,504** |
| **#1** | **NOFT("metoidioplast*" OR "metaidioplast*" OR "phalloplast*" OR "scrotoplast*" OR "neo-phall*" OR "neophall*" OR "coronaplast*" OR "corona-plast*" OR "glans-plast*" OR "glansplast*" OR ("masculin*" NEAR/3 ("genitoplast*" OR "genital*"))) OR (("penile" OR "penis") NEAR/3 "reconstruct*")** | **2,596** |

**References**

1. Kaur MN, Morrison SD, Kennedy SL, et al. International study to develop a patient-reported outcome measure to evaluate outcomes of gender-affirming care - the GENDER-Q. Journal of Patient-Reported Outcomes. 2024;**8**(1):134.

2. Kennedy SL, Cornacchi SD, Kaur MN, et al. Development of a Patient-Reported Outcome Measure for Youth Receiving Gender-Affirming Care: The GENDER-Q Youth Module. Transgend Health. 2025;**10**(4):375–85.

3. Cella D, Yount S, Rothrock N, et al. The Patient-Reported Outcomes Measurement Information System (PROMIS): progress of an NIH Roadmap cooperative group during its first two years. Med Care. 2007;**45**(5 Suppl 1):S3–S11.

4. Cella D, Riley W, Stone A, et al. The Patient-Reported Outcomes Measurement Information System (PROMIS) developed and tested its first wave of adult self-reported health outcome item banks: 2005-2008. J Clin Epidemiol. 2010;**63**(11):1179–94.

5. Bevans M, Ross A, Cella D. Patient-Reported Outcomes Measurement Information System (PROMIS): efficient, standardized tools to measure self-reported health and quality of life. Nurs Outlook. 2014;**62**(5):339–45.

6. Sweitzer KR, Milek D, Escandon JM, Sanderson D, Christiano JG. Patient-Reported Outcome (PROMIS) Scores Improve After Gender-Affirming Mastectomy. Ann Plast Surg. 2025;**94**(3):281–5.

7. Vedovo F, Di Blas L, Perin C, et al. Operated Male-to-Female Sexual Function Index: Validity of the First Questionnaire Developed to Assess Sexual Function after Male-to-Female Gender Affirming Surgery. The Journal of Urology. 2020;**204**(1):115–20.

8. Cueto-Galdames B, Cifuentes M, Vedovo F, Sacomori C. Cross-Cultural Adaptation and Face Validation of the Chilean Version of the Operated Male to Female Sexual Function Index. International Urogynecology Journal. 2024;**35**(6):1271–80.

9. Huber S, Ferrando C, Safer JD, et al. Development and Validation of Urological and Appearance Domains of the Post-Affirming Surgery Form and Function Individual Reporting Measure (AFFIRM) for Transwomen following Genital Surgery. Journal of Urology. 2021;**206**(6):1445–53.

10. Kanthabalan A, Hosking Jervis F, Junejo MH, Morley R, Bellringer J, Rashid T. PROGRESS (Patient-Reported Outcomes in Genital Reconstructive Surgeries): A Validated Patient-Reported Outcome Measure Questionnaire to Assess Post-Operative Functional Improvement Following Feminising Genital Reconstructive Surgery. J Clin Med. 2025;**14**(8).
